# Comparisons of different new-generation transcatheter aortic valve implantation devices for patients with severe aortic stenosis: a systematic review and network meta-analysis

**DOI:** 10.1097/JS9.0000000000000456

**Published:** 2023-05-10

**Authors:** Yi-Xing Yang, Xin-Ming Liu, Yuan Fu, Chuang Li, Hong-Jiang Wang, Li Xu, Kun Xia, Zhi-Yong Zhang, Jiu-Chang Zhong, Mu-Lei Chen, Pi-Xiong Su, Le-Feng Wang

**Affiliations:** Heart Center and Beijing Key Laboratory of Hypertension, Beijing Chaoyang Hospital, Capital Medical University, Chaoyang District, Beijing, China

**Keywords:** aortic stenosis, network meta-analysis, new-generation, systematic review, transcatheter aortic valve implantation

## Abstract

**Background:**

Whether there are differences among the new-generation transcatheter aortic valve implantation (TAVI) devices for patients with aortic stenosis remains unclear. The aim of the study was to compare the efficiency and safety of different new-generation TAVI devices for patients with aortic stenosis.

**Materials and methods:**

A comprehensive search of PubMed, Embase and Web of Science from their inception to 1 February 2022. Randomized clinical trials and observational studies that compared two or more different TAVI devices were enroled. Pairwise meta-analysis and frequentist network meta-analysis were conducted to pool the outcome estimates of interest.

**Results:**

A total of 79 studies were finally included. According to the surface under the cumulative ranking, the top two ranked valves for lower rates of events were as follows: direct flow medical (DFM) (4.6%) and Lotus (48.8%) for lower rate of device success; Sapien 3 (16.8%) and DFM (19.7%) for lower mortality; DFM (8.6%) and Sapien 3 (25.5%) for lower rates of stroke; Evolut (27.6%) and DFM (35.8%) for lower rates of major and life-threatening bleeding; Portico (22.6%) and Sapien 3 (41.9%) for lower rates of acute kidney injury; Acurate (8.6%) and DFM (13.2%) for lower rates of permanent pacemaker implantation; Lotus (0.3%) and Sapien 3 (22.7%) for lower rates of paravalvular leak; Evolut (1.4%) and Portico (29.1%) for lower rates of mean aortic valve gradients.

**Conclusions:**

The findings of the present study suggested that the device success rates were comparable among these new-generation valves except for DFM. After excluding DFM, Sapien 3 might be the best effective for decreased mortality and stroke; Lotus might be the best effective for decreased paravalvular leak; Evolut might be the best effective for decreased major and life-threatening bleeding and mean aortic valve gradients; Acurate and Portico might be the best effective for decreased permanent pacemaker implantation and acute kidney injury, respectively.

## Introduction

Highlights

- The first network meta-analysis comparing new-generation valves for aortic stenosis.
- Device success rates are comparable among these valves except for direct flow medical.
- Each valve has its own unique advantages for the treatment of aortic stenosis.
- The non-metallic design of direct flow medical system is worthy of further exploration.

Transcatheter aortic valve implantation (TAVI) has been developed as an effective alternative to surgical aortic valve replacement for patients with symptomatic severe aortic stenosis (AS) who are at high, intermediate and even low surgical risk^1^. Within the earlier years, the self-expandable CoreValve and the balloon-expandable SAPIEN or SAPIEN XT valve are the most commonly used TAVI devices. However, these early generation prosthetic devices have some inherent shortcomings such as paravalvular leak, vascular complications and conduction abnormalities which significantly weaken the efficiency and safety of TAVI^2^.

Recently, in order to address the limitations of earlier-generation devices and to provide more favourable outcomes, multiple newer-generation TAVI devices with marked design improvements have been widely introduced into clinical practice, including the self-expandable valves (Evolut R/PRO, Acurate neo, Portico), balloon-expandable valve (Sapien 3), mechanically expandable valve (Lotus) and non-metallic valve [direct flow medical (DFM)]^3–5^. Whether there are differences among these newer-generation devices with regards to the clinical outcomes remains unclear. The objective of the study was to compare the efficiency and safety of different new-generation TAVI devices for AS patients by using a network meta-analysis.

## Methods

This systematic review was reported according to the PRISMA, Supplemental Digital Content 1, http://links.lww.com/JS9/A446, Supplemental Digital Content 2, http://links.lww.com/JS9/A447 (Preferred Reporting Items for Systematic Reviews and Meta-Analyses) and AMSTAR, Supplemental Digital Content 3, http://links.lww.com/JS9/A448 (Assessing the methodological quality of systematic reviews) guidelines^6–8^. The protocol of the study has been registered on PROSPERO (CRD42021224646).

### Search strategy and selection criteria

We conducted a systematic search of the literature on the PubMed, Embase and Web of Science from their inception to 1 February 2022. The detailed search terms and strategy are provided in the Supplementary Table 1, Supplemental Digital Content 4, http://links.lww.com/JS9/A449.

Studies were included if they met the following criteria: (1) study sample size greater than 30; (2) study comparing the in-hospital or/and 30-day outcomes among the new-generation TAVI devices including Evolut R and Pro (R/Pro), Acurate, Portico, Sapien 3, Lotus and DFM; (3) randomized clinical trial (RCT) or observational study; (4) English study.

Studies were excluded if they met the following criteria: (1) patients who had previous aortic valve replacement (valve-in-valve), bicuspid aortic valve or pure aortic regurgitation; (2) sample size in each treatment group less than 10; (3) study not comparing outcomes among the new-generation TAVI devices; (4) outcomes of interest were not clearly reported or impossible to extract from the published results; (5) case report, abstract and editorial; (6) non-English study.

Two independent reviewers screened the titles and abstracts of studies retrieved by the initial search. Then, full-text of the potentially relevant articles were evaluated by the selection criteria. Any discrepancies or disagreements were resolved by a third reviewer.

### Outcome measures and definitions

The primary endpoint was device success. The second endpoints included mortality, stroke, major or life-threatening bleeding (MLTB), major vascular complications (MVC), acute kidney injury (AKI) and permanent pacemaker implantation (PPI). Additionally, we also assessed the individual components of device success including the absence of procedural mortality, correct positioning of a single prosthetic heart valve into the proper anatomical location, no moderate-to-severe paravalvular leak (PVL), no prosthesis patient mismatch (PPM) and mean aortic valve gradients (MAVG). The definitions of all endpoints were in accordance with the Valve Academic Research Consortium-2 (VARC-2) criteria^9^.

### Data extraction and risk of bias assessment

Two investigators independently extracted data from each study using a standardized extraction form including first author, publication year, study design, sample size, types of TAVI devices, baseline clinical, imaging and procedural characteristics of patients, study outcomes. We tried to contact the authors by e-mails for the required data which was missing from the original published articles. Any discrepancies were resolved by consensus.

The quality of RCTs and observational studies were assessed by using the Cochrane Collaboration’s tool and Newcastle-Ottawa Scale, respectively^10,11^. Two reviewers performed the quality assessment individually. Discrepancies were resolved by team discussion.

### Data synthesis and statistical analysis

First, we conducted a pairwise meta-analysis in Review Manager (version 5.3) to provide direct estimates for the outcomes. The Mantel Haenszel method with the random-effects model was used to calculate odds ratios and 95% CIs for each endpoint. Heterogeneity was evaluated by visual inspection of the forest plot and tested using I^2^ statistics.

Next, we conducted a frequentist network meta-analysis in STATA software (version 14.0) by using the network command to generate indirect and mixed estimates for the endpoints. We assumed network consistency and a common heterogeneity parameter across all pairwise comparisons within an outcome. We used the between studies variance τ^2^ to present heterogeneity across the network. Estimates of τ^2^ of ~0.04, 0.16 and 0.36 are considered to represent a low, moderate and high degree of heterogeneity, respectively^12^.

We assessed the transitivity assumption by comparing the distribution or frequency of patient characteristics that modify treatment effects across treatment comparisons. We statistically evaluated inconsistencies between direct and indirect evidence within the network meta-analysis globally by using the design-by-treatment interaction model and locally by using the loop-specific approach and the node-splitting method^13^. We obtained a treatment hierarchy for each endpoint by using surface under the cumulative ranking (SUCRA) and mean ranks^14^. We used the comparison-adjusted funnel plot to explore the potential for publication bias^14^. Additionally, we assessed the certainty of evidence contributing to network estimates of the outcomes with the Grading of Recommendations Assessment, Development and Evaluation (GRADE) framework^15^.

Finally, to validate the robustness of the findings based on the frequentist network meta-analysis, we performed a Bayesian random-effects network meta-analysis^16^ in Winbugs (version 1.4.3). We used Markov chain Monte–Carlo simulation method to calculate odds ratio and 95% CI. Four Markov chains were run simultaneously with 50 000 simulated iterations after a burn-in of 20 000 iterations and a thinning interval of 1. Trace plots and the Brooks-Gelman-Rubin statistic were assessed to ensure convergence. Model fit was evaluated with the total residual deviance and data points. Besides, we performed other two sensitivity analyses by excluding studies not reporting 30-day clinical outcomes and by excluding studies investigating DFM valve.

## Results

### Characteristics of included studies and bias assessment

**Figure 1:**
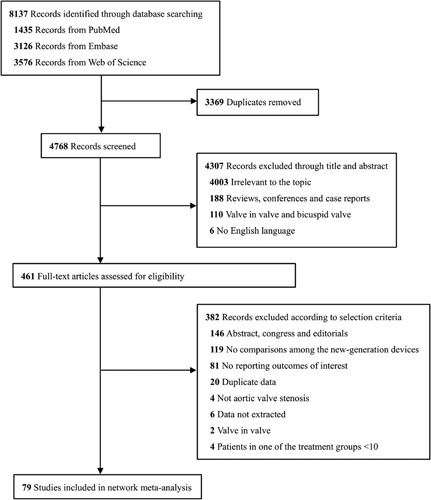
Flow chart of study selection.

Details about the literature search was shown in the Supplementary Table 1, Supplemental Digital Content 4, http://links.lww.com/JS9/A449 and Figure 1. We identified 8137 records from the three databases. After the initial screening of titles and abstracts, 461 full-text articles were considered potentially relevant. Of these, 79 studies^17–95^ including 5 RCTs and 74 observational studies (19 propensity score matched studies) met our inclusion criteria for this network meta-analysis. Of the 79 studies, 60 compared two intervention groups, 8 compared three interventions, 8 compared four interventions and the remaining 3 compared five interventions.

Baseline clinical, imaging and procedural characteristic of the included participants were shown in Supplementary Table 2-3, Supplemental Digital Content 4, http://links.lww.com/JS9/A449. The risk of bias assessment of the included studies was presented in Supplementary Table 4-5, Supplemental Digital Content 4, http://links.lww.com/JS9/A449. Among the observational studies, 52.7% (39/74) studies had moderate risk bias and 47.3% (35/74) studies had low risk of bias. Among the 5 RCTs, only 1 study was assessed to have low risk of bias, and the remaining 4 studies have moderate risk of bias.

## Primary endpoint

### Device success

**Figure 2:**
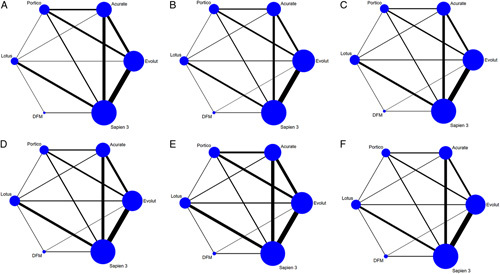
Network plot of new-generation valves for (A) device success; (B) mortality; (C) stroke; (D) major/life-threatening bleeding; (E) major vascular complication; (F) acute kideny injury. The size of the nodes is proportional to the number of participants to each treatment and the thickness of the lines is proportional to the number of studies evaluating each direct comparison. DFM, direct flow medical.

**Figure 3:**
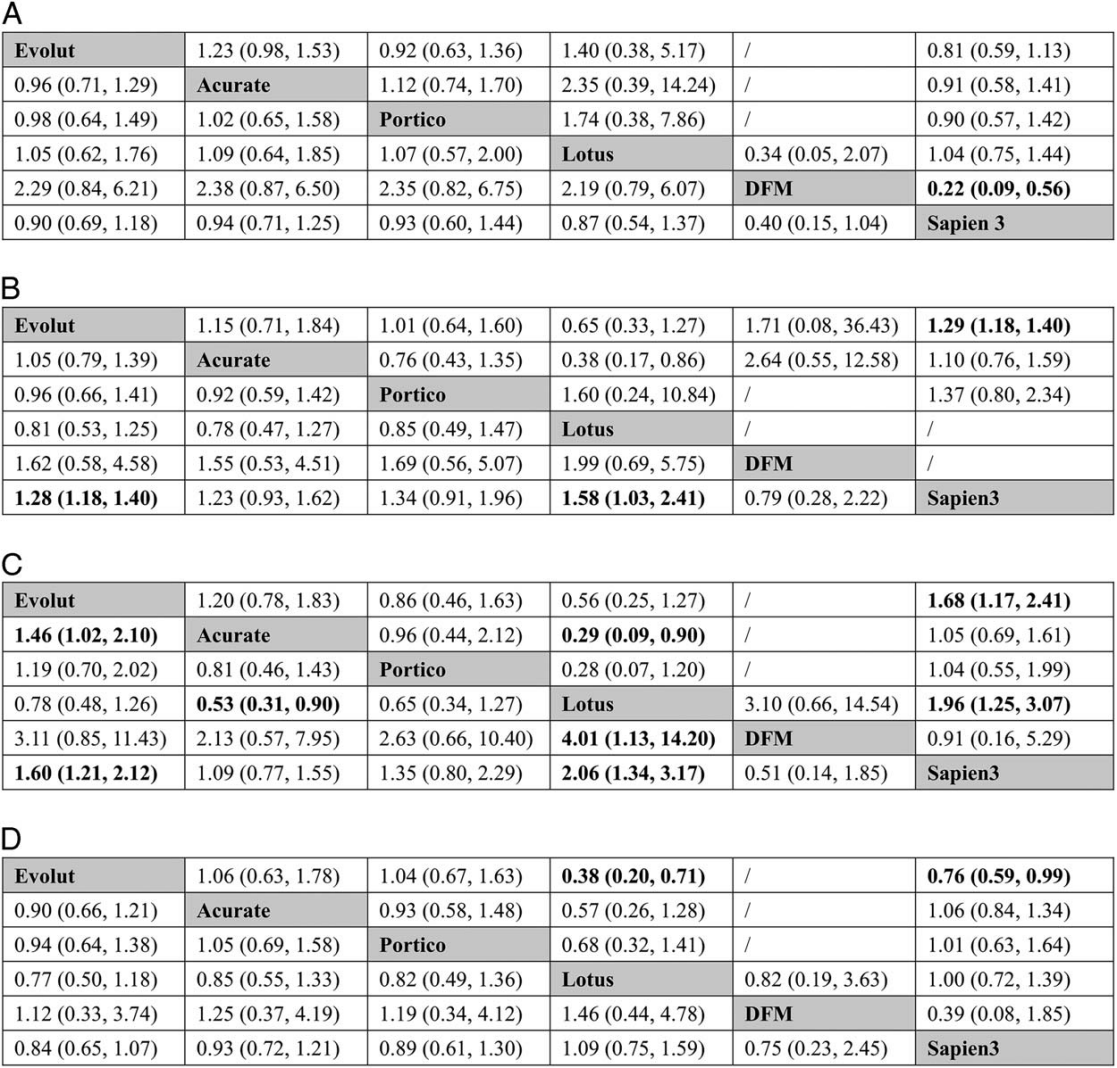
Results of pairwise (upper right portion) and network (lower left portion) meta-analysis are shown for (A) device success; (B) mortality; (C) stroke; (D) major/life-threatening bleeding. Data in each cell are odds ratios (95% credible intervals) for the comparison of row-defining treatment versus column-defining treatment. Significant results are in bold. DFM, direct flow medical.

A total of 43 studies with 17 536 patients contributed to the analysis of device success (Fig. 2 A). The total device success rate was 89.0%. Results of pairwise analysis and network meta-analysis were shown in Figure 3 A. According to the SUCRAs (Supplementary Table 6A, Supplemental Digital Content 4, http://links.lww.com/JS9/A449 and Supplementary Figure 3A, Supplemental Digital Content 4, http://links.lww.com/JS9/A449), the top ranked valve for lower rate of device success was DFM (4.6%). Lotus (48.8%), Evolut (51.8%), Portico (58.3%), Acurate (61.3%) and Sapien 3 (75.3%) had similar probabilities to be the best valve for device success.

## Secondary endpoints

### Mortality

A total of 58 studies with 92 102 patients contributed to the analysis of mortality (8 studies reported in-hospital mortality and 50 studies reported 30-day mortality, Fig. 2 B). The total mortality was 2.7%. Results of pairwise analysis and network meta-analysis were shown in Figure 3 B. According to the SUCRAs (Supplementary Table 6B, Supplemental Digital Content 4, http://links.lww.com/JS9/A449 and Supplementary Figure 3B, Supplemental Digital Content 4, http://links.lww.com/JS9/A449), Sapien 3 (16.8%) and DFM (19.7%) were the top two ranked valves for lower mortality, followed by Acurate (52.0%), Evolut (60.7%), Portico (65.7%) and Lotus (85.1%).

## Stroke

A total of 50 studies with 22 862 patients contributed to the analysis of stroke (13 studies reported in-hospital stroke and 37 studies reported 30-day stroke, Fig. 2 C). The total stroke rate was 2.1%. Results of pairwise analysis and network meta-analysis were shown in Figure 3 C. According to the SUCRAs (Supplementary Table 6C, Supplemental Digital Content 4, http://links.lww.com/JS9/A449 and Supplementary Figure 3C, Supplemental Digital Content 4, http://links.lww.com/JS9/A449), the top ranked valve associated with lower rate of stroke was DFM (8.6%), followed by Sapien 3 (25.5%), Acurate (36.5%), Portico (58.7%), Evolut (76.7%) and Lotus (94.1%).

### Major or life-threatening bleeding

A total of 47 studies with 21 156 patients contributed to the analysis of bleeding (16 studies reported in-hospital bleeding and 31 studies reported 30-day bleeding, Fig. 2 D). The total bleeding rate was 5.0%. Results of pairwise meta-analysis and network meta-analysis were shown in Figure 3 D. According to the SUCRAs (Supplementary Table 6D, Supplemental Digital Content 4, http://links.lww.com/JS9/A449 and Supplementary Figure 3D, Supplemental Digital Content 4, http://links.lww.com/JS9/A449), Evolut (27.6%), DFM (35.8%) and Portico (42.7%) were ranked in the 3 first places for the lower rates of bleeding, followed by Acurate (50.6%), Sapien 3 (67.1%) and Lotus (76.3%).

### Major vascular complication

**Figure 4:**
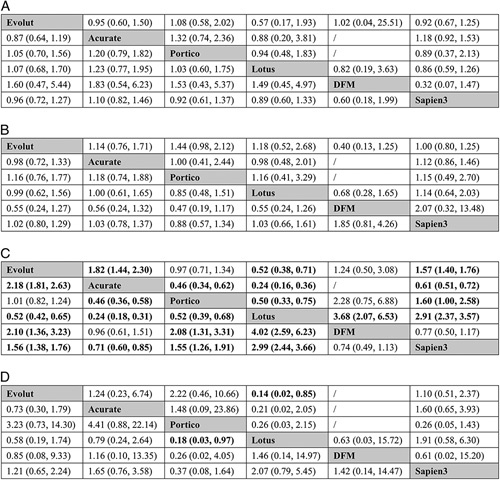
Results of pairwise (upper right portion) and network (lower left portion) meta-analysis are shown for (A) major vascular complication; (B) acute kideny injury; (C) permanent pacemaker implantation; (D) procedural mortality. Data in each cell are odds ratios (95% credible intervals) for the comparison of row-defining treatment versus column-defining treatment. Significant results are in bold. DFM, direct flow medical.

A total of 44 studies with 18 835 patients contributed to the analysis of MVC (18 studies reported in-hospital MVC and 26 studies reported 30-day MVC, Fig. 2 E). The total rate of MVC was 5.9%. No significant differences among the six devices were found in either pairwise meta-analysis or network meta-analysis (Fig. 4 A). According to the SUCRAs (Supplementary Table 6E, Supplemental Digital Content 4, http://links.lww.com/JS9/A449 and Supplementary Figure 3E, Supplemental Digital Content 4, http://links.lww.com/JS9/A449), DFM (22.4%) was the top ranked valve for lower rate of MVC, followed by Lotus (40.8%), Portico (44.8%), Evolut (51.3%) and Sapien 3 (61.1%) and Acurate (79.6%).

### Acute kidney injury

A total of 38 studies with 15681 patients contributed to the analysis of AKI (12 studies reported in-hospital AKI and 26 studies reported 30-day AKI, Fig. 2 F). The total AKI rate was 4.5%. No significant differences among the six valves were found in either pairwise meta-analysis or network meta-analysis (Fig. 4 B). According to the SUCRAs (Supplementary Table 6F, Supplemental Digital Content 4, http://links.lww.com/JS9/A449 and Supplementary Figure 3F, Supplemental Digital Content 4, http://links.lww.com/JS9/A449), Portico (22.6%) was the top ranked valve for lower rate of AKI, followed by Sapien 3 (41.9%), Evolut (46.5%), Lotus (47.0%), Acurate (50.1%) and DFM (92.1%).

### Permanent pacemaker implantation

**Figure 5:**
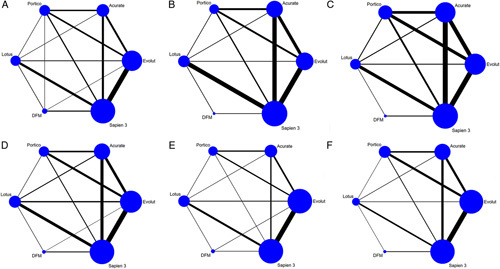
Network plot of new-generation valves for (A) permanent pacemaker implantation; (B) procedural mortality; (C) no correct position; (D) moderate-to-severe paravalvular leak; (E) prosthesis patient mismatch; (F) mean aortic valve gradients. The size of the nodes is proportional to the number of participants to each treatment and the thickness of the lines is proportional to the number of studies evaluating each direct comparison. DFM, direct flow medical.

A total of 68 studies with 94 115 patients contributed to the analysis of PPI (24 studies reported in-hospital PPI and 44 studies reported 30-day PPI, Fig. 5 A). The total PPI rate was 19.3%. Results of pairwise meta-analysis and network meta-analysis were shown in Figure 4 C. According to the SUCRAs (Supplementary Table 6G, Supplemental Digital Content 4, http://links.lww.com/JS9/A449 and Supplementary Figure 3G, Supplemental Digital Content 4, http://links.lww.com/JS9/A449), Acurate (8.6%), DFM (13.2%) and Sapien 3 (38.3%) were ranked in the 3 first places with respect to lower rates of PPI, followed by Portico (69.6%), Evolut (70.3%) and Lotus (100.0%).

## Other endpoints

### Procedural mortality

A total of 24 studies with 10 195 patients contributed to the analysis of procedural mortality (Fig. 5 B). The total procedural mortality was 0.78%. Results of pairwise meta-analysis and network meta-analysis were shown in Figure 4 D. According to the SUCRAs (Supplementary Table 6H, Supplemental Digital Content 4, http://links.lww.com/JS9/A449 and Supplementary Figure 3H, Supplemental Digital Content 4, http://links.lww.com/JS9/A449), Portico (7.7%), Sapien 3 (34.5%) and Evolut (50.4%) were the top three ranked valves for lower procedural mortality, followed by DFM (57.0%), Acurate (70.0%) and Lotus (80.3%).

### No correct positioning (NCP)

**Figure 6:**
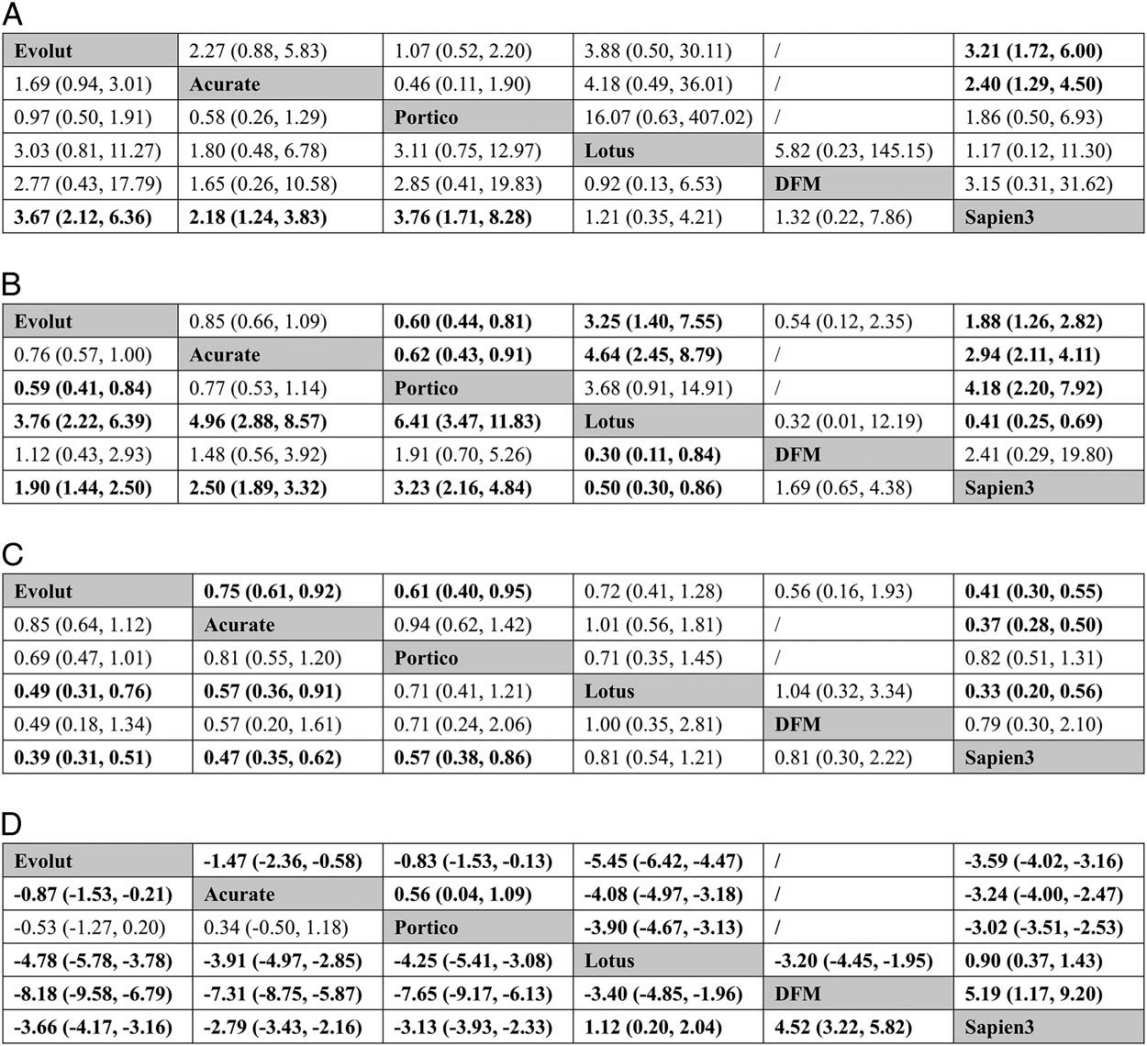
Results of pairwise (upper right portion) and network (lower left portion) meta-analysis are shown for (A) no correct position; (B) moderate-to-severe paravalvular leak; (C) prosthesis patient mismatch; (D) mean aortic valve gradients. Data in each cell are odds ratios (95% credible intervals) for the comparison of row-defining treatment versus column-defining treatment. Significant results are in bold. DFM, direct flow medical.

A total of 35 studies with 14 571 patients contributed to the analysis of no correct positioning (Fig. 5 C). The total rate of no correct positioning was 1.0%. Results of pairwise meta-analysis and network meta-analysis were shown in Figure 6 A. According to the SUCRAs (Supplementary Table 6I, Supplemental Digital Content 4, http://links.lww.com/JS9/A449 and Supplementary Figure 3I, Supplemental Digital Content 4, http://links.lww.com/JS9/A449), Sapien 3 (15.3%), Lotus (27.7%) and DFM (34.9%) were the top three valves associated with lower rates of no correct positioning, followed by Acurate (52.6%), Evolut (84.6%) and Portico (85.0%).

### Moderate-to-severe paravalvular leak

A total of 53 studies with 21 990 patients contributed to the analysis of PVL ( **Fig. 5 D** ). The total rate of PVL was 4.2%. Pairwise meta-analysis and network meta-analysis found similar results and results were shown in Figure 6 B. According to the SUCRAs (Supplementary Table 6J, Supplemental Digital Content 4, http://links.lww.com/JS9/A449 and Supplementary Figure 3J, Supplemental Digital Content 4, http://links.lww.com/JS9/A449), Lotus (0.3%) and Sapien 3 (22.7%) were the top two ranked valves associated with lower rates of moderate to severe PVL, followed by DFM (51.7%), Evolut (52.2%), Acurate (76.9%) and Portico (96.2%).

### Prosthesis patient mismatch

A total of 20 studies with 7914 patients contributed to the analysis of PPM (Fig. 5 E). The total PPM rate was 24.9%. Results of pairwise meta-analysis and network meta-analysis were shown in Figure 6 C. According to the SUCRAs (Supplementary Table 6K, Supplemental Digital Content 4, http://links.lww.com/JS9/A449 and Supplementary Figure 3K, Supplemental Digital Content 4, http://links.lww.com/JS9/A449), Evolut (4.7%) and Acurate (23.5%) were ranked in the top two places for lower rates of PPM, followed by Portico (43.8%), DFM (67.0%), Lotus (70.6%) and Sapien 3 (90.3%).

### Mean aortic valve gradients

A total of 41 studies with 15 578 patients contributed to the analysis of MAVG (Fig. 5 F). Pairwise meta-analysis and network meta-analysis found similar results and results were shown in Figure 6 D. According to the SUCRAs (Supplementary 6L, Supplemental Digital Content 4, http://links.lww.com/JS9/A449 and Supplementary Figure 3L, Supplemental Digital Content 4, http://links.lww.com/JS9/A449), Evolut (1.5%), Portico (23.0%) and Acurate (35.5%) were the top three valves for lower MAVG, followed by Sapien 3 (60.2%), Lotus (79.8%) and DFM (100.0%).

### Heterogeneity, inconsistency and transitivity

Network heterogeneity was low to moderate in all endpoints except for MAVG (Supplementary Table 7, Supplemental Digital Content 4, http://links.lww.com/JS9/A449). The test of global inconsistency by using the design­by­treatment interaction models showed no significant inconsistency for any outcomes except for bleeding, PPI and PPM (Supplementary Table 8, Supplemental Digital Content 4, http://links.lww.com/JS9/A449). Loop-specific approach identified small percentage of loops presenting statistical inconsistency for outcomes (Supplementary Table 9, Supplemental Digital Content 4, http://links.lww.com/JS9/A449). Besides, the test from node-splitting model showed significant differences between direct and indirect comparisons in some outcomes (Supplementary Table 10, Supplemental Digital Content 4, http://links.lww.com/JS9/A449). The distribution of potential effect modifiers was balanced across most of the treatment comparisons (Supplementary Figure 1, Supplemental Digital Content 4, http://links.lww.com/JS9/A449).

### Small-study effects and Quality of evidence

The comparison-adjusted funnel plots suggested no potential publication bias or small-study effect for all outcomes (Supplementary Figure 2, Supplemental Digital Content 4, http://links.lww.com/JS9/A449). We downgraded evidence certainty to low or very low for most comparisons of primary, secondary and other outcomes, mainly because of study limitations, imprecision and inconsistency (Supplementary Table 11-12, Supplemental Digital Content 4, http://links.lww.com/JS9/A449 and Supplementary Figures 4-6, Supplemental Digital Content 4, http://links.lww.com/JS9/A449).

### Sensitive analyses

Sensitive analyses by using a Bayesian framework random-effects model, which had a good model fit, showed that effect estimates and SUCRA values for each endpoint did not change substantially compared with the frequentist network meta-analyses. Sensitive analyses by excluding studies not reporting 30-day outcomes showed that DFM obtained lower ranks for bleeding and MVC, but no substantial changes in study effect estimates and SUCRA values were found for other endpoints. Additionally, removing DFM from the network meta-analysis had little impact on the effect estimates and SUCRA values of other treatments (Supplementary Tables 13-15, Supplemental Digital Content 4, http://links.lww.com/JS9/A449).

## Discussion

To the best of our knowledge, this was the first network meta-analysis to compare these six different new-generation valves for AS and it demonstrated that the device success rates were comparable among these newer valves except for DFM. After excluding DFM, Sapien 3 appeared to be the best effective for reducing mortality and stroke; Lotus present to be the best effective for decreased PVL; Evolut appeared to be the best effective for decreased MLTB and MAVG; Acurate and Portico might be the best effective for decreased PPI and AKI, respectively.

### Device success and hemodynamics performance

Our study showed comparable rates of device success among Sapien 3, Lotus and self-expandable valves (SEVs), which was consistent with the findings of hemodynamics performance in these valves. New-generation TAVI devices all incorporate a dedicated design feature to reduce the risk of PVL. The adaptive seal technology and the full repositionability for optimal positioning prior to final deployment could significantly reduce the incidence of PVL in the Lotus valve^3,4,96,97^. And the lower rate of PVL showed by Sapien 3 might be related to its double outer and inner skirt in the inflow portion of the frame and to the higher radial force that enhances the sealing of the valve into the native annulus^3,4,98^. However, despite the present of an outer skirt, the risk of PVL was higher in the SEVs, especially in Acurate and Portico. This could be explained by the lower radial force which may translate into malappositioning of the device to the aortic annulus^3–5^. This assumption was consistent with our findings that the rate of NCP was higher in SEVs than in Lotus and Sapien 3.

In contrast, SEVs showed lower transvalvular gradients compared with Lotus and Sapien 3. The intra-annular leaflet position of Sapien 3 and infra-annular leaflet position of Lotus might increase the additional constraints by native annulus combined to leaflets and stent, resulting in higher transvalvular gradients, whereas supra-annular position of the leaflets in the self-expanding device allowing lower resistance to the left ventricle outflow (LVOT) seems to be beneficial to achieve lower gradients^94,99^. Interestingly, we observed that Portico, which is the only one with intra-annular leaflets among currently available SEVs, showed a significantly higher gradient compared with the Evolut but showed a lower gradient than Acurate, suggesting that supra-annular design was a crucial but not the only one factor for improved hemodynamics performance. A possible explanation of the favourable hemodynamic of the Portico valve may be related to the tubular shape and the large cell area at the annulus section of the stent frame, which might allow a wider implantation range and reduce the tissue interference, resulting in physiological flow and low transvalvular gradients^3–5,41,54,100^. Future studies are required to evaluate how this intra-annular system compares with other TAVI systems.

In addition, the present study showed lower frequency of PPM in SEVs than in Lotus and Sapien 3. However, the results should be interpreted with caution because the severity of PPM, which was assessed by transthoracic echocardiogram derived indexed effective orifice area (EOA), was more often overestimated in Sapien 3 compared with SEVs due to the pressure recovery phenomenon^101,102^. Fukui et al. demonstrated that using computed tomography-derived EOA instead of transthoracic echocardiogram derived EOA changed the frequency of PPM observed with SEVs and Sapien 3 from 11.1% versus 24.6% (*P* = 0.002) to 2.3% versus 4.2% (*P* = 0.399)^35^. Further studies using computed tomography-derived EOA to assess PPM in new-generation devices are required.

The DFM system was designed with non-metallic, inflatable and deflatable structure, allowing precise positioning, retrieval and assessment of the valve performance in its final position^103^. Although this product has been withdrawn from the market in late 2016 because it failed to achieve a sustainable breakthrough^104^, we enroled this newer device in our network analysis to evaluate the concept of non-metallic design. The present study showed DFM had the highest risk of device failure but had promising performance in clinical outcomes. These results should be interpreted cautiously because of the limited studies contributing to the analyses of DFM for each endpoint. Further researches on improvement of DFM are worthy of expectation.

### Mortality

The present study showed that the all-cause mortality was 2.7% in the new-generation TAVI devices, achieving a marked reduction compared with that in first-generation TAVI devices reported by previous meta-analysis^105^. Besides, we observed that Sapien 3 had the lowest risk of mortality among the new-generation devices. This finding could be partially explained by the favourable performance of Sapien 3 in reducing the incidence of stroke, AKI and PPI, which were found to be strong predictors for short-term mortality. In contrast, Lotus had the highest risk of mortality due to its poor performance in those clinical outcomes mentioned above.

### Stroke

Our study has demonstrated that Lotus and Sapien 3 had the highest and lowest stroke risk among the newer devices, respectively. Previous studies suggested that most of the TAVI related strokes were embolic and developed mainly intraprocedural^106^. One possible explanation for an increased stroke risk with the Lotus valve could be that repositioning of the retrievable valve may increase the frequency of friction between the valve prosthesis and landing zone, which, in turn, may increase the risk of plaque abrasion, causing embolization and subsequent cerebrovascular events^96,97^. Likewise, the higher stroke risk in SEVs could be explained by the higher rate of balloon postdilation required in these groups, which could increase the mechanical interaction between the stent frame of the valve prosthesis and the native aortic valve. Besides, SEVs systems are larger and implanted more slowly and stepwise accordingly more extensive manipulation and longer implantation time are required in SEVs, which might also increase risk of embolization^93^. Compared with Lotus and SEVs, the Sapien 3 delivery systems are more steerable and had lower manipulation with rather rapid positioning, reducing the fragments originating from aortic valve, aortic arch and left ventricular myocardium^93^. Cerebral emboli protection (CEP) systems might be an alternative to further reduce periprocedural ischaemic events. However, current data do not support routinely use of CEP during TAVI due to the controversial results^107–109^. Further clinical trials investigating the efficacy of CEP use are expected.

### Major vascular complication

The insertion of larger sheaths in the common femoral artery strongly correlates with higher MVC rates. New-generation TAVI systems are designed to decrease vascular trauma, by introducing smaller sheath profiles, including expandable (e.g. e-shealth, iSLEEVE), balloon-expandable (e.g. SoloPath)^110^. However, there was no evidence yet on the actual clinical benefit of expandable sheaths over fixed sheaths^110–112^. Several hypothetical explanations could be proposed for the failure of the expandable sheaths to diminish the frequency of MVC. First, although the initial smaller diameter of expandable sheaths, the sheaths will have a diameter similar to the fixed sheath during the expanded phase, and trauma exerted on the vascular structures will be arguably similar. Second, during the post-expanded phase, the sheaths will be deformed and will lack the initial smooth surface and size. Complete recoil to the initial smaller calibre could lead to a situation that the arterial cut is larger than the introducer, with the risk of bleeding around the sheath^111–115^.

The integrated delivery sheaths represent a different solution for reducing sheath size^110^. This In-Line sheath concept allows a true low profile of a 14–16 French equivalent, resulting in a lower sheath-to-femoral-artery-ratio. However, utilization of the integrated delivery sheaths required repeated vessel entries with large bore sheaths to perform pre-dilatation and post-dilatation and THV implantation, respectively, which might counterbalance the benefits of lower sheath-to-femoral-artery-ratio^113,114^.

Consistently, our study found that neither Sapien 3 and Acurate using expandable sheaths nor Evolut using integrate sheaths presented significant advantages on reducing MVC rates compared with those using fixed sheaths. Improvements on the design of the valve prosthesis and the delivery system rather than on sheaths itself might be more worthwhile.

### Major or life-threatening bleeding

In our study, the risk of MLTB was higher in Lotus and Sapien 3 valve compared with SEVs. Several reasons might contribute to these findings. First, the lack of benefit in vascular complications might partially accounts for the absence of a reduction in bleeding complications with the Sapien 3. Secondly, previous studies found that the use of BEV was associated with a higher risk of thrombocytopenia compared with SEVs, which was associated with an increased risk of MLTB^116,117^. Several mechanisms have been proposed to explain the thrombocytopenia following TAVI, including drug toxicity related to the valve preparation or material fixation, mechanical platelet destruction, increased platelet consumption, decreased platelet production and activation of the coagulation cascade ^118,119^.

We hypothesized that the Lotus valve might also have high risk of mechanical platelet destruction due to the repositioning of the valve and high risk of thrombocytopenia due to platelet and coagulation hyperactivity as a result of exposure to the high density of stent struts of the valve. Besides, before being loaded on to the delivery system, for valve preparation, the bovine pericardial biomaterial used in Sapien 3 and Lotus prostheses should undergo additional anticalcification treatment with homocysteic acid, which has been suggested to trigger chemically induced plateletlysis^118,119^.

### Permanent pacemaker implantation

Our study has demonstrated that despite the use of new-generation TAVI devices, the total PPI rate remains high with 19.3% in TAVI patients. Louts and Acurate had the highest risk and the lowest risk of PPI, respectively. Several aspects may contribute to higher risk of PPI in Lotus. First, the device has a very high radial strength and a high density of metal in its frame, which may impose pressure on the conduction system embedded in the interventricular septum adjacent to the aortic annulus. Second, Lotus frame remains in contact with the wall of the LVOT throughout the process of foreshortening and locking, which could be more harmful to the conduction system^96,97^. Unlike other SEVs, Acurate is initially released from the aorta rather than from the LVOT, with subsequent deployment of the sub-annular portion. This top-down deployment mechanism, with little interference with the LVOT during implantation and a lower radial force on the conduction system, may account for the lower PPI rates with this THV^120,121^. Additionally, we observed that Sapien 3 had relatively lower risk of PPI compared with SEVs, which could hypothetically be caused by the shorter metal of Sapien 3 and lower requirement for post-dilatation in this prosthesis^98^.

### Acute kidney injury

Our study found that the total AKI rate in the new-generation device was 4.5% with no significant differences between the valves. Rapid ventricular pacing during the TAVI procedure resulting in significant hypotension has been demonstrated to be associated with the AKI^122,123^. However, in the present study Acurate showed no significant advantages than other devices despite its ability of valve positioning without rapid ventricular stimulation. Possible explanation was that rapid pacing was also required during the procedure of pre-dilatation which was performed more frequently in the Acurate group. Additionally, previous data demonstrated that the presence of post-procedural PVL was associated with high risk of AKI^122,123^. However, in the present study we did not observe a higher risk of AKI in Portico despite it had the highest risk of PVL among the newer devices. Larger systematic studies investigating the association of PVL with the occurrence of AKI in new-generation devices are required.

## Limitations

Our study has some limitations. First, most of the studies included in this network meta-analysis were observational studies, confounding factors were inherent and the risk estimates may be underestimated. Besides, the observational study limited the level of evidence of this study to a low or very low level. More high-quality RCTs are required to update this network meta-analysis to get more reliable results. Second, the differences of aortic valve area, annulus diameter, annulus perimeter, annulus area, aortic angle and the extent of aortic valve calcification among individual patients and surgeon experience might explain the moderate amounts of heterogeneity in some of our analyses such as PPI, MVC, MLTB and PPM. However, due to the limited data, we could not perform subgroup analysis according to these variables which might modify treatment benefits. Third, we only discussed the device related factors contributing to the different findings in our study. However, indeed, patient-related factors and operators learning curve are also potential contributors for the results. Fourth, this study only investigated the short-term clinical outcomes of the new-generation devices, whether the findings of the present study were maintained during long-term follow-up remains unclear.

## Conclusions

The findings of the present study suggested that the device success rates were comparable among these new-generation valves except for DFM. After excluding DFM, Sapien 3 might be the best effective for decreased mortality and stroke; Lotus might be the best effective for decreased PVL; Evolut might be the best effective for decreased MLTB and MAVG; Acurate and Portico might be the best effective for decreased PPI and AKI, respectively. The non-metallic design of DFM system was challenging but worthy of further exploration.

## Ethical approval

Ethical approval was given in every original study.

## Source of funding

This study was supported by Ministry of Science and Technology of the People's Republic of China (2016YFC1301102) and Clinical Incubation Program of Beijing Chaoyang Hospital (CYFH202204).

## Author contribution

L.-F.W. and P.-X.S. conceived and designed the study; Y.-X.Y. and X.-M.L. wrote the first draft of the manuscript and they contributed equally to this work; Y.F. and C.L. selected studies; H.-J.W. and L.X. conducted the data analysis and interpretation; Z.-Y.Z. and K.X. conducted graphic design; M.-L.C. and J.-C.Z. revised the manuscript. All authors had full access to all the data in the study and accept responsibility to submit for publication.

## Conflicts of interest disclosure

The authors declare that they have no competing interests.

## Research registration unique identifying number (UIN)

1. Name of the registry: Prospero.
2. Unique Identifying number or registration ID: CRD42021224646.
3. Hyperlink to your specific registration (must be publicly accessible and will be checked): https://www.crd.york.ac.uk/prospero/display_record.php?ID=CRD42021224646.

## Guarantor

Le-Feng Wang.

## Data statement

We declare that all data are available from previously published articles.

## Provenance and peer review

Not commissioned, externally peer-reviewed.

## Supplementary Material
