# Supplementary material for: Comparisons of different new-generation transcatheter aortic valve implantation devices for patients with severe aortic stenosis: a systematic review and network meta-analysis
Source: Int J Surg. 2023 May 10;109(8):2414–26. doi: 10.1097/JS9.0000000000000456 (PMC10442113; doi:10.1097/JS9.0000000000000456)
Supplement: Supplementary file 1 [file js9-109-2414-s001.docx]

**Identification of studies via databases and registers**

Records removed before screening: Duplicate records removed (n = 3369)

Records identified from PubMed, Embase and Web of Science

Databases (n = 8137)

**Identification**

Records excluded

(n = 4307)

4003 Irrelevant to the topic

188 Reviews, conferences and case reports

110 Valve in valve and bicuspid valve

6 No English language

Records screened

(n = 4768)

**Screening**

Reports excluded:382

146 Abstract, congress and editorials

119 No comparisons among the new-generation devices

81 No reporting outcomes of interest

20 Duplicate data

4 Not aortic valve stenosis

6 Data not extracted

2 Valve in valve

4 Sample size < 10

Reports assessed for eligibility

(n = 461)

Reports of included studies

(n = 79)

**Included**

*Consider, if feasible to do so, reporting the number of records identified from each database or register searched (rather than the total number across all databases/registers).

**If automation tools were used, indicate how many records were excluded by a human and how many were excluded by automation tools.

*From:*  Page MJ, McKenzie JE, Bossuyt PM, Boutron I, Hoffmann TC, Mulrow CD, et al. The PRISMA 2020 statement: an updated guideline for reporting systematic reviews. BMJ 2021;372:n71. doi: 10.1136/bmj.n71

For more information, visit: <http://www.prisma-statement.org/>
