# Supplementary material for: Comparisons of different new-generation transcatheter aortic valve implantation devices for patients with severe aortic stenosis: a systematic review and network meta-analysis
Source: Int J Surg. 2023 May 10;109(8):2414–26. doi: 10.1097/JS9.0000000000000456 (PMC10442113; doi:10.1097/JS9.0000000000000456)
Supplement: Supplementary file 4 [file js9-109-2414-s004.docx]

**Contents**

**1. Table 1.** Details about the systematic literature search

**2. Table 2.** Baseline clinical characteristic of the included studies

**3. Table 3.** Baseline imaging and procedural characteristics of the included studies

**4. Table 4.** Quality assessment of observational studies

**5. Table 5.** Quality assessment of randomized control trials (RCTs)

**6. Table 6.** Surface under the cumulative rankings (SUCRAs) and the mean ranks for each endpoint

**7. Table 7.** Network heterogeneity for each endpoint

**8. Table 8.** Design-by-treatment test for each endpoint

**9. Table 9.** Loop-specific heterogeneity estimates for each endpoint

**10. Table 10.** Network side-split all for each endpoint

**11. Table 11.** Summary of GRADE domain and procedure for pairwise effect estimate and network estimate

**12. Table 12.** Summary of our confidence in effect estimates and ranking of treatments for each endpoint

**13. Table 13.** Sensitivity analyses of effect estimates for each endpoint

**14. Table 14.** Sensitivity analyses of SUCRA values and mean rank for each endpoint

**15. Table 15.** Assessment of Bayesian random effects model fit

**16. Figure 1.** Assessment of the transitivity assumption

**17. Figure 2.** Comparison-adjusted funnel plot for each endpoint

**18. Figure 3.** Cumulative ranking plots for each endpoint

**19. Figure 4.** Contribution matrix for each endpoint

**20. Figure 5.** 95% Prediction interval for each endpoint

**21. Figure 6.** Summary of study limitations for each endpoint

**Table 1. Details about the systematic** **literature search**

| **Databases** | **Search terms** | **Numbers of literatures** |
| --- | --- | --- |
| PubMed | #1 (aortic valve stenosis(MeSH Terms]) | 43,453 |
|  | #2 (aortic stenosis(Text Word]) OR (aortic valve(Text Word]) | 71,466 |
|  | #3 #1 OR #2 | 87,871 |
|  | #4 ((((((evolut(Text Word]) OR (acurate(Text Word])) OR (portico(Text Word])) OR (sapien 3(Text Word])) OR (lotus valve(Text Word])) OR (direct flow medical valve(Text Word])) OR (DFM valve(Text Word]) | 1,072 |
|  | #5 ((((((((((((((((((((self expandable valve*(Text Word]) OR (self expanding valve*(Text Word])) OR (self expanded valve*(Text Word])) OR (balloon expandable valve*(Text Word])) OR (balloon expanding valve*(Text Word])) OR (balloon expanded valve*(Text Word])) OR (mechanically expandable valve(Text Word])) OR (mechanically expanding valve(Text Word])) OR (mechanically expanded valve(Text Word])) OR (new generation valve*(Text Word])) OR (newer generation valve*(Text Word])) OR (next generation valve*(Text Word])) OR (latest generation valve*(Text Word])) OR (second generation valve*(Text Word])) OR (third generation valve*(Text Word])) OR (new generation device*(Text Word])) OR (newer generation device*(Text Word])) OR (next generation device*(Text Word])) OR (latest generation device*(Text Word])) OR (second generation device*(Text Word])) OR (third generation device*(Text Word]) | 1,119 |
|  | #6 #4 OR #5 | 2,047 |
|  | #7 #3 AND #6 | 1,435 |
| Embase | #1 'aortic valve stenosis'/exp | 19,558 |
|  | #2 'aortic stenosis':ab,ti OR 'aortic valve':ab,ti | 87,572 |
|  | #3 #1 OR #2 | 92,542 |
|  | #4 evolut:ab,ti OR acurate:ab,ti OR portico:ab,ti OR 'sapien 3':ab,ti OR 'lotus valve':ab,ti OR 'direct flow medical valve':ab,ti OR 'dfm valve':ab,ti | 2,622 |
|  | #5 'self expandable valve*':ab,ti OR 'self expanding valve*':ab,ti OR 'self expanded valve*':ab,ti OR 'balloon expandable valve*':ab,ti OR 'balloon expanding valve*':ab,ti OR 'balloon expanded valve*':ab,ti OR 'mechanically expandable valve':ab,ti OR 'mechanically expanding valve':ab,ti OR 'mechanically expanded valve':ab,ti OR 'new generation valve*':ab,ti OR 'newer generation valve*':ab,ti OR 'next generation valve*':ab,ti OR 'latest generation valve*':ab,ti OR 'second generation valve*':ab,ti OR 'third generation valve*':ab,ti OR 'new generation device*':ab,ti OR 'newer generation device*':ab,ti OR 'next generation device*':ab,ti OR 'latest generation device*':ab,ti OR 'second generation device*':ab,ti OR 'third generation device*':ab,ti | 2,239 |
|  | #6 #4 OR #5 | 4,498 |
|  | #7 #3 AND #6 | 3,126 |
| Web of Science | #1 TS=(aortic valve stenosis) OR TS=(aortic stenosis) OR TS=(aortic valve) | 62,939 |
|  | #2 TS=(evolut) OR TS=(acurate) OR TS=(portico) OR TS=(sapien 3) OR TS=(lotus valve) OR TS=(direct flow medical valve) OR TS=(DFM valve) OR TS=(self expandable valve*) OR TS=(self expanding valve*) OR TS=(self expanded valve*) OR TS=(balloon expandable valve*) OR TS=(balloon expanding valve*) OR TS=(balloon expanded valve*) OR TS=(mechanically expandable valve) OR TS=(mechanically expanding valve) OR TS=(mechanically expanded valve) OR TS=(new generation valve*) OR TS=(newer generation valve*) OR TS=(next generation valve*) OR TS=(latest generation valve*) OR TS=(second generation valve*) OR TS= (third generation valve*) | 5,358 |
|  | #3 #1 AND #2 | 3,576 |
| Total databases |  | 8,137 |
| All duplications |  | 3,369 |
| After de-duplication |  | 4,768 |

**Table 2. Baseline clinical characteristic of the** **included studies**

| **Study** | **Design** | **Treatment** | **PN** | **Age** | **Male** | **HT** | **DM** | **HLP** | **CAD** | **PVD** | **AF** | **COPD** | **STS score** | **EuroSCORE II** | **Logistic EuroSCORE** |
| --- | --- | --- | --- | --- | --- | --- | --- | --- | --- | --- | --- | --- | --- | --- | --- |
| Ben-shoshan 2016 | OB | Evolut R | 108 | 82.7 ± 5.8 | 36 (33.0) | 96 (89.0) | 43 (40.0) | 83 (77.0) | 58 (54.0) | NA | 38 (35.0) | 12 (11.0) | 4.3 ± 2.7 | 5.4 ± 4.1 | NA |
|  |  | Sapien 3 | 124 | 82.0 ± 6.3 | 71 (57.0) | 107 (86.0) | 49 (40.0) | 94 (76.0) | 72 (58.0) | NA | 36 (29.0) | 11 (9.0) | 4.1 ± 4.9 | 5.2 ± 5.7 | NA |
| Enríquez-Rodríguez 2017 | OB (PM) | Evolut R | 64 | 84.0 ± 5.0 | 27 (42.0) | 52 (81.0) | 22 (34.0) | 30 (47.0) | NA | 3 (5.0) | 19 (29.7) | 10 (16.0) | 5.8 ± 5.0 | NA | NA |
|  |  | Sapien 3 | 80 | 82.0 ± 6.0 | 42 (53.0) | 63 (79.0) | 31 (39.0) | 36 (45.0) | NA | 7 (9.0) | 30 (37.5) | 11 (14.0) | 6.2 ± 5.0 | NA | NA |
| Rogers 2017 | OB | Evolut R | 74 | 82.0 ± 8.0 | 29 (39.2) | 65 (88.1) | 22 (30.3) | 62 (83.3) | 43 (58.3) | 16 (21.3) | 26 (35.8) | 29 (39.4) | 8.1 ± 4.6 | NA | NA |
|  |  | Sapien 3 | 183 | 81.0 ± 9.0 | 98 (53.6) | 160 (87.1) | 61 (33.5) | 160 (87.0) | 112 (61.1) | 35 (19.0) | 72 (39.3) | 63 (34.2) | 6.5 ± 6.3 | NA | NA |
| Vlastra 2018 | OB (PM) | Evolut | 1091 | NA | NA | NA | NA | NA | NA | NA | NA | NA | NA | NA | NA |
|  |  | Sapien 3 | 1122 | NA | NA | NA | NA | NA | NA | NA | NA | NA | NA | NA | NA |
| Veulemans 2018 | OB | Evolut R | 101 | 81.4 ± 5.8 | 24 (23.8) | 90 (89.1) | 29 (28.7) | NA | 66 (65.3) | 29 (28.7) | 39 (38.6) | 42 (41.6) | 7.8 ± 5.5 | 10.2 ± 10.1 | 32.2 ± 18.8 |
|  |  | Sapien 3 | 103 | 80.0 ± 7.1 | 59 (57.3) | 94 (91.3) | 33 (32.0) | NA | 79 (76.7) | 20 (19.4) | 33 (32.0) | 30 (29.1) | 6.4 ± 5.4 | 9.1 ± 9.2 | 26.8 ± 17.5 |
| Finkelstein2018 | OB (PM) | Evolut R | 126 | 82.0 (76.0-86.0) | 78 (61.9) | 103 (81.7) | 45 (35.7) | 80 (63.5) | 74 (58.7) | 15 (11.8) | 43 (34.4) | 21 (16.7) | 3.2 (2.2-4.7) | NA | NA |
|  |  | Sapien 3 | 126 | 82.0 (78.0-85.0) | 84 (66.7) | 106 (84.1) | 51 (40.5) | 86 (68.3) | 78 (61.9) | 13 (10.4) | 35 (27.8) | 19 (15.1) | 3.2 (2.3-4.8) | NA | NA |
| Abdelghani 2018 | OB | Evolut R | 100 | 81.5 ± 4.9 | 37 (37.0) | 91 (91.0) | 25 (25.0) | NA | 56 (56.0) | 24 (24.0) | 45 (45.0) | NA | 4.3 ± 3.9 | NA | NA |
|  |  | Sapien 3 | 334 | 80.8 ± 6.3 | 184 (55.1) | 293 (87.7) | 97 (29.0) | NA | 225 (67.4) | 56 (16.8) | 131 (39.2) | NA | 5.0 ± 3.8 | NA | NA |
| Eitan 2018 | OB | Evolut R | 37 | 82.4 ± 5.8 | 34 (91.9) | 35 (94.6) | 12 (32.4) | 29 (78.4) | 29 (78.4) | 13 (32.4) | 20 (54.1) | 9 (24.3) | 4.6 ± 2.4 | 5.7 ± 3.8 | 25.7 ± 14.3 |
|  |  | Sapien 3 | 55 | 80.9 ± 6.3 | 52 (94.5) | 45 (81.8) | 14 (25.5) | 31 (56.4) | 38 (69.1) | 12 (22.2) | 23 (41.8) | 11 (20.0) | 3.9 ± 2.5 | 4.4 ± 3.6 | 17.9 ± 10.7 |
| Akodad 2018 | OB | Evolut R | 81 | 83.8 ± 6.3 ^#^ | 97 (42.5) ^#^ | 155 (68.0) ^#^ | 65 (28.5) ^#^ | 63 (27.6) ^#^ | 102 (44.7) ^#^ | 28 (12.3) ^#^ | NA | NA | NA | 4.1 ± 2.8 ^#^ | 15.0 ± 9.6 ^#^ |
|  |  | Sapien 3 | 147 |  |  |  |  |  |  |  | NA | NA | NA |  |  |
| Mosleh 2019 | OB | Evolut R/Pro | 129 | 82.0 ± 7.8 | 67 (52.0) | 117 (91.0) | 41 (32.0) | NA | NA | NA | 102 (79.0) | NA | 13.0 ± 9.1 | NA | NA |
|  |  | Sapien 3 | 452 | 81.3 ± 8.2 | 252 (56.0) | 409 (90.0) | 139 (31.0) | NA | NA | NA | 334 (74.0) | NA | 9.9 ± 7.1 | NA | NA |
| Deharo 2020 | OB(PM) | Evolut R | 10459 | 83.1 ± 6.4 | 4240 (40.5) | 8475 (81.0) | 3088 (29.5) | 4936 (47.2) | 6279 (60.0) | 3725 (35.6) | 4488 (42.9) | 1438 (13.7) | NA | NA | NA |
|  |  | Sapien 3 | 10459 | 83.0 ± 6.6 | 4229 (40.4) | 8454 (80.8) | 3115 (29.8) | 4943 (47.3) | 6242 (59.7) | 3726 (35.6) | 4463 (42.7) | 1459 (13.9) | NA | NA | NA |
| Bisson 2020 | OB | Evolut R | 14446 | 82.7 ± 6.8 | 5866 (40.6) | 12214 (84.5) | 4370 (30.3) | 7323 (50.7) | 9098 (63.0) | 5150 (35.7) | 6345 (43.9) | 3192 (22.1) | NA | 3.8 ± 1.0 | NA |
|  |  | Sapien 3 | 25174 | 82.4 ± 6.9 | 13413 (53.3) | 21312 (84.7) | 7831 (31.1) | 12636 (50.2) | 16218(64.4) | 9144 (36.3) | 11359 (45.1) | 5668 (22.5) | NA | 3.7 ± 1.0 | NA |
| Thiele 2020 | RCT | Evolut R | 219 | 81.7 ± 5.3 | 105 (47.9) | 193 (88.1) | 79 (36.2) | 100 (45.9) | 127 (58.0) | 29 (13.3) | 103 (47.0) | 30 (13.7) | 4.9 (2.9-9.9) | 4.1 (2.5-7.5) | 14.9 (8.9-23.8) |
|  |  | Sapien 3 | 219 | 81.5 ± 5.7 | 109 (49.8) | 204 (93.2) | 68 (31.1) | 80 (36.9) | 116 (52.7) | 26 (11.8) | 93 (42.5) | 29 (13.4) | 4.7 (3.1-9.4) | 3.8 (2.4-6.1) | 14.8 (8.6-24.4) |
| Hase 2020 | OB (PM) | Evolut R | 69 | 86.0 (84.0-89.0) | 11 (15.9) | 52 (75.4) | 8 (11.6) | NA | 10 (14.5) | NA | 11 (15.9) | 18 (26.1) | 6.4 (4.8-8.4) | NA | NA |
|  |  | Sapien 3 | 69 | 87.0 (82.0-89.0) | 12 (17.4) | 52 (75.4) | 6 (8.7) | NA | 12 (17.4) | NA | 7 (10.1) | 21 (30.4) | 6.1 (4.7-8.6) | NA | NA |
| Armijo 2020 | OB | Evolut R | 193 | 81.0 (75.0-86.0) | 177 (91.7) | NA | 58 (30.1) | NA | 107 (55.4) | 43 (22.3) | 79 (41.1) | 60 (31.1) | 4.0 (2.3-7.0) | 4.0 (2.5-7.6) | NA |
|  |  | Sapien 3 | 640 | 80.0 (74.5-84.2) | 602 (94.1) | NA | 180 (28.2) | NA | 372 (58.1) | 113 (17.7) | 270 (42.5) | 155 (24.3) | 3.6 (2.3-6.2) | 3.7 (2.1-6.4) | NA |
| Okuyama 2020 | OB | Evolut R/Pro | 13 | 84.0 (82.5-87.0) | 0 (0.0) | 12 (92.0) | 4 (31.0) | 9 (69.0) | 1 (8.0) | 1 (8.0) | 1 (8.0) | 0 (0.0) | 5.6 (3.6-5.9) | NA | NA |
|  |  | Sapien 3 | 33 | 84.0 (79.5-88.5) | 1 (3.0) | 27 (82.0) | 7 (21.0) | 14 (42.0) | 3 (9.0) | 5 (15.0) | 4 (12.0) | 2 (6.0) | 4.8 (3.5-8.2) | NA | NA |
| Eftychiou 2020 | OB | Evolut R/Pro | 57 | 78.7 ± 6.7 ^#^ | 119 (50.6) ^#^ | NA | 85 (36.2) ^#^ | NA | NA | 28 (11.9) ^#^ | 62 (26.4) ^#^ | 64 (27.2) ^#^ | 5.1 ± 3.6 ^#^ | 5.4 ± 4.2 ^#^ | NA |
|  |  | Sapien 3 | 178 |  |  | NA |  | NA | NA |  |  |  |  |  | NA |
| Fukuda 2020 | OB | Evolut R | 1550 | 85.1 ± 5.2 | 409 (26.4) | 1486 (95.9) | 528 (34.1) | NA | NA | 604 (39.0) | NA | 434 (28.0) | 6.8 ± 2.4 | NA | NA |
|  |  | Sapien 3 | 5276 | 84.6 ± 5.1 | 1654 (31.3) | 5016 (95.1) | 1830 (34.7) | NA | NA | 1908 (36.2) | NA | 1549 (29.4) | 6.8 ± 2.5 | NA | NA |
| Habertheuer 2020 | OB | Evolut R/Pro | 269 | 84.0 (78.0-87.0) | 89 (33.1) | 241 (89.6) | 123 (45.7) | NA | NA | NA | 118 (43.9) | 22 (8.2) | 6.7 (4.7-9.1) | NA | NA |
|  |  | Sapien 3 | 294 | 82.0 (77.0-87.0) | 176 (59.9) | 254 (86.4) | 121 (41.2) | NA | NA | NA | 131 (44.6) | 31 (10.5) | 4.9 (3.6-7.5) | NA | NA |
| Tamm 2021 | OB | Evolut R | 144 | 82.2 ± 6.7 | 49 (34.0) | 118 (81.9) | 55 (38.2) | NA | 87 (60.4) | 16 (11.1) | 32 (22.4) | 14 (9.7) | 7.4 ± 5.0 | 7.8 ± 8.1 | NA |
|  |  | Sapien 3 | 215 | 81.7 ± 6.6 | 118 (54.9) | 196 (91.6) | 59 (27.9) | NA | 137 (63.7) | 17 (7.9) | 55 (25.9) | 23 (10.7) | 7.3 ± 6.8 | 8.2 ± 8.2 | NA |
| Lee 2021 | OB | Evolut R/Pro | 45 | 81.7 ± 5.4 | 1 (2.2) | 37 (82.2) | 18 (40.0) | 31 (68.9) | 22 (48.9) | 11 (24.4) | 9 (20.0) | 4 (8.9) | 5.9 ± 4.0 | NA | NA |
|  |  | Sapien 3 | 25 | 81.7 ± 3.4 | 4 (16.0) | 22 (88.0) | 11 (44.0) | 21 (84.0) | 15 (60.0) | 2 (8.0) | 3 (12.0) | 3 (12.0) | 4.2 ± 1.8 | NA | NA |
| Meguro 2021 | OB | Evolut R | 103 | 85.0 (82.0–88.0) | 1 (1.0) | 80 (77.7) | 21 (20.4) | 56 (54.4) | 29 (28.2) | NA | 16 (15.5) | 5 (4.9) | 6.4 (5.0–9.1) | NA | 15.2 (9.0–24.0) |
|  |  | Sapien 3 | 90 | 84.0 (82.0–88.0) | 3 (3.3) | 69 (76.7) | 26 (28.9) | 42 (46.7) | 16 (17.8) | NA | 11 (12.2) | 3 (3.3) | 5.8 (4.6–8.1) | NA | 11.5 (8.5–16.1) |
| Paitazoglou 2021 | OB | Evolut R | 124 | 81.9 ± 0.5 | 62 (50.0) | 110 (88.7) | 30 (24.2) | 86 (69.4) | 78 (62.9) | NA | 54 (43.5) | NA | NA | NA | 22.5 ± 0.8 |
|  |  | Sapien 3 | 129 | 82.2 ± 0.5 | 63 (48.8) | 122 (94.6) | 30 (23.3) | 93 (72.1) | 63 (48.8) | NA | 52 (40.0) | NA | NA | NA | 21.8 ± 0.9 |
| Kanso 2021 | OB | Evolut R | 49 | 83.8 ± 7.0 | 16 (32.7) | 47 (95.9) | 21 (42.9) | NA | NA | 15 (30.6) | 21 (42.9) | 5 (10.2) | NA | 4.1 ± 2.7 | 18.8 ± 11.6 |
|  |  | Sapien 3 | 161 | 84.5 ± 6.1 | 88 (54.7) | 136 (84.5) | 51 (31.7) | NA | NA | 33 (20.5) | 39 (24.2) | 16 (9.9) | NA | 4.8 ± 5.3 | 22.1 ± 13.3 |
| Fukui 2021 | OB | Evolut R/Pro | 171 | 84.0 (79.0–87.0) | 85 (49.7) | 151 (88.3) | 47 (27.5) | NA | 75 (43.9) | NA | 60 (35.1) | NA | 3.7 (2.5–5.5) | NA | NA |
|  |  | Sapien 3 | 276 | 82.0 (77.0–87.0) | 157 (56.9) | 238 (86.2) | 84 (30.4) | NA | 113 (40.9) | NA | 101 (36.6) | NA | 3.3 (2.3–4.8) | NA | NA |
| Medranda 2021 | OB | Evolut Pro | 340 | 78.7 ± 9.2 | 155 (45.6) | 299 (87.9) | 124 (36.5) | NA | 180 (52.9) | 47 (13.8) | NA | NA | 4.9 ± 3.9 | NA | NA |
|  |  | Sapien 3 | 501 | 79.1 ± 9.2 | 302 (60.3) | 446 (89.0) | 183 (36.5) | NA | 319 (63.7) | 97 (19.4) | NA | NA | 5.3 ± 3.6 | NA | NA |
| Minha 2021 | OB | Evolut R/Pro | 276 | 83.0 (79.2-86.3) | 81 (29.3) | 238 (86.2) | 106 (38.4) | 209 (75.7) | 121 (44.0) | NA | 68 (24.6) | 26 (9.4) | 3.4 (2.6-5.0) | 3.1 (2.3-5.3) | NA |
|  |  | Sapien 3 | 121 | 81.3 (78.3-85.4) | 73 (60.3) | 97 (80.2) | 46 (38.0) | 69 (57.0) | 68 (56.2) | NA | 27 (22.3) | 14 (11.6) | 3.0 (2.2-4.6) | 2.6 (1.8-4.9) | NA |
| Catalano 2020 | OB | Evolut R/Pro | 164 | 83.8^#^ | 149 (48.8) ^#^ | 329 (95.1) ^#^ | 121 (35.0) ^#^ | NA^#^ | NA^#^ | 52 (15.0) ^#^ | NA^#^ | 87 (25.1) ^#^ | 4.8^#^ | NA^#^ | NA^#^ |
|  |  | Sapien 3 | 182 |  |  |  |  |  |  |  |  |  |  |  |  |
| Mauri 2017 | OB (PM) | Acurate Neo | 92 | 82.8 ± 6.5 | 7 (7.6) | NA | NA | NA | NA | NA | NA | NA | NA | NA | 15.8 ± 9.1 |
|  |  | Sapien 3 | 92 | 81.9 ± 5.3 | 7 (7.6) | NA | NA | NA | NA | NA | NA | NA | NA | NA | 18.7 ± 11.9 |
| Schaefer 2017 | OB (PM) | Acurate Neo | 104 | 81.7 ± 5.5 | 32 (30.8) | 89 (85.6) | 29 (27.9) | NA | 62 (59.6) | NA | NA | 18 (17.3) | 5.8 ± 3.8 | NA | 15.9 ± 9.3 |
|  |  | Sapien 3 | 104 | 81.2 ± 6.2 | 36 (34.6) | 97 (93.3) | 27 (26.0) | NA | 60 (57.7) | NA | NA | 21 (20.2) | 5.4 ± 3.6 | NA | 13.7 ± 9.0 |
| Husser 2017 | OB (PM) | Acurate Neo | 311 | 81.0 ± 6.0 | 122 (39.2) | NA | 103 (33.1) | NA | 190 (61.1) | 33 (10.6) | NA | 42 (13.5) | NA | NA | 18.0 ± 10.0 |
|  |  | Sapien 3 | 622 | 81.0 ± 6.0 | 278 (44.7) | NA | 201 (32.3) | NA | 390 (62.7) | 70 (11.3) | NA | 92 (14.8) | NA | NA | 18.0 ± 12.0 |
| Pollari 2018 | OB | Acurate | 97 | 81.6 ± 5.5 | 45 (46.0) | NA | NA | NA | NA | NA | NA | 16 (16.0) | NA | 8.0 ± 0.1 | 22.0 ± 0.1 |
|  |  | Sapien 3 | 206 | 82.0 ± 5.5 | 103 (50.0) | NA | NA | NA | NA | NA | NA | 38 (18.0) | NA | 9.0 ± 0.1 | 23.0 ± 0.2 |
| Husser 2019 | OB (PM) | Acurate Neo | 65 | 81.0 (77.0-84.0) | 25 (38.5) | 60 (92.3) | 21 (32.3) | NA | 45 (69.2) | 9 (13.8) | 14 (21.5) | 10 (15.4) | NA | NA | 14.3 (9.8–21.5) |
|  |  | Sapien 3 | 65 | 82.0 (77.0-86.0) | 25 (38.5) | 54 (83.1) | 20 (30.8) | NA | 45 (69.2) | 17 (26.2) | 18 (27.7) | 13 (20.0) | NA | NA | 14.9 (9.0–26.1) |
| Lanz 2019 | RCT | Acurate Neo | 372 | 82.6 ± 4.3 | 154 (41) | 341 (92.0) | 108 (29.0) | 211 (57.0) | 218 (59.0) | NA | 133 (36.0) | 33 (9.0) | 3.7 (2.5-4.9) | NA | NA |
|  |  | Sapien 3 | 367 | 83.0 ± 3.9 | 165 (45) | 333 (91.0) | 116 (32.0) | 216 (59.0) | 219 (60.0) | NA | 136 (37.0) | 44 (12.0) | 3.4 (2.6-5.2) | NA | NA |
| Moriyama 2019 | OB | Acurate Neo | 146 | 80.7 ± 6.0 | 66 (45.2) | 133 (91.0) | 36 (26.0) | 113 (77.0) | NA | 21 (14.0) | 55 (38.0) | 31 (23.0) | 4.7 ± 3.5 | 4.1 ± 3.1 | NA |
|  |  | Sapien 3 | 103 | 79.6 ± 7.3 | 51 (49.5) | 87 (84.0) | 27 (26.0) | 76 (72.0) | NA | 17 (17.0) | 40 (39.0) | 30 (29.0) | 4.3 ± 3.6 | 4.6 ± 3.6 | NA |
| Barth 2019 | OB (PM) | Acurate | 329 | 81.0 ± 5.0 | 145 (44.1) | 307 (93.3) | 121(36.8) | NA | NA | NA | 125(38.0) | 52 (15.8) | NA | NA | 18.8 ± 14.7 |
|  |  | Sapien 3 | 329 | 81.0 ± 6.0 | 146 (44.4) | 306 (93.0) | 115(35.0) | NA | NA | NA | 127 (38.7) | 49 (14.9) | NA | NA | 19.1 ± 13.6 |
| Kooistra 2020 | OB | Acurate Neo | 78 | 81.2 ± 5.6 | 20 (25.6) | 52 (66.7) | 24 (30.8) | 44 (56.4) | 39 (50.0) | 5 (6.4) | 20 (25.6) | 12 (15.4) | NA | NA | 13.4 ± 7.6 |
|  |  | Sapien 3 | 230 | 80.2 ± 7.2 | 126 (54.8) | 151 (65.7) | 60 (26.1) | 127 (55.2) | 95 (41.3) | 40 (17.4) | 83 (36.1) | 44 (19.1) | NA | NA | 14.8 ± 10.6 |
| Mas-Peiro 2019 | OB (PM) | Portico | 104 | 81.8 ± 4.9 | 61 (58.7) | NA | 41 (39.4) | NA | 61 (58.7) | NA | NA | 14 (13.5) | 3.9 ± 2.2 | 4.7 ± 3.9 | NA |
|  |  | Sapien 3 | 73 | 81.5 ± 7.3 | 48 (65.8) | NA | 22 (30.1) | NA | 43 (58.9) | NA | NA | 9 (12.2) | 3.9 ± 2.9 | 5.2 ± 4.5 | NA |
| Wöhrle 2015 | OB | Lotus | 26 | 79.3 ± 5.3 | 10 (38.5) | NA | 6 (23.0) | NA | 7 (27.0) | 6 (23.0) | 10 (38.0) | 16 (62.0) | 7.3 ± 5.3 ^#^ | NA | 17.7 ± 11.8 ^#^ |
|  |  | Sapien 3 | 52 | 82.6 ± 6.2 | 27 (51.9) | NA | 16 (31.0) | NA | 21 (40.0) | 10 (19.0) | 22 (42.0) | 35 (67.0) |  | NA |  |
| Rodríguez-Olivares 2016 | OB | Lotus | 35 | NA | NA | NA | NA | NA | NA | NA | NA | NA | NA | NA | NA |
|  |  | Sapien 3 | 26 | NA | NA | NA | NA | NA | NA | NA | NA | NA | NA | NA | NA |
| Soliman 2016 | OB | Lotus | 79 | 80.0 ± 7.0 | 35 (44.0) | NA | NA | NA | NA | NA | NA | NA | NA | NA | 14.0 ± 9.3 |
|  |  | Sapien 3 | 83 | 80.0 ± 8.0 | 45 (45.0) | NA | NA | NA | NA | NA | NA | NA | NA | NA | 15.5 ± 9.5 |
| Pilgrim 2016 | OB | Lotus | 140 | 83.0 ± 5.4 | 75 (53.6) | 114 (81.4) | 33 (23.6) | 80 (57.1) | 85 (60.7) | 11 (7.9) | NA | 11 (7.9) | 4.1 ± 2.4 | NA | 15.0 ± 8.6 |
|  |  | Sapien 3 | 815 | 81.9 ± 6.4 | 463 (56.8) | 625 (76.8) | 200 (24.5) | 392 (48.1) | 477 (58.5) | 126 (15.5) | NA | 91 (11.2) | 5.0 ± 3.8 | NA | 18.9 ± 14.8 |
| Vangils 2017 | OB | Lotus | 20 | 83.0 ± 6.0 | 12 (60.0) | NA | 9 (45.0) | NA | NA | 6 (30.0) | 4 (20.0) | 5 (25.0) | 5.9 (5.2-7.8) | NA | NA |
|  |  | Sapien 3 | 32 | 81.0 ± 6.0 | 20 (63.0) | NA | 13 (41.0) | NA | NA | 7 (22.0) | 8 (25.0) | 7 (22.0) | 4.5 (3.0-10.5) | NA | NA |
| Jarr 2017 | OB | Lotus | 63 | 81.3 ± 5.1 | 42 (66.7) | NA | NA | NA | NA | NA | NA | NA | 5.6 ± 4.5 | NA | NA |
|  |  | Sapien 3 | 90 | 81.7 ± 7.3 | 66 (73.3) | NA | NA | NA | NA | NA | NA | NA | 5.3 ± 4.8 | NA | NA |
| Seeger 2017 | OB (PM) | Lotus | 202 | 81.2 ± 5.2 | 88 (43.6) | NA | 52 (25.9) | NA | 122 (60.7) | 160 (81.7) | 74 (36.6) | 91 (45.1) | 6.8 ± 5.0 | NA | 13.2 ± 12.1 |
|  |  | Sapien 3 | 202 | 80.1 ± 6.4 | 86 (42.6) | NA | 52 (25.9) | NA | 124 (61.4) | 165 (81.7) | 73 (36.1) | 75 (37.2) | 6.5 ± 5.2 | NA | 14.6 ± 13.0 |
| Seeger 2017 ^2^ | OB | Lotus | 100 | 80.6 ± 5.9 ^#^ | 92 (46.0) ^#^ | NA | 57 (28.5) ^#^ | NA | 124 (62.0) ^#^ | 151 (22.0) ^#^ | 85 (42.5) ^#^ | NA | 6.5 ± 4.4 ^#^ | NA | NA |
|  |  | Sapien 3 | 100 |  |  | NA |  | NA |  |  |  | NA |  | NA | NA |
| Schofer 2018 | OB | Lotus | 61 | 80.5 ± 7.5 | 26 (42.6) | NA | 10 (16.4) | NA | 37 (61.7) | 12 (19.7) | NA | 8 (13.1) | 4.8 ± 2.6 | NA | 13.4 ± 8.6 |
|  |  | Sapien 3 | 212 | 80.6 ± 7.2 | 110 (51.9) | NA | 31 (14.6) | NA | 126 (59.4) | 30 (14.2) | NA | 40 (18.9) | 5.9 ± 5.6 | NA | 16.1 ± 11.0 |
| Naifovino 2018 | OB (PM) | Lotus | 93 | 80.5 ± 7.0 | 52 (55.9) | 77 (82.8) | 26 (28.0) | NA | 43 (46.2) | NA | 21 (22.6) | 27 (29.0) | 9.0 ± 5.2 | NA | 15.9 ± 10.2 |
|  |  | Sapien 3 | 93 | 79.8 ± 5.8 | 54 (58.1) | 75 (80.6) | 23 (24.7) | NA | 48 (51.6) | NA | 25 (26.9) | 29 (31.2) | 8.7 ± 8.0 | NA | 15.3 ± 10.5 |
| Sathananthan 2018 | OB | Lotus | 72 | NA | NA | NA | NA | NA | NA | NA | NA | NA | NA | NA | NA |
|  |  | Sapien 3 | 136 | NA | NA | NA | NA | NA | NA | NA | NA | NA | NA | NA | NA |
| Schulz 2017 | OB | DMF | 61 | 81.4 ± 0.6 | 21 (34.0) | NA | 8 (13.0) | NA | 45 (74.0) | 3 (5.0) | 12 (20.0) | 5 (8.0) | NA | NA | 29.6 ± 1.7 |
|  |  | Sapien 3 | 113 | 82.3 ± 0.6 | 60 (53.0) | NA | 30 (27.0) | NA | 74 (65.0) | 17 (15.0) | 21 (19.0) | 22 (19.0) | NA | NA | 31.7 ± 1.4 |
| Gamal 2019 | OB | DMF | 68 | 82.4 ± 5.8 | 43 (63.2) | NA | 11 (16.2) | NA | NA | NA | 24 (35.3) | 23 (33.8) | NA | NA | 6.3 ± 1.8 |
|  |  | Sapien 3 | 99 | 80.0 ± 7.1 | 48 (48.5) | NA | 26 (26.3) | NA | NA | NA | 28 (28.3) | 30 (30.3) | NA | NA | 8.0 ± 2.1 |
| Edlinge 2021 | OB (PM) | DMF | 42 | 81.7 ± 5.2 | 19 (45.2) | NA | 15 (35.7) | NA | NA | NA | NA | NA | NA | NA | 15.7 ± 6.8 |
|  |  | Sapien 3 | 42 | 80.7 ± 5.9 | 12 (28.6) | NA | 16 (38.1) | NA | NA | NA | NA | NA | NA | NA | 15.3 ± 14.4 |
| Pagnesi 2019 | OB (PM) | Evolut Pro | 251 | 81.6 ± 6.1 | 86 (34.3) | 224 (89.2) | 72 (28.7) | NA | NA | 62 (24.7) | 93 (37.1) | 46 (18.3) | 5.1 ± 3.1 | 6.3 ± 5.2 | NA |
|  |  | Acurate Neo | 251 | 81.4 ± 6.5 | 86 (34.3) | 204 (87.6) | 68 (27.1) | NA | NA | 62 (24.7) | 91 (36.3) | 46 (18.3) | 5.3 ± 3.7 | 5.8 ± 6.0 | NA |
| Tamburino 2020 | RCT | Evolut | 398 | 82.9 ± 4.3 | 123 (31.0) | 328 (83.0) | 113 (29.0) | 192 (49.0) | 149 (38.0) | 41(10.0) | 126 (32.0) | 55 (14.0) | 4.5 ± 2.7 | NA | NA |
|  |  | Acurate Neo | 398 | 83.4 ± 4.2 | 135 (34.0) | 350 (88.0) | 108 (27.0) | 212 (53.0) | 171 (43.0) | 30 (8.0) | 138 (35.0) | 37 (9.0) | 4.6 ± 3.0 | NA | NA |
| Ivanov 2021 | OB (PM) | Evolut R | 76 | 81.2 ± 5.6 | 38 (50.0) | NA | 24 (32.0) | NA | 48 (63.0) | 12 (16.0) | 37 (49.0) | 19 (25.0) | 10 (8, 12) | NA | 21 (16, 26) |
|  |  | Acurate Neo | 76 | 81.4 ± 4.5 | 39 (51.3) | NA | 26 (34.0) | NA | 49 (64.5) | 12 (16.0) | 38 (50.0) | 18 (24.0) | 9 (7, 12) | NA | 19 (14, 24) |
| Gorla 2019 | OB | Evolut R | 263 | 83.6 ± 6.9 ^#^ | 212 (56.7) ^#^ | NA | NA | NA | 180 (48.1) ^#^ | NA | NA | NA | 5.9 ± 3.8 ^#^ | NA | NA |
|  |  | Portico | 111 |  |  | NA | NA | NA |  | NA | NA | NA |  | NA | NA |
| Corcione 2020 | OB | Evolut R/Pro | 119 | 83.2 (79.1-85.5) | 51 (42.9) | 87 (73.0) | 14 (12.0) | 63 (53.0) | 7 (6.0) | NA | NA | 30 (26.0) | NA | 2.8 (2.1-4.0) | 19.0 (14.0-26.0) |
|  |  | Portico | 114 | 82.7 (79.1-85.1) | 44 (38.6) | 80 (70.0) | 17 (15.0) | 64 (56.0) | 10 (9.0) | NA | NA | 16 (14.0) | NA | 2.2 (1.9-3.3) | 16.0 (12.0-19.0) |
| Gama 2022 | OB | Acurate | 32 | NA | NA | NA | NA | NA | NA | NA | NA | NA | NA | NA | NA |
|  |  | Portico | 49 | NA | NA | NA | NA | NA | NA | NA | NA | NA | NA | NA | NA |
| Feldman 2018 | RCT | Evolut R | 135 | NA | NA | NA | NA | NA | NA | NA | NA | NA | NA | NA | NA |
|  |  | Lotus | 529 | NA | NA | NA | NA | NA | NA | NA | NA | NA | NA | NA | NA |
| Giannini 2017 | OB | Lotus | 60 | 82.0 ± 5.0 | 33 (55.0) | 47 (78.3) | 18 (30.0) | NA | 36 (60.0) | 14 (23.3) | 15 (25.0) | 15 (25.0) | 6.0 ± 5.0 | NA | 18.0 ± 10.0 |
|  |  | DFM | 115 | 82.0 ± 8.0 | 57 (49.6) | 89 (77.4) | 25 (21.7) | NA | 68 (59.1) | 30 (26.1) | 23 (20.0) | 22 (19.1) | 6.0 ± 7.0 | NA | 20.0 ± 13.0 |
| Costa 2019 | OB (PM) | Evolut R | 48 | 83.0 (80.0-85.0) | 15 (31.3) | 41 (85.4) | 11 (22.9) | 26 (54.2) | NA | 2 (4.2) | 10 (20.8) | 7 (14.6) | 3.9 ± 2.3 | NA | NA |
|  |  | Acurate Neo | 48 | 82.0 (80.0-85.0) | 14 (29.2) | 43 (89.6) | 9 (18.8) | 24 (50.0) | NA | 3 (6.3) | 6 (12.5) | 10 (20.8) | 4.0 ± 3.3 | NA | NA |
|  |  | Sapien 3 | 48 | 83.0 (82.0-85.0) | 15 (31.3) | 43 (89.6) | 13 (27.1) | 28 (58.3) | NA | 2 (4.2) | 6 (12.5) | 7 (14.6) | 3.8 ± 1.7 | NA | NA |
| Makkar 2020 | RCT | Evolut R/Pro | 110 | 83.3 ± 6.7 | 50 (45.5) | 100 (90.1) | 46 (41.4) | NA | 74 (66.7) | 19 (17.1) | 38 (34.2) | 45 (40.5) | 6.5 (3.1) | 5.2 (4.4) | NA |
|  |  | Portico | 375 | 83.0 ± 7.6 | 182 (48.5) | 354 (94.4) | 140 (37.3) | NA | 261 (69.6) | 70 (18.7) | 122 (32.5) | 156 (41.6) | 6.3 (3.4) | 6.6 (7.2) | NA |
|  |  | Sapien 3 | 206 | 83.5 ± 7.4 | 99 (48.1) | 184 (89.3) | 73 (35.4) | NA | 150 (72.8) | 29 (14.1) | 81 (39.3) | 81 (39.3) | 6.2 (3.4) | 6.8 (5.9) | NA |
| Tichelba¨cker 2018 | OB | Portico | 16 | NA | NA | NA | NA | NA | NA | NA | NA | NA | NA | NA | NA |
|  |  | DFM | 31 | NA | NA | NA | NA | NA | NA | NA | NA | NA | NA | NA | NA |
|  |  | Sapien 3 | 134 | NA | NA | NA | NA | NA | NA | NA | NA | NA | NA | NA | NA |
| Regazzoli 2019 | OB | Evolut R/Pro | 481 | 82.3 ± 0.5 | 52 (10.8) | 407 (84.6) | 124 (25.8) | 242 (50.3) | 176 (36.6) | 88 (18.3) | 103 (21.4) | 55 (11.4) | 5.8 ± 0.3 | NA | NA |
|  |  | Acurate | 201 | 82.7 ± 0.4 | 21 (10.5) | 162 (80.6) | 55 (27.4) | 86 (42.8) | 80 (39.8) | 30 (14.9) | 47 (23.4) | 26 (12.9) | 5.7 ± 0.3 | NA | NA |
|  |  | Portico | 177 | 82.7 ± 0.5 | 14 (7.9) | 150 (84.8) | 55 (31.1) | 90 (51.1) | 62 (35.2) | 22 (12.6) | 50 (28.3) | 12 (6.8) | 5.3 ± 0.2 | NA | NA |
| Gorla 2020 | OB | Evolut R | 211 | 82.6 ± 6.8 | 102 (48.3) | 157 (74.4) | 74 (35.1) | 80 (37.9) | 82 (38.9) | NA | NA | 42 (19.9) | 5.6 ± 3.6 | NA | NA |
|  |  | Acurate Neo | 85 | 82.1 ± 8.1 | 39 (45.9) | 60 (70.6) | 20 (23.5) | 34 (40) | 15 (17.6) | NA | NA | 13 (15.3) | 4.3 ± 2.9 | NA | NA |
|  |  | Portico | 96 | 83.5 ± 6.6 | 32 (33.3) | 74 (77.1) | 25 (26.0) | 44 (45.8) | 27 (28.1) | NA | NA | 7 (7.3) | 5.6 ± 3.7 | NA | NA |
| Vera Vera 2020 | OB (PM) | Evolut R/Pro | 217 | 81.4 ± 6.9 | 112 (51.6) | 165 (76) | 83 (38.2) | 120 (55.3) | 98 (45.2) | 22 (10.3) | 90 (41.5) | 46 (21.2) | 5.0 ± 4.1 | NA | 15.9 ± 11.4 |
|  |  | Acurate Neo | 107 | 81.5 ± 6.2 | 38 (35.5) | 87 (81.3) | 37 (34.6) | 66 (61.7) | 34 (31.8) | 4 (3.8) | 34 (31.8) | 11 (10.4) | 4.4 ± 1.4 | NA | 13.4 ± 8.3 |
|  |  | Portico | 88 | 82.4 ± 5.9 | 30 (34.1) | 77 (87.5) | 31 (35.2) | 60 (68.2) | 24 (27.3) | 3 (3.6) | 43 (48.9) | 6 (6.8) | 4.9 ± 2.7 | NA | 15.2 ± 9.0 |
| Bieliauskas 2021 | OB (PM) | Evolut | 20 | 78.0 (75.0-81.0) | 17 (85.0) | 15 (75.0) | 2 (10.0) | NA | 7 (35.0) | 1 (5.0) | 7 (35.0) | 4 (20.0) | 1.9 (1.3-2.4) | NA | NA |
|  |  | Acurate Neo | 20 | 78.0 (73.0-83.0) | 9 (45.0) | 16 (80.0) | 4 (20.0) | NA | 10 (50.0) | 1 (5.0) | 5 (25.0) | 3 (15.0) | 2.4 (1.5-2.8) | NA | NA |
|  |  | Portico | 20 | 79.0 (74.0-85.0) | 13 (65.0) | 12 (60.0) | 5 (20.0) | NA | 9 (45.0) | 4 (20.0) | 4 (20.0) | 5 (25.0) | 2.1 (1.5-2.9) | NA | NA |
| Leone 2021 | OB (PM) | Evolut | 248 | NA | NA | NA | NA | NA | NA | NA | NA | NA | NA | NA | NA |
|  |  | Acurate Neo | 104 | NA | NA | NA | NA | NA | NA | NA | NA | NA | NA | NA | NA |
|  |  | Portico | 93 | NA | NA | NA | NA | NA | NA | NA | NA | NA | NA | NA | NA |
| Kim 2017 | OB | Evolut R | 15 | NA | NA | NA | NA | NA | NA | NA | NA | NA | NA | NA | NA |
|  |  | Acurate Neo | 425 | NA | NA | NA | NA | NA | NA | NA | NA | NA | NA | NA | NA |
|  |  | Portico | 127 | NA | NA | NA | NA | NA | NA | NA | NA | NA | NA | NA | NA |
|  |  | Sapien 3 | 381 | NA | NA | NA | NA | NA | NA | NA | NA | NA | NA | NA | NA |
| Mauri 2019 | OB | Evolut R | 132 | 83.1 ± 5.5 | 36 (27.3) | 121 (91.7) | 39 (29.5) | NA | 83 (62.9) | 17 (12.9) | 57 (43.2) | 15 (11.4) | NA | 4.3 ± 3.3 | 21.5 ± 13.6 |
|  |  | Acurate Neo | 166 | 82.9 ± 4.8 | 43 (25.9) | 157 (94.6) | 54 (32.5) | NA | 111 (66.9) | 33 (19.9) | 63 (38.2) | 21 (12.7) | NA | 4.3 ± 4.1 | 22.1 ± 15.0 |
|  |  | Lotus | 52 | 81.4 ± 5.7 | 24 (46.2) | 48 (92.3) | 15 (28.8) | NA | 34 (65.4) | 9 (17.3) | 19 (36.5) | 8 (15.4) | NA | 4.7 ± 4.6 | 19.8 ± 15.7 |
|  |  | Sapien 3 | 292 | 81.2 ± 6.4 | 165 (56.5) | 258 (88.4) | 92 (31.5) | NA | 174 (59.6) | 73 (25.0) | 123 (42.1) | 66 (22.6) | NA | 4.8 ± 4.4 | 24.3 ± 17.2 |
| Modolo 2020 | OB | Evolut R/Pro | 390 | NA | NA | NA | NA | NA | NA | NA | NA | NA | NA | NA | NA |
|  |  | Acurate | 115 | NA | NA | NA | NA | NA | NA | NA | NA | NA | NA | NA | NA |
|  |  | Lotus | 546 | NA | NA | NA | NA | NA | NA | NA | NA | NA | NA | NA | NA |
|  |  | Sapien 3 | 397 | NA | NA | NA | NA | NA | NA | NA | NA | NA | NA | NA | NA |
| Stundl 2019 | OB | Evolut R | 114 | 81.3 ± 5.7 | 32 (28.1) | NA | 29 (25.4) | NA | 59 (51.8) | NA | 50 (43.9) | 17 (15.0) | 4.2 (2.9-5.5) | 4.9 (3.0-7.6) | 16.9 (11.2-24.8) |
|  |  | Lotus | 104 | 80.4 ± 6.6 | 52 (50.0) | NA | 27 (26.0) | NA | 60 (57.7) | NA | 44 (42.3) | 14 (13.5) | 3.9 (2.7-5.8) | 4.6 (3.0-7.7) | 12.3 (8.4-20.3) |
|  |  | DFM | 38 | 80.9 ± 7.3 | 28 (73.7) | NA | 13 (34.2) | NA | 27 (71.1) | NA | 21 (55.3) | 9 (23.7) | 4.7 (3.2-7.7) | 4.6 (3.1-13.5) | 19.9 (10.6-33.3) |
|  |  | Sapien 3 | 101 | 81.0 ± 5.7 | 63 (62.4) | NA | 29 (28.7) | NA | 65 (64.4) | NA | 47 (46.5) | 21 (20.8) | 3.6 (2.3-5.1) | 4.7 (2.9-7.4) | 13.5 (9.4-22.2) |
| Panoulas 2020 | OB | Evolut R | 22 | NA | 0 (0.0) | NA | NA | NA | NA | NA | NA | NA | NA | NA | NA |
|  |  | Lotus | 25 | NA | 0 (0.0) | NA | NA | NA | NA | NA | NA | NA | NA | NA | NA |
|  |  | DFM | 25 | NA | 0 (0.0) | NA | NA | NA | NA | NA | NA | NA | NA | NA | NA |
|  |  | Sapien 3 | 48 | NA | 0 (0.0) | NA | NA | NA | NA | NA | NA | NA | NA | NA | NA |
| Nicolas 2021 | OB | Evolut R | 70 | NA | 0 (0.0) | NA | NA | NA | NA | NA | NA | NA | NA | NA | NA |
|  |  | Lotus | 54 | NA | 0 (0.0) | NA | NA | NA | NA | NA | NA | NA | NA | NA | NA |
|  |  | DFM | 29 | NA | 0 (0.0) | NA | NA | NA | NA | NA | NA | NA | NA | NA | NA |
|  |  | Sapien 3 | 209 | NA | 0 (0.0) | NA | NA | NA | NA | NA | NA | NA | NA | NA | NA |
| Santos-Martinez 2022 | OB | Evolut R | 298 | 81.2 ± 6.7 | 158 (50.0) | NA | 114 (38.4) | NA | 122 (40.9) | 27 (9.2) | NA | 56 (18.8) | NA | 5.1 ± 5.2 | NA |
|  |  | Acurate | 180 | 81.4 ± 6.0 | 67 (37.2) | NA | 59 (32.8) | NA | 54 (30.0) | 9 (5.0) | NA | 16 (8.9) | NA | 3.9 ± 3.1 | NA |
|  |  | Portico | 125 | 82.6 ± 5.7 | 46 (36.8) | NA | 44 (35.2) | NA | 38 (30.4) | 6 (5.0) | NA | 6 (4.8) | NA | 6.2 ± 6.1 | NA |
|  |  | Sapien 3 | 290 | 80.4 ± 6.4 | 170 (58.6) | NA | 90 (31.0) | NA | 131 (45.2) | 39 (13.4) | NA | 40 (13.8) | NA | 4.4 ± 4.3 | NA |
| Marzahn 2017 | OB | Portico | 26 | NA | NA | NA | NA | NA | NA | NA | NA | NA | NA | NA | NA |
|  |  | Lotus | 14 | NA | NA | NA | NA | NA | NA | NA | NA | NA | NA | NA | NA |
|  |  | DFM | 41 | NA | NA | NA | NA | NA | NA | NA | NA | NA | NA | NA | NA |
|  |  | Sapien 3 | 234 | NA | NA | NA | NA | NA | NA | NA | NA | NA | NA | NA | NA |
| Giordano 2019 | OB | Evolut | 703 | 82.1 ± 6.7 | 312 (44.4) | NA | NA | NA | NA | NA | NA | NA | 5.6 ± 4.1 | 5.0 ± 4.9 | 16.7 ± 11.6 |
|  |  | Acurate | 234 | 83.5 ± 6.0 | 78 (33.3) | NA | NA | NA | NA | NA | NA | NA | 5.0 ± 3.8 | 4.1 ± 4.2 | 12.9 ± 11.0 |
|  |  | Portico | 347 | 82.5 ± 6.5 | 14 (35.7) | NA | NA | NA | NA | NA | NA | NA | 6.3 ± 4.2 | 4.2 ± 3.9 | 16.2 ± 11.6 |
|  |  | Lotus | 151 | 82.0 ± 6.5 | 68 (45.0) | NA | NA | NA | NA | NA | NA | NA | 5.1 ± 4.2 | 5.0 ± 6.1 | 16.3 ± 14.5 |
|  |  | Sapien 3 | 541 | 83.1 ± 6.5 | 253 (46.8) | NA | NA | NA | NA | NA | NA | NA | 5.4 ± 4.2 | 5.5 ± 4.8 | 16.7 ± 12.1 |
| Okuno 2020 | OB | Evolut | 237 | NA | NA | NA | NA | NA | NA | NA | NA | NA | NA | NA | NA |
|  |  | Acurate | 170 | NA | NA | NA | NA | NA | NA | NA | NA | NA | NA | NA | NA |
|  |  | Portico | 23 | NA | NA | NA | NA | NA | NA | NA | NA | NA | NA | NA | NA |
|  |  | Lotus | 120 | NA | NA | NA | NA | NA | NA | NA | NA | NA | NA | NA | NA |
|  |  | Sapien 3 | 468 | NA | NA | NA | NA | NA | NA | NA | NA | NA | NA | NA | NA |
| Voigtlände 2021 | OB | Evolut | 179 | 83.0 (79.8–6.8) | 11 (6.1) | NA | 38 (21.2) | NA | 97 (54.2) | NA | NA | 34 (19.0) | 4.7 (3.2–6.5) | NA | NA |
|  |  | Acurate | 428 | 82.4 (79.4–85.7) | 30 (7.0) | NA | 110 (25.7) | NA | 230 (54.0) | NA | NA | 78 (18.2) | 4.4 (3.2–6.5) | NA | NA |
|  |  | Portico | 110 | 83.3 (80.2–85.8) | 4 (3.6) | NA | 37 (33.6) | NA | 56 (50.9) | NA | NA | 12 (10.9) | 4.9 (3.1–6.6) | NA | NA |
|  |  | Lotus | 64 | 83.0 (80.0–86.7) | 6 (9.4) | NA | 12 (18.8) | NA | 27 (42.9) | NA | NA | 6 (9.4) | 4.0 (3.0–5.2) | NA | NA |
|  |  | Sapien 3 | 288 | 82.8 (78.8–85.9) | 24 (8.3) | NA | 69 (24.0) | NA | 164 (56.9) | NA | NA | 54 (18.8) | 4.5 (3.2–6.6) | NA | NA |

Continuous variables are presented as mean value ± standard deviation or median and interquartile range (IQR), categorical variables are presented as n (%). Abbreviations: OB: observational study; PM: propensity score matched study; PN: patient number; HT: hypertension; DM: diabetes mellitus; HLP: hyperlipidemia; CAD: coronary artery disease; PVD: peripheral vascular disease; AF: atrial fibrillation; COPD: chronic obstructive pulmonary disease; STS: Society of Thoracic Surgeons; NA: not available; #: overall.

**Table 3. Baseline imaging and procedural characteristics of the included studies**

| **Study** | **Design** | **Treatment** | **PN** | **Echocardiographic variables** | | | **MDCT variables** | | | **Procedural variables** | | | |
| --- | --- | --- | --- | --- | --- | --- | --- | --- | --- | --- | --- | --- | --- |
|  |  |  |  | **AVA (cm²)** | **MAVG (mmHg)** | **LVEF (%)** | **Annulus**  **diameter (mm)** | **Annulus perimeter (mm)** | **Annulus**  **area (mm²)** | **General anesthesia** | **Transfemoral** | **Pre-dilatation** | **Post-dilatation** |
| Ben-shoshan 2016 | OB | Evolut R | 108 | 0.72 ± 0.18 | 44.7 ± 15.5 | 56.2 ± 9.7 | NA | 23.1 ± 5.6 | 392 ± 62 | NA | 108 (100.0) | 28 (25.9) | 25 (23.1) |
|  |  | Sapien 3 | 124 | 0.72 ± 0.17 | 46.0 ± 15.0 | 55.6 ± 8.5 | NA | 24.1 ± 2.2 | 465 ± 90 | NA | 124 (100.0) | 90 (72.5) | 9 (7.2) |
| Enríquez-Rodríguez 2017 | OB (PM) | Evolut R | 64 | 0.68 ± 0.20 | 47.0 ± 14.0 | 5 (8) < 40% | 24.3 ± 2.4 | 77.0 ± 8.0 | 459 ± 93 | NA | 61 (95.0) | 25 (39.0) | 14 (22.0) |
|  |  | Sapien 3 | 80 | 0.69 ± 0.20 | 45.0 ± 15.0 | 13 (17) < 40% | 24.2 ± 3.1 | 77.0 ± 8.0 | 451 ± 98 | NA | 75 (94.0) | 29 (36.0) | 4 (5.0) |
| Rogers 2017 | OB | Evolut R | 74 | 0.68 ± 0.13 | 48.1 ± 12.2 | 55.0 ± 12.0 | NA | NA | NA | 11 (14.9) | 66 (89.2) | 27 (36.7) | 32 (42.6) |
|  |  | Sapien 3 | 183 | 0.70 ± 0.17 | 45.8 ± 12.0 | 53.0 ± 13.0 | NA | NA | NA | 17 (9.3) | 167 (91.3) | 120 (65.4) | 34 (18.4) |
| Vlastra 2018 | OB (PM) | Evolut | 1091 | NA | NA | NA | NA | NA | NA | NA | 1091 (100.0) | NA | NA |
|  |  | Sapien 3 | 1122 | NA | NA | NA | NA | NA | NA | NA | 1122 (100.0) | NA | NA |
| Veulemans 2018 | OB | Evolut R | 101 | 0.70 ± 0.18 | NA | NA | NA | NA | NA | 0 (0.0) | 101(100.0) | NA | NA |
|  |  | Sapien 3 | 103 | 0.79 ± 0.22 | NA | NA | NA | NA | NA | 0 (0.0) | 103 (100.0) | NA | NA |
| Finkelstein 2018 | OB (PM) | Evolut R | 126 | 0.70 (0.60-0.80) | 43.0 (33.0-53.0) | 55.0 ± 10.0 | NA | NA | NA | 10 (7.9) | 122 (96.8) | 25 (20.0) | 50 (39.5) |
|  |  | Sapien 3 | 126 | 0.70 (0.60-0.80) | 43.0 (33.0-52.0) | 56.0 ± 10.0 | NA | NA | NA | 8 (6.3) | 115 (91.3) | 78 (61.9) | 15 (12.1) |
| Abdelghani 2018 | OB | Evolut R | 100 | 0.54 ± 0.17 * | 39.8 ± 13.9 | 58.8 ± 9.4 | 23.6 ± 1.2 | NA | NA | NA | 100 (100.0) | 25 (25.0) | 39 (39.0) |
|  |  | Sapien 3 | 334 | 0.46 ± 0.16 * | 43.9 ± 6.6 | 55.3 ± 12.4 | 25.2 ± 1.8 | NA | NA | NA | 334 (100.0) | 86 (25.7) | 38 (11.4) |
| Eitan 2018 | OB | Evolut R | 37 | NA | 36.6 ± 11.0 | 50.0 ± 13.0 | 27.4 ± 1.7 | 86.2 ± 6.2 | 583 ± 78 | 35 (94.5) | 37 (100.0) | 30 (81.0) | 12 (32.0) |
|  |  | Sapien 3 | 55 | NA | 39.5 ± 10.0 | 52.0± 12.0 | 27.6 ± 1.6 | 87.3 ± 5.0 | 586 ± 74 | 43 (78.2) | 55 (100.0) | 8 (14.5) | 3 (5.5) |
| Akodad 2018 | OB | Evolut R | 81 | NA | 46.3 ± 14.6 | 52.9 ± 10.7 | NA | NA | NA | 81 (100.0) | 81 (100.0) | NA | NA |
|  |  | Sapien 3 | 147 | NA |  |  | NA | NA | NA | 147 (100.0) | 147 (100.0) | NA | NA |
| Mosleh 2019 | OB | Evolut R/Pro | 129 | 0.73 ± 0.24 | 41.6 ± 12.5 | 55.5 ± 14.7 | 24.0 ± 2.6 | NA | NA | 34 (26.0) | 129 (100.0) | NA | 45 (34.9) |
|  |  | Sapien 3 | 452 | 0.70 ± 0.27 | 42.0 ± 13.9 | 54.8 ± 15.3 | 24.9 ± 2.5 | NA | NA | 91 (20.0) | 452 (100.0) | NA | 113 (25.0) |
| Deharo 2020 | OB(PM) | Evolut R | 10459 | NA | NA | NA | NA | NA | NA | NA | NA | NA | NA |
|  |  | Sapien 3 | 10459 | NA | NA | NA | NA | NA | NA | NA | NA | NA | NA |
| Bisson 2020 | OB | Evolut R | 14446 | NA | NA | NA | NA | NA | NA | NA | NA | NA | NA |
|  |  | Sapien 3 | 25174 | NA | NA | NA | NA | NA | NA | NA | NA | NA | NA |
| Thiele 2020 | RCT | Evolut R | 219 | 0.70 (0.60-0.90) | 38.5 (30.0-50.5) | 12 (5.7) < 35% | NA | NA | NA | 112 (51.1) | 219 (100.0) | NA | NA |
|  |  | Sapien 3 | 219 | 0.80 (0.60-0.90) | 37.0 (26.5-47.5) | 19 (9.1) < 35% | NA | NA | NA | 108 (49.3) | 219 (100.0) | NA | NA |
| Hase 2020 | OB (PM) | Evolut R | 69 | 0.40 (0.35-0.50) * | 48.9 (34.9-69.0) | 64.0 (57.3-68.0) | 21.3 (20.5-22.3) | 67.5 (65.6-70.4) | 353 (327-377) | 39 (56.5) | 69 (100.0) | 40 (58.0) | 11 (15.9) |
|  |  | Sapien 3 | 69 | 0.42 (0.37-0.50) * | 48.0 (39.9-63.8) | 63.0 (53.8-70.0) | 21.2 (20.4-22.1) | 67.5 (65.0-69.9) | 353 (320-375) | 38 (55.1) | 69 (100.0) | 36 (52.2) | 11 (15.9) |
| Armijo 2020 | OB | Evolut R | 193 | 0.78 (0.60-0.90) | 42.0 (32.5-48.0) | 55.0 (41.0-60.0) | 27.9 (27.1-28.8) | 88.6 (86.5-92.0) | 610 (580-649) | 28 (17.3) | 182 (94.3) | 114 (67.5) | 55 (28.5) |
|  |  | Sapien 3 | 640 | 0.76 (0.60-0.90) | 40.2 (30.0-48.0) | 53.0 (39.0-60.0) | 28.2 (27.5-29.0) | 89.3 (87.0-92.2) | 620 (596-660) | 122 (23.6) | 568 (88.8) | 300 (50.8) | 71 (11.1) |
| Okuyama 2020 | OB | Evolut R/Pro | 13 | 0.60 (0.50-0.70) | 29.4 (23.4-48.6) | 67.0 (65.0-71.0) | NA | NA | 297 (280-313) | 13 (100.0) | NA | NA | 1 (8.0) |
|  |  | Sapien 3 | 33 | 0.50 (0.40-0.70) | 43.8 (30.7-61.4) | 69.0 (62.5-73.5) | NA | NA | 309 (303-323) | 33 (100.0) | NA | NA | 3 (9.0) |
| Eftychiou 2020 | OB | Evolut R/Pro | 57 | NA | 41.3 ± 15.5 ^#^ | 55.8 ± 9.8 | 22.6 ± 2.0 | NA | 416 ± 71 | 91 (38.7) ^#^ | 57 (100.0) | 3 (5.3) | 9 (15.7) |
|  |  | Sapien 3 | 178 | NA |  |  |  | NA |  |  | 178 (100.0) | 9 (15.8) | 6 (3.4) |
| Fukuda 2020 | OB | Evolut R | 1550 | NA | NA | NA | NA | NA | NA | NA | 1550 (100.0) | NA | NA |
|  |  | Sapien 3 | 5276 | NA | NA | NA | NA | NA | NA | NA | 5276 (100.0) | NA | NA |
| Habertheuer 2020 | OB | Evolut R/Pro | 269 | NA | 46.0 (41.0-55.0) | 58.0 (50.0-63.0) | NA | NA | NA | 42 (15.6) | 269 (100.0) | NA | NA |
|  |  | Sapien 3 | 294 | NA | 48.0 (41.0-57.0) | 58.0 (47.0-63.0) | NA | NA | NA | 39 (13.3) | 294 (100.0) | NA | NA |
| Tamm 2021 | OB | Evolut R | 144 | 0.71 ± 0.20 | 41.2 ± 17.2 | 54.5 ± 11.3 | NA | NA | NA | 144 (100.0) | 144 (100.0) | 123 (85.4) | 27 (18.8) |
|  |  | Sapien 3 | 215 | 0.80 ± 0.20 | 39.9 ± 16.4 | 49.6 ± 14.4 | NA | NA | NA | 215 (100.0) | 215 (100.0) | 181 (84.2) | 1 (0.5) |
| Lee 2021 | OB | Evolut R/Pro | 45 | NA | 59.4 ± 21.3 | 59.9 ± 15.7 | 22.1 ± 1.0 | 69.9 ± 3.1 | 375 ± 33 | 38 (84.4) | 45 (100.0) | 26 (57.8) | 29 (64.4) |
|  |  | Sapien 3 | 25 | NA | 49.1 ± 11.0 | 67.5 ± 10.4 | 22.4 ± 0.9 | 70.7 ± 2.6 | 387 ± 30 | 16 (64.0) | 25 (100.0) | 19 (76.0) | 2 (8.0) |
| Meguro 2021 | OB | Evolut R | 103 | 0.50 (0.40–0.70) | 47.0 (41.0–60.0) | 69.0 (63.0–77.0) | NA | 60.8 (56.6–62.4) | 280 (270–300) | NA | 97 (94.2) | NA | NA |
|  |  | Sapien 3 | 90 | 0.60 (0.50–0.70) | 49.5 (40.5–66.0) | 68.0 (64.0–74.0) | NA | 61.7 (60.0–63.0) | 290 (270–300) | NA | 86 (95.6) | NA | NA |
| Paitazoglou 2021 | OB | Evolut R | 124 | NA | 39.1 ± 1.3 | 54 ± 0.9 | 23.7 ± 0.3 | NA | NA | NA | 124 (100.0) | 64 (51.6) | 46 (37.1) |
|  |  | Sapien 3 | 129 | NA | 39.8 ± 1.1 | 54.0 ± 1.2 | 24.5 ± 0.2 | NA | NA | NA | 129 (100.0) | 33 (25.6) | 22 (17.1) |
| Kanso 2021 | OB | Evolut R | 49 | 0.74 ± 0.21 | 52.8 ± 13.0 | 59.6 ± 10.3 | 23.1 ± 1.2 | 74.0 ± 3.6 | 422 ± 57 | NA | NA | NA | NA |
|  |  | Sapien 3 | 161 | 0.72 ± 0.19 | 48.6 ± 15.7 | 51.9 ± 12.8 | 24.1 ± 1.4 | 76.0 ± 5.7 | 457 ± 52 | NA | NA | NA | NA |
| Fukui 2021 | OB | Evolut R/Pro | 171 | 0.39 (0.33–0.47) | 38.0 (30.0–46.0) | 60.0 (55.0–65.0) | NA | NA | 450 (406–542) | NA | 153 (92.4) | NA | NA |
|  |  | Sapien 3 | 276 | 0.39 (0.33–0.47) | 40.0 (32.0–48.0) | 60.0 (53.0–65.0) | NA | NA | 481 (406–550) | NA | 266 (96.4) | NA | NA |
| Medranda 2021 | OB | Evolut Pro | 340 | 0.80 ± 0.20 | 40.0 ± 14.0 | 55.4 ± 13.3 | NA | 74.2 ± 6.9 | 441 ± 80 | NA | 340 (100.0) | NA | NA |
|  |  | Sapien 3 | 501 | 0.70 ± 0.20 | 41.4 ± 14.5 | 52.4 ± 14.7 | NA | 78.4 ± 8.7 | 492 ± 104 | NA | 501 (100.0) | NA | NA |
| Minha 2021 | OB | Evolut R/Pro | 276 | NA | NA | NA | NA | NA | NA | NA | 276 (100.0) | 60 (21.9) | 97 (35.4) |
|  |  | Sapien 3 | 121 | NA | NA | NA | NA | NA | NA | NA | 121 (100.0) | 87 (71.9) | 11 (9.2) |
| Catalano 2020 | OB | Evolut R/Pro | 164 | NA | NA | 62.7 | 24.0 | NA | 440 | NA | NA | NA | NA |
|  |  | Sapien 3 | 182 |  |  |  |  |  |  |  |  |  |  |
| Mauri 2017 | OB (PM) | Acurate Neo | 92 | 0.68 ± 0.19 | 46.0 ± 16.0 | 59.0 ± 8.0 | NA | 68.9 ± 2.2 | 361 ± 24 | NA | 92 (100.0) | 87 (94.6) | 41 (44.6) |
|  |  | Sapien 3 | 92 | 0.65 ± 0.17 | 47.0 ± 16.0 | 59.0 ± 10.0 | NA | 68.7 ± 2.9 | 364 ± 29 | NA | 92 (100.0) | 29 (31.5) | 6 (6.5) |
| Schaefer 2017 | OB (PM) | Acurate Neo | 104 | 0.80 ± 0.20 | 35.9 ± 16.6 | 27 (26.0) < 44% | 23.9 ± 2.8 | 77.0 ± 7.9 | 462 ± 97 | 55 (52.9) | 104 (100.0) | 93 (90.3) | 49 (47.6) |
|  |  | Sapien 3 | 104 | 0.80 ± 0.20 | 37.6 ± 16.7 | 23 (22.1) < 44% | 24.8 ± 2.6 | 79.1 ± 9.5 | 487 ± 108 | 68 (65.4) | 104 (100.0) | 56 (53.8) | 21 (20.2) |
| Husser 2017 | OB (PM) | Acurate Neo | 311 | NA | 45.0 ± 15.0 | 18 (5.8) <35% | NA | NA | 440 ± 60 | NA | 311 (100.0) | 298 (95.8) | 131 (42.1) |
|  |  | Sapien 3 | 622 | NA | 44.0 ± 16.0 | 34 (5.5) <35% | NA | NA | 450 ± 80 | NA | 622 (100.0) | 462 (74.3) | 148 (23.8) |
| Pollari 2018 | OB | Acurate | 97 | NA | NA | 53.5 ± 11.9 | NA | 76.5 (70.5-81.4) | 450 (380-500) | 97 (100.0) | 43 (44.0) | 96 (99.0) | 42 (43.3) |
|  |  | Sapien 3 | 206 | NA | NA | 52.5 ± 12.9 | NA | 78.6 (72.8-84.3) | 460 (410-540) | 206 (100.0) | 155 (75.0) | 202 (98.1) | 53 (25.7) |
| Husser 2019 | OB (PM) | Acurate Neo | 65 | 0.70 (0.60–0.90) | 42.0 (34.0–53.0) | 60.0 (50.0–61.0) | 24.4 (23.3–25.5) | 77.3 (73.8–81.2) | 460 (420–510) | 29 (44.6) | 65 (100.0) | 60 (92.3) | 32 (49.2) |
|  |  | Sapien 3 | 65 | 0.70 (0.60–0.90) | 46.0 (36.0–61.0) | 60.0 (50.0–65.0) | 24.6 (22.6–26.1) | 77.4 (72.5–83.1) | 460 (400–530) | 24 (36.9) | 65 (100.0) | 35 (53.8) | 15 (23.1) |
| Lanz 2019 | RCT | Acurate Neo | 372 | 0.70 ± 0.20 | 42.9 ± 17.2 | 56.4 ± 11.1 | NA | 75.7 ± 5.2 | 439 ± 60 | 94 (25.0) | 369 (99.0) | 325 (88.0) | 193 (52.0) |
|  |  | Sapien 3 | 367 | 0.70 ± 0.20 | 41.5 ± 15.1 | 57.1 ± 10.7 | NA | 75.9 ± 5.1 | 443 ± 60 | 84 (23.0) | 363 (99.0) | 83 (23.0) | 45 (13.0) |
| Moriyama 2019 | OB | Acurate Neo | 146 | NA | 43.9 ± 12.7 | 58.5 ± 11.7 | NA | NA | NA | 0 (0.0) | 146 (100.0) | 142 (97.0) | 7 (5.0) |
|  |  | Sapien 3 | 103 | NA | 46.2 ± 14.2 | 56.2 ± 10.3 | NA | NA | NA | 0 (0.0) | 103 (100.0) | 11 (11.0) | 0 (0.0) |
| Barth 2019 | OB (PM) | Acurate | 329 | 0.68 ± 0.18 | 44.0 ± 15.0 | 53.0 ± 13.0 | 21.0 ± 2.0 | 77.3 ± 5.9 | 458 ± 68 | 316 (96.0) | 249 (75.7) | 321 (97.6) | 133 (40.4) |
|  |  | Sapien 3 | 329 | 0.67 ± 0.17 | 45.0 ± 14.0 | 54.0 ± 15.0 | 21.0 ± 3.0 | 77.3 ± 7.9 | 459 ± 93 | 317 (96.4) | 245 (74.5) | 171 (52.1) | 38 (11.6) |
| Kooistra 2020 | OB | Acurate Neo | 78 | NA | NA | 0 (0.0) <30% | NA | NA | NA | 0 (0.0) | 78 (100.0) | 76 (97.4) | 28 (35.9) |
|  |  | Sapien 3 | 230 | NA | NA | 16 (7.0) <30% | NA | NA | NA | 8 (3.5) | 230 (100.0) | 150 (65.2) | 31 (13.5) |
| Mas-Peiro 2019 | OB (PM) | Portico | 104 | NA | 47.0 | 60.0 (15.0-65.0) | NA | NA | NA | 0 (0.0) | 104 (100.0) | NA | 32 (30.8) |
|  |  | Sapien 3 | 73 | NA | 41.0 | 55.0 (15.0-75.0) | NA | NA | NA | 0 (0.0) | 73 (100.0) | NA | 3 (4.1) |
| Wöhrle 2015 | OB | Lotus | 26 | 0.72 ± 0.21 | 41.0 ± 17.0 | NA | 25.2 ± 1.7 | 80.8 ± 4.9 | 499 ± 66 | 0 (0.0) | 26 (100.0) | 9 (35.0) | 0 (0.0) |
|  |  | Sapien 3 | 52 | 0.71 ± 0.17 | 35.0 ± 15.0 | NA | 24.6 ± 1.7 | 79.3 ± 6.5 | 479 ± 78 | 0 (0.0) | 52 (100.0) | 43 (83.0) | 0 (0.0) |
| Rodríguez-Olivares 2016 | OB | Lotus | 35 | NA | NA | NA | NA | NA | NA | 35 (100.0) | NA | NA | NA |
|  |  | Sapien 3 | 26 | NA | NA | NA | NA | NA | NA | 26 (100.0) | NA | NA | NA |
| Soliman 2016 | OB | Lotus | 79 | NA | NA | NA | 24.3 ± 1.7 | 76.9 ± 5.1 | 461 ± 61 | NA | 79 (100.0) | 12 (15.0) | 0 (0.0) |
|  |  | Sapien 3 | 83 | NA | NA | NA | 24.9 ± 2.3 | 79.0 ± 7.2 | 487 ± 89 | NA | 75 (90.0) | 18 (22.0) | 11 (13.0) |
| Pilgrim 2016 | OB | Lotus | 140 | 0.66 ± 0.22 | 49.4 ± 19.5 | 56.1 ± 12.1 | NA | NA | NA | 35 (25.0) | 140 (100) | 43 (31.0) | NA |
|  |  | Sapien 3 | 815 | 0.71 ± 0.23 | 46.1 ± 21.5 | 55.1 ± 14.4 | NA | NA | NA | 314 (38.5) | 815 (100) | 668 (82.0) | NA |
| Vangils 2017 | OB | Lotus | 20 | NA | NA | NA | NA | NA | NA | NA | 20 (100.0) | NA | NA |
|  |  | Sapien 3 | 32 | NA | NA | NA | NA | NA | NA | NA | 25 (78.0) | NA | NA |
| Jarr 2017 | OB | Lotus | 63 | 0.75 ± 0.35 | 38.1 ± 13.0 | NA | 24.7 ± 1.9 | NA | NA | 0 (0.0) | 63 (100.0) | 28 (44.4) | 0 (0.0) |
|  |  | Sapien 3 | 90 | 0.75 ± 0.17 | 38.3 ± 14.5 | NA | 25.4 ± 2.3 | NA | NA | 0 (0.0) | 90 (100.0) | 57 (63.3) | 0 (0.0) |
| Seeger 2017 | OB (PM) | Lotus | 202 | 0.79 ± 0.33 | 36.0 ± 16.0 | 57.0 ± 14.8 | 24.6 ± 2.6 | 79.0 ± 8.3 | 478 ± 101 | 0 (0.0) | 202 (100.0) | 177 (87.6) | 0 (0.0) |
|  |  | Sapien 3 | 202 | 0.78 ± 0.30 | 35.0 ± 15.0 | 57.3 ± 15.0 | 24.3 ± 1.7 | 78.2 ± 5.5 | 466 ± 65 | 0 (0.0) | 202 (100.0) | 189 (93.5) | 0 (0.0) |
| Seeger 2017 ^2^ | OB | Lotus | 100 | 0.82 ± 0.38 ^#^ | 35.2 ± 15.6 ^#^ | 54.4 ± 13.2 ^#^ | 24.6 ± 2.3 ^#^ | 79.5 ± 7.3 ^#^ | 480 ± 89 ^#^ | 0 (0.0) | 100 (100.0) | 176 (88.0) ^#^ | 0 (0.0) |
|  |  | Sapien 3 | 100 |  |  |  |  |  |  | 0 (0.0) | 100 (100.0) |  | 0 (0.0) |
| Schofer 2018 | OB | Lotus | 61 | 0.80 ± 0.30 | 40.6 ± 14.2 | 2 (3.3) < 30% | 24.0 ± 2.0 | 77.6 ± 6.4 | NA | NA | 61 (100.0) | 56 (91.8) | 1 (1.6) |
|  |  | Sapien 3 | 212 | 0.80 ± 0.20 | 35.0 ± 16.8 | 25 (11.8) < 30% | 24.6 ± 2.3 | 86.8 ± 61.6 | NA | NA | 212 (100.0) | 118 (55.7) | 37 (17.5) |
| Naifovino 2018 | OB (PM) | Lotus | 93 | 0.71 ± 0.22 | 47.8 ± 14.1 | 53.2 ± 10.9 | NA | 79.4 ± 9.4 | 483 ± 133 | NA | 93 (100.0) | 47 (50.1) | 0 (0.0) |
|  |  | Sapien 3 | 93 | 0.80 ± 0.21 | 42.6 ± 16.9 | 55.7 ± 12.3 | NA | 79.8 ± 12.6 | 510 ± 129 | NA | 93 (100.0) | 61 (65.3) | 5 (5.3) |
| Sathananthan 2018 | OB | Lotus | 72 | NA | NA | 55.0 ± 12.4 ^#^ | NA | NA | NA | NA | 72 (100.0) | NA | NA |
|  |  | Sapien 3 | 136 | NA | NA |  | NA | NA | NA | NA | 136 (100.0) | NA | NA |
| Schulz 2017 | OB | DMF | 61 | NA | 37.3 ± 1.8 | 46.7 ± 1.6 | 25.2 ± 0.2 | NA | 502 ± 9 | 61 (100.0) | 61 (100.0) | 61 (100.0) | 0 (0.0) |
|  |  | Sapien 3 | 113 | NA | 41.7 ± 1.5 | 47.3 ± 1.2 | 24.5 ± 0.3 | NA | 471 ± 8 | 113 (100.0) | 113 (100.0) | 104 (92.0) | 1 (1.0) |
| Gamal 2019 | OB | DMF | 68 | 0.72 ± 0.19 | 41.5 ± 14.7 | NA | NA | NA | NA | NA | 68 (100.0) | NA | NA |
|  |  | Sapien 3 | 99 | 0.69 ± 0.19 | 44.8 ± 12.5 | NA | NA | NA | NA | NA | 99 (100.0) | NA | NA |
| Edlinge 2021 | OB (PM) | DMF | 42 | NA | 42.0 ± 16.1 | 55.2 ± 10.5 | NA | NA | NA | NA | NA | NA | NA |
|  |  | Sapien 3 | 42 | NA | 40.1 ± 18.8 | 55.7 ± 9.1 | NA | NA | NA | NA | NA | NA | NA |
| Pagnesi 2019 | OB (PM) | Evolut Pro | 251 | 0.68 ± 0.17 | 45.7 ± 16.1 | 56.5 ± 11.6 | NA | NA | NA | NA | 251 (100.0) | 217 (86.5) | 104 (41.4) |
|  |  | Acurate Neo | 251 | 0.71 ± 0.20 | 43.2 ± 16.6 | 56.5 ± 11.7 | NA | NA | NA | NA | 251 (100.0) | 94 (37.9) | 62 (25.0) |
| Tamburino 2020 | RCT | Evolut | 398 | NA | NA | NA | 23.0 (22.0- 24.0) | 73.0 ± 5.0 | 416 ± 56 | 52 (13.0) | 398 (100.0) | 160 (41.0) | 139 (36.0) |
|  |  | Acurate Neo | 398 | NA | NA | NA | 23.0 (22.0- 24.0) | 74.0 ± 5.0 | 429 ± 54 | 52 (13.0) | 398 (100.0) | 306 (79.0) | 177 (46.0) |
| Ivanov 2021 | OB (PM) | Evolut R | 76 | 0.68 (0.60, 0.80) | 40.0 (30.0, 51.5) | 50.0 (38.0, 60.0) | NA | NA | NA | 27 (35.5) | 72 (94.7) | NA | NA |
|  |  | Acurate Neo | 76 | 0.70 (0.50, 0.80) | 42.5 (33.5, 51.0) | 52.0 (40.0, 60.0) | NA | NA | NA | 16 (21.1) | 67 (88.2) | NA | NA |
| Gorla2019 | OB | Evolut R | 263 | NA | 44.8 ± 17.1 | 52.5 ± 10.9 | 23.6 ± 2.6 | 74.8 ± 8.0 | 431 ± 106 | NA | 247 (93.9) | 79 (30.0) | 120 (45.6) |
|  |  | Portico | 111 | NA | 44.2 ± 15.7 | 54.6 ± 10.2 | 23.1 ± 2.0 | 72.7 ± 7.5 | 379 ± 133 | NA | 96 (86.5) | 60 (54.1) | 50 (45.0) |
| Corcione 2020 | OB | Evolut R/Pro | 119 | 0.66 (0.50-0.80) | 50.0 (40.0-61.0) | 55.0 (50.0-60.0) | NA | NA | NA | 1 (1.0) | 119 (100.0) | 42 (35.0) | 43 (36.0) |
|  |  | Portico | 114 | 0.60 (0.50-0.75) | 50.0 (41.0-62.0) | 55.0 (55.0-60.0) | NA | NA | NA | 1 (1.0) | 114 (100.0) | 53 (46.0) | 74 (65.0) |
| Gama 2022 | OB | Acurate | 32 | NA | NA | NA | NA | NA | NA | NA | NA | NA | NA |
|  |  | Portico | 49 | NA | NA | NA | NA | NA | NA | NA | NA | NA | NA |
| Feldman 2018 | RCT | Evolut R | 135 | NA | NA | NA | NA | NA | NA | NA | 135 (100.0) | NA | NA |
|  |  | Lotus | 529 | NA | NA | NA | NA | NA | NA | NA | 529 (100.0) | NA | NA |
| Giannini 2017 | OB | Lotus | 60 | 0.70 ± 0.20 | 48.0 ± 10.0 | 50.0 ± 9.0 | NA | 76.0 ± 14.0 | 453 ± 64 | 0 (0.0) | 60 (100.0) | 40 (66.7) | 0 (0.0) |
|  |  | DFM | 115 | 0.70 ± 0.30 | 50.0 ± 12.0 | 50.0 ± 11.0 | NA | 75.0 ± 13.0 | 456 ± 51 | 0 (0.0) | 115 (100.0) | 115 (100.0) | 0 (0.0) |
| Costa 2019 | OB (PM) | Evolut R | 48 | NA | 52.8 ± 14.1 | 54.8 ± 9.8 | NA | NA | NA | NA | 48 (100.0) | NA | NA |
|  |  | Acurate Neo | 48 | NA | 51.3 ± 14.5 | 54.5 ± 9.7 | NA | NA | NA | NA | 48 (100.0) | NA | NA |
|  |  | Sapien 3 | 48 | NA | 51.3 ± 17.2 | 56.1 ± 9.7 | NA | NA | NA | NA | 48 (100.0) | NA | NA |
| Makkar 2020 | RCT | Evolut R/Pro | 110 | 0.67 ± 0.17 | 46.3 ± 10.8 | 59.3 ± 10.6 | NA | NA | NA | NA | NA | NA | NA |
|  |  | Portico | 375 | 0.68 ± 0.17 | 46.2 ± 11.3 | 57.3 ± 11.3 | NA | NA | NA | NA | NA | NA | NA |
|  |  | Sapien 3 | 206 | 0.68 ± 0.16 | 46.7 ± 11.7 | 57.1 ± 10.8 | NA | NA | NA | NA | NA | NA | NA |
| Tichelba¨cker 2018 | OB | Portico | 16 | NA | NA | NA | NA | NA | NA | NA | 16 (100.0) | NA | NA |
|  |  | DFM | 31 | NA | NA | NA | NA | NA | NA | NA | 31 (100.0) | NA | NA |
|  |  | Sapien 3 | 134 | NA | NA | NA | NA | NA | NA | NA | 134 (100.0) | NA | NA |
| Regazzoli 2019 | OB | Evolut R/Pro | 481 | 0.64 ± 0.01 | 50.8 ± 1.0 | 47 (9.8) <40% | 21.2 ± 0.1 | 67.3 ± 0.2 | 346 ± 3 | NA | 444 (92.3) | 149 (31.0) | 176 (36.6) |
|  |  | Acurate | 201 | 0.64 ± 0.01 | 51.5 ± 1.2 | 16 (7.8) <40% | 21.5 ± 0.1 | 67.3 ± 0.3 | 352 ± 3 | NA | 140 (69.7) | 151 (75.5) | 71 (35.5) |
|  |  | Portico | 177 | 0.65 ± 0.02 | 46.5 ± 1.2 | 8 (4.5) <40% | 21.2 ± 0.1 | 67.4 ± 0.4 | 345 ± 3 | NA | 165 (93.2) | 121 (68.4) | 65 (37.1) |
| Gorla 2020 | OB | Evolut R | 211 | NA | 44.8 ± 16.8 | 53.2 ± 13.1 | 23.9 ± 2.8 | 75.8 ± 8.3 | 450 ± 100 | 0 (0.0) | 202 (95.7) | 51 (24.2) | 79 (37.4) |
|  |  | Acurate Neo | 85 | NA | 47.3 ± 12.9 | 54.7 ± 11.1 | 23.9 ± 2.4 | 75.6 ± 6.8 | 438 ± 78 | 0 (0.0) | 84 (98.8) | 60 (70.6) | 53 (63.4) |
|  |  | Portico | 96 | NA | 43.7 ± 14.0 | 55.7 ± 12.9 | 23.0 ± 2.0 | 73.2 ± 6.0 | 408 ± 66 | 0 (0.0) | 79 (82.3) | 49 (51.0) | 40 (41.7) |
| Vera Vera 2020 | OB (PM) | Evolut R/Pro | 217 | 0.74 ± 0.2 | 44.5 ± 15.0 | 56.0 ± 12.0 | 24.1 ± 3.2 | 76.8 ± 11.7 | 451 ± 113 | NA | 195 (89.9) | 80 (43.0) | 53 (24.9) |
|  |  | Acurate Neo | 107 | 0.72 ± 0.2 | 45.1 ± 14.0 | 59.0 ± 10.0 | 23.3 ± 2.0 | 73.3 ± 10.0 | 417 ± 64 | NA | 102 (95.3) | 92 (87.6) | 26 (24.3) |
|  |  | Portico | 88 | 0.68 ± 0.1 | 45.3 ± 13.0 | 58.0 ± 11.0 | 22.8 ± 1.9 | 69.4 ± 11.0 | 410 ± 68 | NA | 88 (100.0) | 84 (95.5) | 36 (42.4) |
| Bieliauskas 2021 | OB (PM) | Evolut | 20 | 0.80 (0.70-0.90) | 44.0 (37.0-50.0) | 7 (35.0) <50% | NA | NA | NA | NA | 19 (95.0) | 13 (65.0) | 8 (40.0) |
|  |  | Acurate Neo | 20 | 0.70 (0.60-0.80) | 48.0 (40.0-66.0) | 5 (25.0) <50% | NA | NA | NA | NA | 20 (100.0) | 20 (100.0) | 8 (40.0) |
|  |  | Portico | 20 | 0.70 (0.50-0.80) | 50.0 (43.0-66.0) | 5 (25.0) <50% | NA | NA | NA | NA | 19 (95.0) | 19 (95.0) | 11 (55.0) |
| Leone 2021 | OB | Evolut | 248 | NA | NA | NA | NA | NA | NA | NA | NA | NA | NA |
|  |  | Acurate Neo | 104 | NA | NA | NA | NA | NA | NA | NA | NA | NA | NA |
|  |  | Portico | 93 | NA | NA | NA | NA | NA | NA | NA | NA | NA | NA |
| Kim 2017 | OB | Evolut R | 15 | NA | NA | NA | NA | NA | NA | NA | 15 (100.0) | NA | NA |
|  |  | Acurate Neo | 425 | NA | NA | NA | NA | NA | NA | NA | 425 (100.0) | NA | NA |
|  |  | Portico | 127 | NA | NA | NA | NA | NA | NA | NA | 127 (100.0) | NA | NA |
|  |  | Sapien 3 | 381 | NA | NA | NA | NA | NA | NA | NA | 381 (100.0) | NA | NA |
| Mauri 2019 | OB | Evolut R | 132 | NA | NA | NA | NA | 76.8 ± 7.2 | 449 ± 94 | NA | NA | 33 (25.6) | 28 (21.7) |
|  |  | Acurate Neo | 166 | NA | NA | NA | NA | 77.1 ± 7.8 | 455 ± 99 | NA | NA | 151 (92.6) | 41 (25.2) |
|  |  | Lotus | 52 | NA | NA | NA | NA | 77.5 ± 6.6 | 465 ± 80 | NA | NA | 47 (90.4) | 1 (1.9) |
|  |  | Sapien 3 | 292 | NA | NA | NA | NA | 81.6 ± 8.3 | 511 ± 104 | NA | NA | 128 (44.0) | 12 (4.1) |
| Modolo 2020 | OB | Evolut R/Pro | 390 | NA | NA | NA | NA | NA | NA | NA | NA | NA | NA |
|  |  | Acurate | 115 | NA | NA | NA | NA | NA | NA | NA | NA | NA | NA |
|  |  | Lotus | 546 | NA | NA | NA | NA | NA | NA | NA | NA | NA | NA |
|  |  | Sapien 3 | 397 | NA | NA | NA | NA | NA | NA | NA | NA | NA | NA |
| Stundl 2019 | OB | Evolut R | 114 | 0.72 ± 0.18 | 44.1 ± 18.5 | 56.1 ± 12.0 | 23.1 ± 2.1 | NA | NA | NA | 114 (100.0) | 34 (29.8) | 37 (32.5) |
|  |  | Lotus | 104 | 0.73 ± 0.14 | 43.0 ± 14.5 | 57.7 ± 10.6 | 23.9 ± 1.7 | NA | NA | NA | 104 (100.0) | 12 (11.5) | 4 (3.8) |
|  |  | DFM | 38 | 0.75 ± 0.19 | 38.8 ± 14.5 | 51.5 ± 11.9 | 25.0 ± 2.8 | NA | NA | NA | 38 (100.0) | 38 (100.0) | 1 (2.6) |
|  |  | Sapien 3 | 101 | 0.74 ± 0.15 | 41.1 ± 13.6 | 57.4 ± 12.2 | 25.5 ± 2.4 | NA | NA | NA | 101 (100.0) | 68 (68.0) | 3 (3.0) |
| Panoulas 2020 | OB | Evolut R | 22 | NA | NA | NA | NA | NA | NA | NA | NA | NA | NA |
|  |  | Lotus | 25 | NA | NA | NA | NA | NA | NA | NA | NA | NA | NA |
|  |  | DFM | 25 | NA | NA | NA | NA | NA | NA | NA | NA | NA | NA |
|  |  | Sapien 3 | 48 | NA | NA | NA | NA | NA | NA | NA | NA | NA | NA |
| Nicolas 2021 | OB | Evolut R | 70 | NA | NA | NA | NA | NA | NA | NA | NA | NA | NA |
|  |  | Lotus | 54 | NA | NA | NA | NA | NA | NA | NA | NA | NA | NA |
|  |  | DFM | 29 | NA | NA | NA | NA | NA | NA | NA | NA | NA | NA |
|  |  | Sapien 3 | 209 | NA | NA | NA | NA | NA | NA | NA | NA | NA | NA |
| Santos-Martinez 2022 | OB | Evolut R | 298 | 0.74 ± 0.24 | 46.6 ± 16.7 | 56.4 ± 12.4 | 24.3 ± 3.1 | 77.3 ± 11.0 | 445 ± 136 | NA | 266 (89.3) | 128 (48.6) | 70 (24.4) |
|  |  | Acurate | 180 | 0.73 ± 0.16 | 45.9 ± 13.5 | 58.5 ± 11.3 | 23.7 ± 2.0 | 74.8 ± 10.9 | 424 ± 88 | NA | 170 (94.4) | 158 (89.8) | 33 (19.0) |
|  |  | Portico | 125 | 0.68 ± 0.18 | 44.8 ± 14.0 | 57.7 ± 12.1 | 23.1 ± 2.2 | 72.0 ± 14.4 | 415 ± 82 | NA | 123 (98.4) | 119 (95.2) | 43 (35.8) |
|  |  | Sapien 3 | 290 | 0.70 ± 0.17 | 45.3 ± 14.7 | 57.3 ± 11.9 | 24.8 ± 2.8 | 72.9 ± 17.5 | 476 ± 95 | NA | 264 (91.0) | 93 (32.1) | 21 (7.3) |
| Marzahn 2017 | OB | Portico | 26 | NA | NA | NA | NA | NA | NA | 26 (100.0) | 26 (100.0) | NA | NA |
|  |  | Lotus | 14 | NA | NA | NA | NA | NA | NA | 14 (100.0) | 14 (100.0) | NA | NA |
|  |  | DFM | 41 | NA | NA | NA | NA | NA | NA | 41 (100.0) | 41 (100.0) | NA | NA |
|  |  | Sapien 3 | 234 | NA | NA | NA | NA | NA | NA | 234 (100.0) | 234 (100.0) | NA | NA |
| Giordano 2019 | OB | Evolut | 703 | 0.67 ± 0.26 | 47.3 ± 14.9 | 52.0 ± 10.0 | NA | NA | NA | 109 (15.5) | 612 (87.1) | 431 (61.3) | 202 (28.7) |
|  |  | Acurate | 234 | 0.67 ± 0.23 | 47.8 ± 13.8 | 53.0 ± 11.0 | NA | NA | NA | 32 (13.7) | 216 (92.3) | 152 (65.0) | 111 (47.4) |
|  |  | Portico | 347 | 0.69 ± 0.24 | 48.0 ± 16.8 | 54.0 ± 10.0 | NA | NA | NA | 34 (9.8) | 303 (87.3) | 230 (66.3) | 165 (47.6) |
|  |  | Lotus | 151 | 0.66 ± 0.24 | 48.0 ± 14.8 | 53.0 ± 12.0 | NA | NA | NA | 17 (11.3) | 141 (93.4) | 58 (38.4) | 2 (1.3) |
|  |  | Sapien 3 | 541 | 0.63 ± 0.18 | 48.2 ± 13.8 | 53.0 ± 10.0 | NA | NA | NA | 121 (22.4) | 510 (94.3) | 449 (83.0) | 27 (5.0) |
| Okuno 2020 | OB | Evolut | 237 | NA | NA | NA | NA | NA | NA | 184 (18.1) | 931 (91.5) | 654 (64.2) | 294 (28.9) |
|  |  | Acurate | 170 | NA | NA | NA | NA | NA | NA |  |  |  |  |
|  |  | Portico | 23 | NA | NA | NA | NA | NA | NA |  |  |  |  |
|  |  | Lotus | 120 | NA | NA | NA | NA | NA | NA |  |  |  |  |
|  |  | Sapien 3 | 468 | NA | NA | NA | NA | NA | NA |  |  |  |  |
| Voigtlände 2021 | OB | Evolut | 179 | NA | 40.0 (32.0–48.0) | 133 (74.7) < 30 | NA | 68.4 (64.9–70.1) | 360 (321-380) | 26 (14.5) | 179 (100.0) | 45 (25.1) | 46 (25.7) |
|  |  | Acurate | 428 | NA | 42.0 (32.0–54.0) | 294 (68.9) < 30 | NA | 69.5 (67.2–71.0) | 368 (345-383) | 73 (17.1) | 428 (100.0) | 294 (69.2) | 134 (31.5) |
|  |  | Portico | 110 | NA | 44.0 (32.0–56.0) | 74 (67.3) < 30 | NA | 68.8 (66.6–70.7) | 362 (332-381) | 33 (30.0) | 110 (100.0) | 86 (78.2) | 46 (41.8) |
|  |  | Lotus | 64 | NA | 44.0 (33.1–57.0) | 27 (42.2) < 30 | NA | 70.0 (68.5–71.3) | 370 (354-388) | 9 (14.0) | 64 (100.0) | 32 (50.8) | 3 (4.8) |
|  |  | Sapien 3 | 288 | NA | 42.0 (31.8–53.0) | 180 (62.7) <30 | NA | 70.0 (68.0–71.2) | 376 (352-386) | 99 (34.3) | 288 (100.0) | 113 (39.2) | 37 (12.8) |

Continuous variables are presented as mean value ± standard deviation or median and interquartile range (IQR), categorical variables are presented as n (%). Abbreviations: OB: observational study; PM: propensity score matched study; PN: patient number; AVA: aortic valve area; MAVG: mean aortic valve gradient; LVEF: left ventricular ejection fraction; MDCT: multidetector computed tomography; NA: not available; *: indexed AVA;

**Table 4. Quality assessment of observational studies**

| **Study** | **1** | **2** | **3** | **4** | **5** | **6** | **7** | **8** | **Total score** | **Risk of bias** |
| --- | --- | --- | --- | --- | --- | --- | --- | --- | --- | --- |
| Ben-shoshan 2016 | ★ | ★ | ★ |  | ★ | ★ | ★ | ★ | 7★ | moderate |
| Enríquez-Rodríguez 2017 | ★ | ★ | ★ | ★ | ★★ | ★ | ★ | ★ | 9★ | low |
| Rogers 2017 | ★ | ★ | ★ | ★ | ★★ | ★ | ★ | ★ | 9★ | low |
| Vlastra 2018 | ★ | ★ | ★ |  | ★★ | ★ | ★ | ★ | 8★ | low |
| Veulemans 2018 | ★ | ★ | ★ |  | ★★ | ★ | ★ | ★ | 8★ | low |
| Finkelstein2018 | ★ | ★ | ★ |  | ★★ | ★ | ★ | ★ | 8★ | low |
| Abdelghani 2018 | ★ | ★ | ★ | ★ | ★ | ★ | ★ | ★ | 8★ | low |
| Eitan 2018 | ★ | ★ | ★ | ★ | ★★ | ★ | ★ | ★ | 9★ | low |
| Akodad 2018 | ★ | ★ | ★ | ★ |  | ★ | ★ | ★ | 7★ | moderate |
| Mosleh 2019 | ★ | ★ | ★ |  | ★ | ★ | ★ | ★ | 7★ | moderate |
| Deharo 2020 | ★ | ★ | ★ |  | ★★ | ★ | ★ | ★ | 8★ | low |
| Bisson 2020 | ★ | ★ | ★ |  | ★ | ★ | ★ | ★ | 7★ | moderate |
| Hase 2020 | ★ | ★ | ★ | ★ | ★★ | ★ | ★ | ★ | 9★ | low |
| Armijo 2020 | ★ | ★ | ★ | ★ | ★ | ★ | ★ | ★ | 8★ | low |
| Okuyama 2020 | ★ | ★ | ★ |  | ★★ | ★ | ★ | ★ | 8★ | low |
| Eftychiou2020 | ★ | ★ | ★ | ★ |  | ★ | ★ | ★ | 7★ | moderate |
| Fukuda 2020 | ★ | ★ | ★ |  |  | ★ | ★ | ★ | 6★ | moderate |
| Habertheuer 2020 | ★ | ★ | ★ | ★ | ★ | ★ | ★ | ★ | 8★ | low |
| Tamm 2021 | ★ | ★ | ★ |  | ★ | ★ | ★ | ★ | 7★ | moderate |
| Lee 2021 | ★ | ★ | ★ |  | ★ | ★ | ★ | ★ | 7★ | moderate |
| Meguro 2021 | ★ | ★ | ★ |  | ★★ | ★ | ★ | ★ | 8★ | low |
| Paitazoglou 2021 | ★ | ★ | ★ |  | ★ | ★ | ★ | ★ | 7★ | moderate |
| Kanso 2021 | ★ | ★ | ★ |  | ★ | ★ | ★ | ★ | 7★ | moderate |
| Fukui 2021 | ★ | ★ | ★ | ★ | ★ | ★ | ★ | ★ | 8★ | low |
| Medranda 2021 | ★ | ★ | ★ |  | ★★ | ★ | ★ | ★ | 8★ | low |
| Minha 2021 | ★ | ★ | ★ |  | ★ | ★ | ★ | ★ | 7★ | moderate |
| Catalano 2020 | ★ | ★ | ★ |  |  | ★ | ★ | ★ | 6★ | moderate |
| Edlinge 2021 | ★ | ★ | ★ |  | ★★ | ★ | ★ | ★ | 8★ | low |
| Gama 2022 | ★ | ★ | ★ |  |  | ★ | ★ | ★ | 6★ | moderate |
| Mauri 2017 | ★ | ★ | ★ |  | ★★ | ★ | ★ | ★ | 8★ | low |
| Schaefer 2017 | ★ | ★ | ★ |  | ★★ | ★ | ★ | ★ | 8★ | low |
| Husser 2017 | ★ | ★ | ★ | ★ | ★★ | ★ | ★ | ★ | 9★ | low |
| Pollari 2018 | ★ | ★ | ★ | ★ | ★ | ★ | ★ | ★ | 8★ | low |
| Husser 2019 | ★ | ★ | ★ | ★ | ★★ | ★ | ★ | ★ | 9★ | low |
| Moriyama 2019 | ★ | ★ | ★ |  | ★★ | ★ | ★ | ★ | 8★ | low |
| Barth 2019 | ★ | ★ | ★ |  | ★★ | ★ | ★ | ★ | 8★ | low |
| Kooistra 2020 | ★ | ★ | ★ |  | ★ | ★ | ★ | ★ | 7★ | moderate |
| Mas-Peiro 2019 | ★ | ★ | ★ | ★ | ★ | ★ | ★ | ★ | 8★ | low |
| Wöhrle 2015 | ★ | ★ | ★ |  | ★★ | ★ | ★ | ★ | 8★ | low |
| Rodríguez-Olivares 2016 | ★ | ★ | ★ | ★ |  | ★ | ★ | ★ | 7★ | moderate |
| Soliman 2016 | ★ | ★ | ★ | ★ | ★ | ★ | ★ | ★ | 8★ | low |
| Pilgrim 2016 | ★ | ★ | ★ | ★ | ★ | ★ | ★ | ★ | 8★ | low |
| Vangils 2017 | ★ | ★ | ★ |  | ★ | ★ | ★ | ★ | 7★ | moderate |
| Jarr 2017 | ★ | ★ | ★ |  | ★ | ★ | ★ | ★ | 7★ | moderate |
| Seeger 2017 | ★ | ★ | ★ | ★ | ★★ | ★ | ★ | ★ | 9★ | low |
| Seeger 2017 ^2^ | ★ | ★ | ★ | ★ | ★ | ★ | ★ | ★ | 8★ | low |
| Schofer 2018 | ★ | ★ | ★ |  | ★ | ★ | ★ | ★ | 7★ | moderate |
| Naifovino 2018 | ★ | ★ | ★ | ★ | ★★ | ★ | ★ | ★ | 9★ | low |
| Sathananthan 2018 | ★ | ★ | ★ | ★ |  | ★ | ★ | ★ | 7★ | moderate |
| Schulz 2017 | ★ | ★ | ★ |  | ★ | ★ | ★ | ★ | 7★ | moderate |
| Gamal2019 | ★ | ★ | ★ |  | ★ | ★ | ★ | ★ | 7★ | moderate |
| Pagnesi 2019 | ★ | ★ | ★ |  | ★★ | ★ | ★ | ★ | 8★ | low |
| Ivanov 2021 | ★ | ★ | ★ |  | ★★ | ★ | ★ | ★ | 8★ | low |
| Gorla2019 | ★ | ★ | ★ |  | ★ | ★ | ★ | ★ | 7★ | moderate |
| Corcione 2020 | ★ | ★ | ★ | ★ | ★★ | ★ | ★ | ★ | 9★ | low |
| Giannini2017 | ★ | ★ | ★ |  | ★★ | ★ | ★ | ★ | 8★ | low |
| Costa 2019 | ★ | ★ | ★ |  | ★★ | ★ | ★ | ★ | 8★ | low |
| Tichelba¨cker 2018 | ★ | ★ | ★ | ★ |  | ★ | ★ | ★ | 7★ | moderate |
| Regazzoli 2019 | ★ | ★ | ★ |  | ★ | ★ | ★ | ★ | 7★ | moderate |
| Gorla 2020 | ★ | ★ | ★ |  | ★ | ★ | ★ | ★ | 7★ | moderate |
| Vera Vera 2020 | ★ | ★ | ★ |  | ★ | ★ | ★ | ★ | 7★ | moderate |
| Bieliauskas 2021 | ★ | ★ | ★ | ★ |  | ★ | ★ | ★ | 7★ | moderate |
| Leone 2021 | ★ | ★ | ★ | ★ |  | ★ | ★ | ★ | 7★ | moderate |
| Kim 2017 | ★ | ★ | ★ |  |  | ★ | ★ | ★ | 6★ | moderate |
| Mauri 2019 | ★ | ★ | ★ |  |  | ★ | ★ | ★ | 6★ | moderate |
| Modolo 2020 | ★ | ★ | ★ |  |  | ★ | ★ | ★ | 6★ | moderate |
| Stundl 2019 | ★ | ★ | ★ |  | ★ | ★ | ★ | ★ | 7★ | moderate |
| Panoulas 2020 | ★ | ★ | ★ |  |  | ★ | ★ | ★ | 6★ | moderate |
| Nicolas 2021 | ★ | ★ | ★ |  |  | ★ | ★ | ★ | 6★ | moderate |
| Santos-Martinez 2022 | ★ | ★ | ★ |  |  | ★ | ★ | ★ | 6★ | moderate |
| Marzahn 2017 | ★ | ★ | ★ |  |  | ★ | ★ | ★ | 6★ | moderate |
| Giordano 2019 | ★ | ★ | ★ | ★ |  | ★ | ★ | ★ | 7★ | moderate |
| Okuno 2020 | ★ | ★ | ★ |  |  | ★ | ★ | ★ | 6★ | moderate |
| Voigtlände 2021 | ★ | ★ | ★ |  | ★ | ★ | ★ | ★ | 7★ | moderate |

1. representativeness of the exposed cohort

2. selection of the nonexposed cohort

3. ascertainment of exposure

4. outcome of interest was not present at start of study

5. comparability of cohorts

6. assessment of outcome

7. long enough follow up:

8. adequacy of follow up

Total score ≥ 8 ★: low risk of bias; 6-7 ★: moderate risk of bias; ≤5 ★: high risk of bias

**Table 5. Quality assessment of RCTs**

| **Study** | **1** | **2** | **3** | **4** | **5** | **6** | **7** | **Total score** | **Risk of bias** |
| --- | --- | --- | --- | --- | --- | --- | --- | --- | --- |
| Thiele 2020 | A | A | B | A | A | A | A | 6A | low |
| Lanz 2019 | A | C | B | A | A | A | A | 5A | moderate |
| Makkar 2020 | A | C | B | A | A | A | B | 4A | moderate |
| Tamburino 2020 | A | C | B | A | A | A | B | 4A | moderate |
| Feldman 2018 | A | C | B | A | A | A | A | 5A | moderate |

1. random sequence generation

2. allocation concealment

3. blinding of participants and personnel

4. blinding of outcome assessment

5. incomplete outcome data

6. selective reporting

7. other bias

A: low risk; B: high risk; C: unclear risk

Total score ≥ 6 A: low risk of bias; 4-5 A: moderate risk of bias; ≤ 3A: high risk of bias

**Table 6. Surface under the cumulative rankings (SUCRAs) and the mean ranks for each endpoint**

1. **Device success** **(B) Mortality**  **(C) Stroke (D) Major/life threatening bleeding**

| **Treatment** | **SUCRA** | **PrBest** | **MeanRank** |  | **Treatment** | **SUCRA** | **PrBest** | **MeanRank** |  | **Treatment** | **SUCRA** | **PrBest** | **MeanRank** |  | **Treatment** | **SUCRA** | **PrBest** | **MeanRank** |
| --- | --- | --- | --- | --- | --- | --- | --- | --- | --- | --- | --- | --- | --- | --- | --- | --- | --- | --- |
| Evolut | 51.8 | 8.0 | 3.4 |  | Evolut | 60.7 | 5.4 | 3.0 |  | Evolut | 76.7 | 12.7 | 2.2 |  | Evolut | 27.6 | 1.1 | 4.6 |
| Acurate | 61.3 | 16.8 | 2.9 |  | Acurate | 52.0 | 6.4 | 3.4 |  | Acurate | 36.5 | 0.1 | 4.2 |  | Acurate | 50.6 | 8.5 | 3.5 |
| Portico | 58.3 | 24.9 | 3.1 |  | Portico | 65.7 | 21.3 | 2.7 |  | Portico | 58.7 | 7.9 | 3.1 |  | Portico | 42.7 | 9.7 | 3.9 |
| Lotus | 48.8 | 18.7 | 3.6 |  | Lotus | 85.1 | 59.2 | 1.7 |  | Lotus | 94.1 | 77.9 | 1.3 |  | Lotus | 76.3 | 43.3 | 2.2 |
| DFM | 4.6 | 1.5 | 5.8 |  | DFM | 19.7 | 7.7 | 5.0 |  | DFM | 8.6 | 1.3 | 5.6 |  | DFM | 35.8 | 23.5 | 4.2 |
| Sapien3 | 75.3 | 30.1 | 2.2 |  | Sapien3 | 16.8 | 0.0 | 5.2 |  | Sapien3 | 25.5 | 0.0 | 4.7 |  | Sapien3 | 67.1 | 13.9 | 2.6 |
| **(E) Major vascular complications** | | | |  | **(F) Acute kidney injury** | | | |  | **(G) Permanent pacemaker implantation** | | | |  | **(H) Procedural mortality** | | | |
| **Treatment** | **SUCRA** | **PrBest** | **MeanRank** |  | **Treatment** | **SUCRA** | **PrBest** | **MeanRank** |  | **Treatment** | **SUCRA** | **PrBest** | **MeanRank** |  | **Treatment** | **SUCRA** | **PrBest** | **MeanRank** |
| Evolut | 51.3 | 8.4 | 3.4 |  | Evolut | 46.5 | 2.4 | 3.7 |  | Evolut | 70.3 | 0.0 | 2.5 |  | Evolut | 50.4 | 3.9 | 3.5 |
| Acurate | 79.6 | 45.1 | 2.0 |  | Acurate | 50.1 | 4.8 | 3.5 |  | Acurate | 8.6 | 0.0 | 5.6 |  | Acurate | 70.0 | 22.2 | 2.5 |
| Portico | 44.8 | 11.1 | 3.8 |  | Portico | 22.6 | 1.8 | 4.9 |  | Portico | 69.6 | 0.0 | 2.5 |  | Portico | 7.7 | 0.7 | 5.6 |
| Lotus | 40.8 | 10.0 | 4.0 |  | Lotus | 47.0 | 4.3 | 3.7 |  | Lotus | 100 | 100.0 | 1.0 |  | Lotus | 80.3 | 40.4 | 2.0 |
| DFM | 22.4 | 14.2 | 4.9 |  | DFM | 92.1 | 85.2 | 1.4 |  | DFM | 13.2 | 0.0 | 5.3 |  | DFM | 57.0 | 32.7 | 3.2 |
| Sapien3 | 61.1 | 11.2 | 2.9 |  | Sapien3 | 41.9 | 1.4 | 3.9 |  | Sapien3 | 38.3 | 0.0 | 4.1 |  | Sapien3 | 34.5 | 0.2 | 4.3 |
| **(I) No correct positioning** | | | |  | **(J) Moderate-to-severe paravalvular leak** | | | |  | **(K) Prosthesis patient mismatch** | | | |  | **(L) Mean aortic valve gradients** | | | |
| **Treatment** | **SUCRA** | **PrBest** | **MeanRank** |  | **Treatment** | **SUCRA** | **PrBest** | **MeanRank** |  | **Treatment** | **SUCRA** | **PrBest** | **MeanRank** |  | **Treatment** | **SUCRA** | **PrBest** | **MeanRank** |
| Evolut | 84.6 | 38.2 | 1.8 |  | Evolut | 52.2 | 0.0 | 3.4 |  | Evolut | 4.7 | 0.0 | 5.8 |  | Evolut | 1.5 | 0.0 | 5.9 |
| Acurate | 52.6 | 1.4 | 3.4 |  | Acurate | 76.9 | 7.4 | 2.2 |  | Acurate | 23.5 | 0.0 | 4.8 |  | Acurate | 35.5 | 0.0 | 4.2 |
| Portico | 85.0 | 47.2 | 1.8 |  | Portico | 96.2 | 82.9 | 1.2 |  | Portico | 43.8 | 0.2 | 3.8 |  | Portico | 23.0 | 0.0 | 4.8 |
| Lotus | 27.7 | 2.5 | 4.6 |  | Lotus | 0.3 | 0.0 | 6.0 |  | Lotus | 70.6 | 9.8 | 2.5 |  | Lotus | 79.8 | 0.0 | 2.0 |
| DFM | 34.9 | 10.7 | 4.3 |  | DFM | 51.7 | 9.7 | 3.4 |  | DFM | 67.0 | 32.6 | 2.7 |  | DFM | 100.0 | 100.0 | 1.0 |
| Sapien3 | 15.3 | 0.0 | 5.2 |  | Sapien3 | 22.7 | 0.0 | 4.9 |  | Sapien3 | 90.3 | 57.4 | 1.5 |  | Sapien3 | 60.2 | 0.0 | 3.0 |
|  | | | |  |  | | | |  |  | | | |  |  | | | |

**Table 7.** **Network heterogeneity for each endpoint**

| **Endpoint** | **Heterogeneity** |
| --- | --- |
| Device success | 0.334 |
| Mortality | 0.000 |
| Stroke | 0.081 |
| Major/life threatening bleeding | 0.288 |
| Major vascular complications | 0.360 |
| Acute kidney injury | 0.171 |
| Permanent pacemaker implantation | 0.242 |
| Procedural mortality | 0.000 |
| No correct position | 0.355 |
| Moderate-to-severe paravalvular leak | 0.315 |
| Prosthesis patient mismatch | 0.258 |
| Mean aortic valve gradients | 1.077 |

**Table 8.** **Design-by-treatment test for each endpoint**

| **Endpoint** | **Chi-square** | **Global inconsistency P-value** |
| --- | --- | --- |
| Device success | chi2 (14) = 10.02 | Prob > chi2 = 0.7607 |
| Mortality | chi2 (22) = 24.89 | Prob > chi2 = 0.3023 |
| Stroke | chi2 (22) = 14.60 | Prob > chi2 = 0.8789 |
| Major/life threatening bleeding | chi2 (22) = 43.25 | Prob > chi2 = 0.0044 |
| Major vascular complications | chi2 (21) = 30.63 | Prob > chi2 = 0.0800 |
| Acute kidney injury | chi2 (17) = 15.53 | Prob > chi2 = 0.5572 |
| Permanent pacemaker implantation | chi2 (28) = 41.93 | Prob > chi2 = 0.0441 |
| Procedural mortality | chi2 (10) = 11.50 | Prob > chi2 = 0.3201 |
| No correct position | chi2 (12) = 12.12 | Prob > chi2 = 0.4364 |
| Moderate-to-severe paravalvular leak | chi2 (20) = 16.40 | Prob > chi2 = 0.6916 |
| Prosthesis patient mismatch | chi2 (8) = 24.12 | Prob > chi2 = 0.0022 |
| Mean aortic valve gradients | chi2 (16) = 5.77 | Prob > chi2 = 0.9904 |

**Table 9. Loop-specific heterogeneity estimates for each endpoint**

**(A) Device success (B) Mortality**

| **Loop** | **IF** | **seIF** | **z_value** | **p_value** | **CI_95** | **Loop_Heterog_tau2** |  | **Loop** | **IF** | **seIF** | **z_value** | **p_value** | **CI_95** | **Loop_Heterog_tau2** |
| --- | --- | --- | --- | --- | --- | --- | --- | --- | --- | --- | --- | --- | --- | --- |
| Lotus-DFM-Sapien3 | 2.634 | 1.055 | 2.497 | 0.013 | (0.57,4.70) | 0.000 |  | Acurate-Lotus-Sapien3 | 0.922 | 0.511 | 1.804 | 0.071 | (0.00,1.92) | 0.000 |
| Acurate-Lotus-Sapien3 | 0.918 | 1.073 | 0.855 | 0.392 | (0.00,3.02) | 0.192 |  | Acurate-Portico-Lotus | 0.854 | 0.739 | 1.155 | 0.248 | (0.00,2.30) | 0.000 |
| Evolut-Acurate-Lotus | 0.734 | 1.14 | 0.644 | 0.520 | (0.00,2.97) | 0.000 |  | Evolut-Acurate-Lotus | 0.666 | 0.597 | 1.116 | 0.264 | (0.00,1.84) | 0.000 |
| Portico-Lotus-Sapien3 | 0.722 | 0.822 | 0.878 | 0.380 | (0.00,2.33) | 0.000 |  | Evolut-Acurate-Portico | 0.614 | 0.490 | 1.254 | 0.210 | (0.00,1.57) | 0.000 |
| Evolut-Lotus-Sapien3 | 0.610 | 0.744 | 0.821 | 0.412 | (0.00,2.07) | 0.048 |  | Evolut-Portico-Lotus | 0.538 | 0.654 | 0.822 | 0.411 | (0.00,1.82) | 0.000 |
| Evolut-Acurate-Portico | 0.505 | 0.318 | 1.588 | 0.112 | (0.00,1.13) | 0.000 |  | Lotus-DFM-Sapien3 | 0.380 | 1.047 | 0.363 | 0.717 | (0.00,2.43) | 0.000 |
| Acurate-Portico-Lotus | 0.200 | 1.219 | 0.164 | 0.870 | (0.00,2.59) | 0.000 |  | Evolut-Lotus-Sapien3 | 0.353 | 0.410 | 0.860 | 0.390 | (0.00,1.16) | 0.000 |
| Evolut-Portico-Lotus | 0.152 | 1.039 | 0.146 | 0.884 | (0.00,2.19) | 0.000 |  | Acurate-Portico-Sapien3 | 0.206 | 0.449 | 0.459 | 0.646 | (0.00,1.09) | 0.000 |
| Acurate-Portico-Sapien3 | 0.088 | 0.550 | 0.161 | 0.872 | (0.00,1.17) | 0.233 |  | Portico-Lotus-Sapien3 | 0.159 | 0.618 | 0.257 | 0.798 | (0.00,1.37) | 0.015 |
| Evolut-Acurate-Sapien3 | 0.073 | 0.348 | 0.210 | 0.834 | (0.00,0.76) | 0.182 |  | Evolut-DFM-Sapien3 | 0.131 | 1.685 | 0.078 | 0.938 | (0.00,3.43) | 0.000 |
| Evolut-Portico-Sapien3 | 0.015 | 0.393 | 0.038 | 0.969 | (0.00,0.79) | 0.060 |  | Evolut-Portico-Sapien3 | 0.081 | 0.351 | 0.231 | 0.817 | (0.00,0.77) | 0.000 |
|  |  |  |  |  |  |  |  | Evolut-Lotus-DFM | 0.040 | 1.789 | 0.022 | 0.982 | (0.00,3.55) | 0.000 |
|  |  |  |  |  |  |  |  | Evolut-Acurate-Sapien3 | 0.032 | 0.275 | 0.116 | 0.908 | (0.00,0.57) | 0.000 |

**(C) Stroke (D) Major/life threatening bleeding**

| **Loop** | **IF** | **seIF** | **z_value** | **p_value** | **CI_95** | **Loop_Heterog_tau2** |  | **Loop** | **IF** | **seIF** | **z_value** | **p_value** | **CI_95** | **Loop_Heterog_tau2** |
| --- | --- | --- | --- | --- | --- | --- | --- | --- | --- | --- | --- | --- | --- | --- |
| Evolut-DFM-Sapien3 | 2.699 | 2.221 | 1.215 | 0.224 | (0.00,7.05) | 0.046 |  | Evolut-DFM-Sapien3 | 1.742 | 2.166 | 0.804 | 0.421 | (0.00,5.99) | 0.011 |
| Evolut-Lotus-DFM | 1.795 | 2.201 | 0.816 | 0.415 | (0.00,6.11) | 0.000 |  | Lotus-DFM-Sapien3 | 1.14 | 1.108 | 1.029 | 0.304 | (0.00,3.31) | 0.000 |
| Evolut-Portico-Lotus | 1.274 | 0.934 | 1.364 | 0.172 | (0.00,3.10) | 0.000 |  | Evolut-Acurate-Lotus | 0.832 | 0.669 | 1.244 | 0.214 | (0.00,2.14) | 0.248 |
| Evolut-Portico-Sapien3 | 0.756 | 0.535 | 1.412 | 0.158 | (0.00,1.80) | 0.074 |  | Evolut-Lotus-Sapien3 | 0.804 | 0.384 | 2.092 | 0.036 | (0.05,1.56) | 0.000 |
| Portico-Lotus-Sapien3 | 0.688 | 0.850 | 0.810 | 0.418 | (0.00,2.35) | 0.000 |  | Evolut-Portico-Lotus | 0.768 | 0.543 | 1.416 | 0.157 | (0.00,1.83) | 0.000 |
| Acurate-Lotus-Sapien3 | 0.619 | 0.666 | 0.930 | 0.352 | (0.00,1.92) | 0.000 |  | Acurate-Lotus-Sapien3 | 0.644 | 0.372 | 1.733 | 0.083 | (0.00,1.37) | 0.000 |
| Evolut-Acurate-Lotus | 0.586 | 0.755 | 0.776 | 0.438 | (0.00,2.07) | 0.000 |  | Portico-Lotus-Sapien3 | 0.513 | 0.464 | 1.104 | 0.270 | (0.00,1.42) | 0.000 |
| Lotus-DFM-Sapien3 | 0.573 | 1.216 | 0.471 | 0.637 | (0.00,2.96) | 0.000 |  | Evolut-Portico-Sapien3 | 0.456 | 0.314 | 1.453 | 0.146 | (0.00,1.07) | 0.011 |
| Evolut-Lotus-Sapien3 | 0.557 | 0.501 | 1.111 | 0.267 | (0.00,1.54) | 0.000 |  | Evolut-Acurate-Sapien3 | 0.373 | 0.277 | 1.349 | 0.177 | (0.00,0.92) | 0.04 |
| Evolut-Acurate-Sapien3 | 0.407 | 0.362 | 1.122 | 0.262 | (0.00,1.12) | 0.010 |  | Acurate-Portico-Lotus | 0.273 | 0.619 | 0.441 | 0.659 | (0.00,1.49) | 0.000 |
| Evolut-Acurate-Portico | 0.387 | 0.602 | 0.642 | 0.521 | (0.00,1.57) | 0.000 |  | Evolut-Acurate-Portico | 0.239 | 0.447 | 0.534 | 0.594 | (0.00,1.11) | 0.054 |
| Acurate-Portico-Lotus | 0.178 | 1.058 | 0.168 | 0.866 | (0.00,2.25) | 0.000 |  | Acurate-Portico-Sapien3 | 0.127 | 0.356 | 0.358 | 0.720 | (0.00,0.83) | 0.027 |
| Acurate-Portico-Sapien3 | 0.055 | 0.583 | 0.095 | 0.925 | (0.00,1.20) | 0.000 |  | Evolut-Lotus-DFM | 0.060 | 2.171 | 0.028 | 0.978 | (0.00,4.32) | 0.000 |

**(E) Major vascular complications (F) Acute kidney injury**

| **Loop** | **IF** | **seIF** | **z_value** | **p_value** | **CI_95** | **Loop_Heterog_tau2** |  | **Loop** | **IF** | **seIF** | **z_value** | **p_value** | **CI_95** | **Loop_Heterog_tau2** |
| --- | --- | --- | --- | --- | --- | --- | --- | --- | --- | --- | --- | --- | --- | --- |
| Lotus-DFM-Sapien3 | 1.188 | 1.098 | 1.082 | 0.279 | (0.00,3.34) | 0.000 |  | Acurate-Portico-Lotus | 1.226 | 0.772 | 1.588 | 0.112 | (0.00,2.74) | 0.000 |
| Evolut-DFM-Sapien3 | 1.039 | 1.848 | 0.562 | 0.574 | (0.00,4.66) | 0.065 |  | Evolut-Lotus-DFM | 1.065 | 0.797 | 1.337 | 0.181 | (0.00,2.63) | 0.000 |
| Evolut-Lotus-DFM | 0.793 | 2.297 | 0.345 | 0.730 | (0.00,5.30) | 0.975 |  | Lotus-DFM-Sapien | 0.807 | 0.755 | 1.068 | 0.286 | (0.00,2.29) | 0.000 |
| Evolut-Acurate-Lotus | 0.732 | 0.777 | 0.943 | 0.346 | (0.00,2.26) | 0.452 |  | Evolut-Acurate-Lotus | 0.781 | 0.636 | 1.229 | 0.219 | (0.00,2.03) | 0.110 |
| Evolut-Lotus-Sapien3 | 0.640 | 0.475 | 1.349 | 0.177 | (0.00,1.57) | 0.148 |  | Acurate-Lotus-Sapien | 0.600 | 0.454 | 1.323 | 0.186 | (0.00,1.49) | 0.000 |
| Evolut-Portico-Lotus | 0.467 | 0.741 | 0.630 | 0.529 | (0.00,1.92) | 0.290 |  | Portico-Lotus-Sapien | 0.514 | 0.784 | 0.656 | 0.512 | (0.00,2.05) | 0.291 |
| Evolut-Acurate-Portico | 0.324 | 0.561 | 0.577 | 0.564 | (0.00,1.42) | 0.290 |  | Evolut-Portico-Sapien | 0.426 | 0.369 | 1.155 | 0.248 | (0.00,1.15) | 0.031 |
| Acurate-Portico-Lotus | 0.215 | 0.829 | 0.259 | 0.795 | (0.00,1.84) | 0.314 |  | Evolut-Lotus-Sapien | 0.334 | 0.438 | 0.762 | 0.446 | (0.00,1.19) | 0.060 |
| Acurate-Portico-Sapien3 | 0.157 | 0.471 | 0.333 | 0.740 | (0.00,1.08) | 0.216 |  | Evolut-Acurate-Sapien | 0.315 | 0.266 | 1.183 | 0.237 | (0.00,0.84) | 0.000 |
| Evolut-Acurate-Sapien3 | 0.137 | 0.316 | 0.434 | 0.664 | (0.00,0.76) | 0.091 |  | Acurate-Portico-Sapien | 0.145 | 0.592 | 0.245 | 0.806 | (0.00,1.30) | 0.232 |
| Evolut-Portico-Sapien3 | 0.070 | 0.500 | 0.139 | 0.889 | (0.00,1.05) | 0.295 |  | Evolut-Acurate-Portico | 0.139 | 0.642 | 0.216 | 0.829 | (0.00,1.40) | 0.183 |
| Acurate-Lotus-Sapien3 | 0.026 | 0.464 | 0.057 | 0.955 | (0.00,0.94) | 0.113 |  | Evolut-DFM-Sapien | 0.083 | 0.832 | 0.100 | 0.921 | (0.00,1.71) | 0.020 |
| Portico-Lotus-Sapien3 | 0.013 | 0.597 | 0.022 | 0.982 | (0.00,1.18) | 0.171 |  | Evolut-Portico-Lotus | 0.020 | 0.673 | 0.030 | 0.976 | (0.00,1.34) | 0.090 |

**(G) Permanent pacemaker implantation (H) Procedural mortality**

| **Loop** | **IF** | **seIF** | **z_value** | **p_value** | **CI_95** | **Loop_Heterog_tau2** |  | **Loop** | **IF** | **seIF** | **z_value** | **p_value** | **CI_95** | **Loop_Heterog_tau2** |
| --- | --- | --- | --- | --- | --- | --- | --- | --- | --- | --- | --- | --- | --- | --- |
| Evolut-Portico-DFM | 0.583 | 0.840 | 0.694 | 0.488 | (0.00,2.23) | 0.146 |  | Acurate-Portico-Sapien3 | 2.337 | 1.42 | 1.646 | 0.100 | (0.00,5.12) | 0.000 |
| Evolut-DFM-Sapien3 | 0.515 | 0.598 | 0.861 | 0.389 | (0.00,1.69) | 0.033 |  | Evolut-Acurate-Portico | 1.985 | 1.762 | 1.127 | 0.260 | (0.00,5.44) | 0.000 |
| Evolut-Lotus-DFM | 0.506 | 0.574 | 0.882 | 0.378 | (0.00,1.63) | 0.000 |  | Evolut-Lotus-Sapien3 | 1.843 | 1.116 | 1.651 | 0.099 | (0.00,4.03) | 0.000 |
| Evolut-Acurate-Sapien3 | 0.410 | 0.165 | 2.493 | 0.013 | (0.09,0.73) | 0.031 |  | Acurate-Lotus-Sapien3 | 1.793 | 1.354 | 1.324 | 0.186 | (0.00,4.45) | 0.000 |
| Evolut-Acurate-Lotus | 0.237 | 0.270 | 0.877 | 0.380 | (0.00,0.77) | 0.000 |  | Lotus-DFM-Sapien3 | 1.476 | 2.378 | 0.621 | 0.535 | (0.00,6.14) | 0.000 |
| Acurate-Lotus-Sapien3 | 0.212 | 0.251 | 0.845 | 0.398 | (0.00,0.70) | 0.010 |  | Evolut-Portico-Lotus | 1.413 | 1.817 | 0.778 | 0.437 | (0.00,4.97) | 0.000 |
| Portico-Lotus-DFM | 0.203 | 0.676 | 0.300 | 0.764 | (0.00,1.53) | 0.000 |  | Evolut-Portico-Sapien3 | 0.996 | 1.271 | 0.784 | 0.433 | (0.00,3.49) | 0.000 |
| Acurate-Portico-Sapien3 | 0.128 | 0.311 | 0.414 | 0.679 | (0.00,0.74) | 0.141 |  | Evolut-Acurate-Lotus | 0.806 | 2.217 | 0.364 | 0.716 | (0.00,5.15) | 0.928 |
| Evolut-Lotus-Sapien3 | 0.093 | 0.213 | 0.440 | 0.660 | (0.00,0.51) | 0.030 |  | Acurate-Portico-Lotus | 0.674 | 2.167 | 0.311 | 0.756 | (0.00,4.92) | 0.000 |
| Portico-Lotus-Sapien3 | 0.082 | 0.392 | 0.208 | 0.835 | (0.00,0.85) | 0.129 |  | Evolut-Acurate-Sapien3 | 0.228 | 0.914 | 0.249 | 0.803 | (0.00,2.02) | 0.000 |
| Evolut-Portico-Sapien3 | 0.078 | 0.226 | 0.347 | 0.729 | (0.00,0.52) | 0.047 |  | Portico-Lotus-Sapien3 | 0.146 | 1.639 | 0.089 | 0.929 | (0.00,3.36) | 0.000 |
| Evolut-Acurate-Portico | 0.073 | 0.329 | 0.223 | 0.824 | (0.00,0.72) | 0.118 |  |  |  |  |  |  |  |  |
| Acurate-Portico-Lotus | 0.062 | 0.393 | 0.158 | 0.875 | (0.00,0.83) | 0.050 |  |  |  |  |  |  |  |  |
| Portico-DFM-Sapien3 | 0.025 | 0.786 | 0.031 | 0.975 | (0.00,1.57) | 0.284 |  |  |  |  |  |  |  |  |
| Lotus-DFM-Sapien3 | 0.015 | 0.396 | 0.037 | 0.971 | (0.00,0.79) | 0.024 |  |  |  |  |  |  |  |  |
| Evolut-Portico-Lotus | 0.000 | 0.374 | 0.001 | 0.999 | (0.00,0.73) | 0.076 |  |  |  |  |  |  |  |  |

**(I) No correct positioning (J) Moderate-to-severe paravalvular leak**

| **Loop** | **IF** | **seIF** | **z_value** | **p_value** | **CI_95** | **Loop_Heterog_tau2** |  | **Loop** | **IF** | **seIF** | **z_value** | **p_value** | **CI_95** | **Loop_Heterog_tau2** |
| --- | --- | --- | --- | --- | --- | --- | --- | --- | --- | --- | --- | --- | --- | --- |
| Lotus-DFM-Sapien3 | 2.862 | 2.044 | 1.401 | 0.161 | (0.00,6.87) | 0.000 |  | Portico-Lotus-Sapien3 | 1.171 | 0.834 | 1.404 | 0.160 | (0.00,2.80) | 0.000 |
| Acurate-Portico-Lotus | 1.383 | 1.795 | 0.771 | 0.441 | (0.00,4.90) | 0.000 |  | Evolut-Lotus-DFM | 0.944 | 1.358 | 0.695 | 0.487 | (0.00,3.61) | 0.058 |
| Evolut-Acurate-Lotus | 1.334 | 1.570 | 0.850 | 0.395 | (0.00,4.41) | 0.000 |  | Acurate-Portico-Lotus | 0.861 | 0.815 | 1.057 | 0.290 | (0.00,2.46) | 0.000 |
| Acurate-Portico-Sapien3 | 0.822 | 0.865 | 0.950 | 0.342 | (0.00,2.52) | 0.000 |  | Evolut-DFM-Sapien3 | 0.688 | 1.183 | 0.581 | 0.561 | (0.00,3.01) | 0.347 |
| Evolut-Acurate-Sapien3 | 0.699 | 0.593 | 1.180 | 0.238 | (0.00,1.86) | 0.049 |  | Evolut-Portico-Sapien3 | 0.559 | 0.464 | 1.203 | 0.229 | (0.00,1.47) | 0.119 |
| Acurate-Lotus-Sapien3 | 0.477 | 1.336 | 0.357 | 0.721 | (0.00,3.09) | 0.000 |  | Evolut-Portico-Lotus | 0.520 | 0.780 | 0.666 | 0.505 | (0.00,2.05) | 0.000 |
| Portico-Lotus-Sapien3 | 0.425 | 1.643 | 0.259 | 0.796 | (0.00,3.64) | 0.000 |  | Evolut-Acurate-Sapien3 | 0.501 | 0.259 | 1.937 | 0.053 | (0.00,1.01) | 0.016 |
| Evolut-Acurate-Portico | 0.340 | 0.978 | 0.348 | 0.728 | (0.00,2.26) | 0.298 |  | Acurate-Lotus-Sapien3 | 0.432 | 0.447 | 0.965 | 0.334 | (0.00,1.31) | 0.000 |
| Evolut-Portico-Sapien3 | 0.122 | 0.789 | 0.155 | 0.877 | (0.00,1.67) | 0.000 |  | Evolut-Acurate-Portico | 0.329 | 0.302 | 1.088 | 0.276 | (0.00,0.92) | 0.000 |
| Evolut-Portico-Lotus | 0.024 | 1.684 | 0.014 | 0.989 | (0.00,3.32) | 0.000 |  | Evolut-Lotus-Sapien3 | 0.144 | 0.402 | 0.359 | 0.720 | (0.00,0.93) | 0.000 |
| Evolut-Lotus-Sapien3 | 0.016 | 1.298 | 0.012 | 0.990 | (0.00,2.56) | 0.000 |  | Evolut-Acurate-Lotus | 0.083 | 0.454 | 0.184 | 0.854 | (0.00,0.97) | 0.000 |
|  |  |  |  |  |  |  |  | Lotus-DFM-Sapien3 | 0.083 | 1.400 | 0.060 | 0.953 | (0.00,2.83) | 0.454 |
|  |  |  |  |  |  |  |  | Acurate-Portico-Sapien3 | 0.007 | 0.450 | 0.017 | 0.987 | (0.00,0.89) | 0.054 |

**(K) Prosthesis patient mismatch (L) Mean aortic valve gradients**

| **Loop** | **IF** | **seIF** | **z_value** | **p_value** | **CI_95** | **Loop_Heterog_tau2** |  | **Loop** | **IF** | **seIF** | **z_value** | **p_value** | **CI_95** | **Loop_Heterog_tau2** |
| --- | --- | --- | --- | --- | --- | --- | --- | --- | --- | --- | --- | --- | --- | --- |
| Acurate-Lotus-Sapien3 | 0.976 | 0.366 | 2.670 | 0.008 | (0.26,1.69) | 0.000 |  | Evolut-Acurate-Sapien3 | 1.375 | 0.621 | 2.215 | 0.027 | (0.16,2.59) | 0.900 |
| Evolut-Portico-Sapien3 | 0.795 | 0.474 | 1.677 | 0.094 | (0.00,1.72) | 0.070 |  | Evolut-Lotus-Sapien3 | 1.146 | 0.897 | 1.278 | 0.201 | (0.00,2.90) | 0.599 |
| Portico-Lotus-Sapien3 | 0.774 | 0.481 | 1.609 | 0.108 | (0.00,1.72) | 0.000 |  | Acurate-Portico-Sapien3 | 0.985 | 0.931 | 1.058 | 0.290 | (0.00,2.81) | 0.703 |
| Evolut-Portico-Lotus | 0.710 | 0.493 | 1.440 | 0.150 | (0.00,1.68) | 0.000 |  | Acurate-Portico-Lotus | 0.888 | 0.914 | 0.971 | 0.331 | (0.00,2.68) | 0.065 |
| Acurate-Portico-Lotus | 0.620 | 0.504 | 1.231 | 0.219 | (0.00,1.61) | 0.000 |  | Evolut-Portico-Lotus | 0.869 | 1.544 | 0.563 | 0.574 | (0.00,3.89) | 1.046 |
| Evolut-Acurate-Sapien3 | 0.509 | 0.227 | 2.238 | 0.025 | (0.06,0.96) | 0.016 |  | Lotus-DFM-Sapien3 | 0.773 | 2.208 | 0.350 | 0.726 | (0.00,5.10) | 2.784 |
| Evolut-Acurate-Portico | 0.319 | 0.458 | 0.695 | 0.487 | (0.00,1.22) | 0.077 |  | Evolut-Portico-Sapien3 | 0.598 | 0.694 | 0.862 | 0.389 | (0.00,1.96) | 0.706 |
| Evolut-Lotus-Sapien3 | 0.254 | 0.453 | 0.560 | 0.575 | (0.00,1.14) | 0.066 |  | Evolut-Acurate-Lotus | 0.184 | 1.429 | 0.129 | 0.897 | (0.00,2.98) | 1.316 |
| Lotus-DFM-Sapien3 | 0.245 | 0.964 | 0.254 | 0.799 | (0.00,2.13) | 0.133 |  | Evolut-Acurate-Portico | 0.122 | 0.726 | 0.168 | 0.867 | (0.00,1.55) | 0.666 |
| Evolut-Lotus-DFM | 0.098 | 0.920 | 0.107 | 0.915 | (0.00,1.90) | 0.000 |  | Acurate-Lotus-Sapien3 | 0.122 | 1.094 | 0.111 | 0.911 | (0.00,2.27) | 1.066 |
| Evolut-Acurate-Lotus | 0.054 | 0.435 | 0.124 | 0.901 | (0.00,0.91) | 0.000 |  | Portico-Lotus-Sapien3 | 0.064 | 0.750 | 0.085 | 0.932 | (0.00,1.53) | 0.000 |
| Acurate-Portico-Sapien3 | 0.046 | 0.385 | 0.121 | 0.904 | (0.00,0.80) | 0.019 |  |  |  |  |  |  |  |  |
| Evolut-DFM-Sapien3 | 0.031 | 0.946 | 0.032 | 0.974 | (0.00,1.89) | 0.072 |  |  |  |  |  |  |  |  |

**Table 10.** **Network side-split all for each endpoint**

**(A) Device success (B) Mortality**

| **Side** | **Direct** |  | **Indirect** |  | **Difference** |  | **P>\|z\|** |  | **Side** | **Direct** |  | **Indirect** |  | **Difference** |  | **P>\|z\|** |
| --- | --- | --- | --- | --- | --- | --- | --- | --- | --- | --- | --- | --- | --- | --- | --- | --- |
|  | **Coef.** | **Std. Err.** | **Coef.** | **Std. Err.** | **Coef.** | **Std. Err.** |  |  |  | **Coef.** | **Std.Err.** | **Coef.** | **Std.Err.** | **Coef.** | **Std.Err.** |  |
| Evolut-Acurate | -0.0223048 | 0.1835036 | 0.1704628 | 0.2707072 | -0.1927677 | 0.3335576 | 0.563 |  | Evolut-Acurate | -0.0636435 | 0.1981073 | -0.0275636 | 0.2095755 | -0.0360799 | 0.2858106 | 0.900 |
| Evolut-Portico | 0.0734036 | 0.2395888 | -0.1756629 | 0.482429 | 0.2490665 | 0.5351567 | 0.642 |  | Evolut-Portico | -0.0395307 | 0.2237847 | 0.2518036 | 0.3528019 | -0.2913344 | 0.4033496 | 0.470 |
| Evolut-Lotus | -0.2288988 | 0.7077967 | -0.0202698 | 0.282528 | -0.2086289 | 0.7455206 | 0.780 |  | Evolut-Lotus | 0.3750453 | 0.3273889 | 0.1026237 | 0.2631504 | 0.2724216 | 0.3894816 | 0.484 |
| Evolut-Sapien3 | 0.1934876 | 0.1498801 | -0.2278195 | 0.2805382 | 0.4213071 | 0.3204185 | 0.189 |  | Evolut-DFM | 0.3050228 | 1.279328 | -0.5590366 | 0.5403386 | 0.8640594 | 1.2752500 | 0.498 |
| Acurate-Portico | -0.0938029 | 0.2762537 | 0.1465688 | 0.3916599 | -0.2403717 | 0.4736407 | 0.612 |  | Evolut-Sapien3 | -0.2476024 | 0.0441075 | -0.3813531 | 0.3250735 | 0.1337507 | 0.3277639 | 0.683 |
| Acurate-Lotus | -0.8345337 | 0.9674371 | -0.0156717 | 0.284465 | -0.8188619 | 1.014844 | 0.420 |  | Acurate-Portico | 0.2865964 | 0.2720318 | -0.3100592 | 0.3785113 | 0.5966556 | 0.4594415 | 0.194 |
| Acurate-Sapien3 | -0.0098449 | 0.1690064 | 0.2460774 | 0.280425 | -0.2559224 | 0.3345309 | 0.444 |  | Acurate-Lotus | 1.00502 | 0.3944746 | -0.234549 | 0.3189951 | 1.239569 | 0.4994009 | 0.013 |
| Portico-Lotus | -0.5419231 | 0.8358752 | 0.0090084 | 0.3434705 | -0.5509315 | 0.9019442 | 0.541 |  | Acurate-Sapien3 | -0.1385462 | 0.1666331 | -0.3583567 | 0.2486029 | 0.2198105 | 0.2877447 | 0.445 |
| Portico-Sapien3 | 0.1058753 | 0.3094669 | 0.0467114 | 0.3099056 | 0.0591639 | 0.4263795 | 0.890 |  | Portico-Lotus | -0.0142773 | 0.4659885 | 0.2673852 | 0.3521711 | -0.2816625 | 0.5822266 | 0.629 |
| Lotus-DFM | 1.089802 | 0.9815579 | -1.501597 | 0.6033292 | 2.591398 | 1.152155 | 0.025 |  | Portico-Sapien3 | -0.3334808 | 0.2505286 | -0.2319417 | 0.2828568 | -0.1015391 | 0.3607606 | 0.778 |
| Lotus-Sapien3 | 0.0218759 | 0.2412405 | 1.550138 | 0.7361161 | -1.528262 | 0.7571413 | 0.044 |  | Lotus-DFM | -0.588693 | 0.7548921 | -0.7701438 | 0.6884096 | 0.1814508 | 0.9525755 | 0.849 |
| DFM-Sapien3 | 1.533023 | 0.5556214 | -1.058375 | 1.00956 | 2.591398 | 1.152156 | 0.025 |  | Lotus-Sapien3 | -0.3221076 | 0.2288821 | -1.093202 | 0.4181567 | 0.7710945 | 0.4320261 | 0.074 |
|  |  |  |  |  |  |  |  |  | DFM-Sapien3 | 0.4957565 | 0.6005797 | -0.30728 | 0.7949953 | 0.8030365 | 0.8818252 | 0.362 |

**(C) Stroke (D) Major/life threatening bleeding**

| **Side** | **Direct** |  | **Indirect** |  | **Difference** |  | **P>\|z\|** |  | **Side** | **Direct** |  | **Indirect** |  | **Difference** |  | **P>\|z\|** |
| --- | --- | --- | --- | --- | --- | --- | --- | --- | --- | --- | --- | --- | --- | --- | --- | --- |
|  | **Coef.** | **Std. Err.** | **Coef.** | **Std. Err.** | **Coef.** | **Std. Err.** |  |  |  | **Coef.** | **Std. Err.** | **Coef.** | **Std. Err.** | **Coef.** | **Std. Err.** |  |
| Evolut-Acurate | -0.2113577 | 0.2192811 | -0.7140836 | 0.3012824 | 0.5027259 | 0.3672323 | 0.171 |  | Evolut-Acurate | -0.090194 | 0.1815414 | 0.4835815 | 0.2410014 | -0.5737756 | 0.2992389 | 0.055 |
| Evolut-Portico | 0.11151090 | 0.3098334 | -0.8911373 | 0.4744723 | 1.0026480 | 0.5449731 | 0.066 |  | Evolut-Portico | -0.0634574 | 0.213736 | 0.5737019 | 0.4138383 | -0.6371592 | 0.4557369 | 0.162 |
| Evolut-Lotus | 0.42113850 | 0.3953556 | 0.1602388 | 0.3063909 | 0.2608997 | 0.4849998 | 0.591 |  | Evolut-Lotus | 0.9650715 | 0.3429646 | -0.1657766 | 0.2672725 | 1.130848 | 0.4301751 | 0.009 |
| Evolut-DFM | 1.0966080 | 2.0098990 | -1.407039 | 0.7017956 | 2.5036470 | 2.1281730 | 0.239 |  | Evolut-DFM | 1.094334 | 2.029249 | -0.2340544 | 0.6472376 | 1.328388 | 2.129391 | 0.533 |
| Evolut-Sapien3 | -0.5786242 | 0.1491184 | 0.0602348 | 0.3365019 | -0.6388590 | 0.3673300 | 0.082 |  | Evolut-Sapien3 | 0.2256536 | 0.1400949 | -0.0212878 | 0.2869805 | 0.2469414 | 0.3168959 | 0.436 |
| Acurate-Portico | 0.11201290 | 0.4127650 | 0.3072475 | 0.4145384 | -0.1952347 | 0.5836455 | 0.738 |  | Acurate-Portico | 0.0252029 | 0.2776381 | -0.1435827 | 0.3274153 | 0.1687856 | 0.4246939 | 0.691 |
| Acurate-Lotus | 1.13867800 | 0.5414253 | 0.4572177 | 0.3168736 | 0.6814604 | 0.6323024 | 0.281 |  | Acurate-Lotus | 0.4194017 | 0.342626 | -0.0181687 | 0.2832252 | 0.4375704 | 0.4310206 | 0.310 |
| Acurate-Sapien3 | 0.01209460 | 0.2112244 | -0.3029987 | 0.2979355 | 0.3150933 | 0.3589240 | 0.380 |  | Acurate-Sapien3 | -0.0195567 | 0.1449554 | 0.4216935 | 0.2840333 | -0.4412502 | 0.3147784 | 0.161 |
| Portico-Lotus | 1.14876300 | 0.7254033 | 0.2292445 | 0.3774683 | 0.9195187 | 0.8141719 | 0.259 |  | Portico-Lotus | 0.3575185 | 0.4271264 | 0.1125832 | 0.3332984 | 0.2449353 | 0.5414048 | 0.651 |
| Portico-Sapien3 | -0.1033184 | 0.3224285 | -0.6696068 | 0.4276709 | 0.5662884 | 0.5167876 | 0.273 |  | Portico-Sapien3 | -0.0091555 | 0.2324036 | 0.3674863 | 0.3192449 | -0.3766418 | 0.3811708 | 0.323 |
| Lotus-DFM | -1.2911280 | 0.7514985 | -1.567277 | 0.9564832 | 0.2761486 | 1.0883590 | 0.800 |  | Lotus-DFM | 0.1637817 | 0.7795482 | -1.081044 | 0.8800207 | 1.244826 | 1.127672 | 0.270 |
| Lotus-Sapien3 | -0.6635316 | 0.2275037 | -1.218581 | 0.5771052 | 0.5550498 | 0.5995964 | 0.355 |  | Lotus-Sapien3 | -0.0003664 | 0.1974586 | -0.6930651 | 0.444076 | 0.6926987 | 0.4562661 | 0.129 |
| DFM-Sapien3 | 1.0660100 | 0.8510511 | 0.2621008 | 0.8517235 | 0.8039088 | 1.0940220 | 0.462 |  | DFM-Sapien3 | 0.980933 | 0.7871271 | -0.519904 | 0.8465975 | 1.500837 | 1.092965 | 0.170 |

**(E) Major vascular complications (F) Acute kidney injury**

| **Side** | **Direct** |  | **Indirect** |  | **Difference** |  | **P>\|z\|** |  | **Side** | **Direct** |  | **Indirect** |  | **Difference** |  | **P>\|z\|** |
| --- | --- | --- | --- | --- | --- | --- | --- | --- | --- | --- | --- | --- | --- | --- | --- | --- |
|  | **Coef.** | **Std. Err.** | **Coef.** | **Std. Err.** | **Coef.** | **Std. Err.** |  |  |  | **Coef.** | **Std. Err.** | **Coef.** | **Std. Err.** | **Coef.** | **Std. Err.** |  |
| Evolut-Acurate | 0.0860128 | 0.1885816 | 0.2481223 | 0.2986564 | -0.1621095 | 0.3499443 | 0.643 |  | Evolut-Acurate | -0.1234009 | 0.1740847 | 0.3962063 | 0.266464 | -0.5196072 | 0.3115997 | 0.095 |
| Evolut-Portico | -0.0721794 | 0.2200999 | 0.1022634 | 0.4990666 | -0.1744428 | 0.5365954 | 0.745 |  | Evolut-Portico | -0.2782443 | 0.2139551 | 0.5893785 | 0.4578584 | -0.8676228 | 0.4670526 | 0.063 |
| Evolut-Lotus | 0.3922705 | 0.3544545 | -0.4021434 | 0.2988198 | 0.7944139 | 0.4547997 | 0.081 |  | Evolut-Lotus | -0.1043031 | 0.2716026 | 0.2443725 | 0.3534783 | -0.3486755 | 0.3967927 | 0.380 |
| Evolut-DFM | 0.2853295 | 1.551195 | -0.5662524 | 0.6511823 | 0.8515819 | 1.602337 | 0.595 |  | Evolut-DFM | 0.9232627 | 0.5975703 | 0.3093231 | 0.5739751 | 0.6139395 | 0.8010926 | 0.443 |
| Evolut-Sapien3 | 0.0191898 | 0.1670599 | 0.1170641 | 0.2956867 | -0.0978743 | 0.3380154 | 0.772 |  | Evolut-Sapien3 | 0.0148753 | 0.1302906 | -0.2863023 | 0.3968084 | 0.3011776 | 0.4181047 | 0.471 |
| Acurate-Portico | -0.2966084 | 0.2529169 | 0.1003029 | 0.3812391 | -0.3969114 | 0.4446005 | 0.372 |  | Acurate-Portico | -0.194842 | 0.2627304 | 0.0125939 | 0.4825657 | -0.2074359 | 0.5213124 | 0.691 |
| Acurate-Lotus | -0.33392 | 0.3629242 | -0.1167528 | 0.2942349 | -0.2171672 | 0.4511896 | 0.630 |  | Acurate-Lotus | -0.3706406 | 0.349835 | 0.386165 | 0.3559798 | -0.7568056 | 0.4802213 | 0.115 |
| Acurate-Sapien3 | -0.1357707 | 0.1640302 | 0.0768737 | 0.3044789 | -0.2126444 | 0.3410854 | 0.533 |  | Acurate-Sapien3 | -0.0807107 | 0.1550376 | 0.2267854 | 0.3545595 | -0.3074961 | 0.3847659 | 0.424 |
| Portico-Lotus | 0.1456388 | 0.4268718 | -0.1415946 | 0.3547173 | 0.2872335 | 0.5564399 | 0.606 |  | Portico-Lotus | 0.0391692 | 0.3810325 | 0.3429853 | 0.4756896 | -0.3038161 | 0.6100876 | 0.618 |
| Portico-Sapien3 | 0.0843583 | 0.2702985 | 0.093592 | 0.3128107 | -0.0092336 | 0.4037195 | 0.982 |  | Portico-Sapien3 | 0.0526944 | 0.2553918 | 0.309497 | 0.3780303 | -0.2568026 | 0.4250792 | 0.546 |
| Lotus-DFM | 0.1792019 | 0.7970164 | -1.10363 | 0.8734118 | 1.282831 | 1.128358 | 0.256 |  | Lotus-DFM | 0.2969338 | 0.4495227 | 1.573039 | 0.7691944 | -1.276105 | 0.8408128 | 0.129 |
| Lotus-Sapien3 | 0.1563807 | 0.2143257 | -0.1852724 | 0.4820026 | 0.341653 | 0.5002374 | 0.495 |  | Lotus-Sapien3 | -0.1019174 | 0.2478789 | 0.2932772 | 0.4336103 | -0.3951946 | 0.4497195 | 0.380 |
| DFM-Sapien3 | 1.192678 | 0.7724668 | -0.3303463 | 0.8450636 | 1.523024 | 1.053954 | 0.148 |  | DFM-Sapien3 | -0.9651537 | 0.5636509 | -0.1574274 | 0.6485436 | -0.8077262 | 0.8656297 | 0.351 |

**(G) Permanent pacemaker implantation (H) Procedural mortality**

| **Side** | **Direct** |  | **Indirect** |  | **Difference** |  | **P>\|z\|** |  | **Side** | **Direct** |  | **Indirect** |  | **Difference** |  | **P>\|z\|** |
| --- | --- | --- | --- | --- | --- | --- | --- | --- | --- | --- | --- | --- | --- | --- | --- | --- |
|  | **Coef.** | **Std. Err.** | **Coef.** | **Std. Err.** | **Coef.** | **Std. Err.** |  |  |  | **Coef.** | **Std. Err.** | **Coef.** | **Std. Err.** | **Coef.** | **Std. Err.** |  |
| Evolut-Acurate | -0.711618 | 0.1338689 | -0.9332772 | 0.1751532 | 0.2216592 | 0.2123784 | 0.297 |  | Evolut-Acurate | .1232737 | 0.613967 | 0.5326099 | 0.6696605 | -0.4093361 | 0.900531 | 0.649 |
| Evolut-Portico | -0.1109539 | 0.1337078 | -0.2735001 | 0.244657 | 0.1625462 | 0.2704316 | 0.548 |  | Evolut-Portico | -1.112895 | 0.779276 | -1.532348 | 1.303583 | 0.4194527 | 1.237344 | 0.735 |
| Evolut-Lotus | 0.5647095 | 0.192756 | 0.6509866 | 0.1794349 | -0.0862771 | 0.2573995 | 0.737 |  | Evolut-Lotus | 1.875374 | 0.9102073 | -0.3556254 | 0.7386449 | 2.230999 | 1.201003 | 0.063 |
| Evolut-DFM | -0.381089 | 0.4543773 | -0.8189723 | 0.2501324 | 0.4378834 | 0.4893732 | 0.371 |  | Evolut-Sapien3 | -0.1236976 | 0.3317914 | -0.6938587 | 0.8870536 | 0.570161 | 0.935329 | 0.542 |
| Evolut-Sapien3 | -0.4819535 | 0.0725266 | -0.2596592 | 0.1917663 | -0.2222943 | 0.2052494 | 0.279 |  | Acurate-Portico | -.0580008 | 1.001961 | -4.452954 | 1.446587 | 4.394953 | 1.759746 | 0.013 |
| Acurate-Portico | 0.7311239 | 0.1819671 | 0.4935511 | 0.2468328 | 0.2375729 | 0.3119152 | 0.446 |  | Acurate-Lotus | 1.402115 | 1.081426 | -0.4594488 | 0.8071944 | 1.861563 | 1.416888 | 0.189 |
| Acurate-Lotus | 1.48517 | 0.2897826 | 1.366559 | 0.1925906 | 0.1186113 | 0.3499928 | 0.735 |  | Acurate-Sapien3 | -0.4795811 | 0.417564 | -0.6073476 | 0.8646957 | 0.1277665 | 0.898926 | 0.887 |
| Acurate-Sapien3 | 0.4234003 | 0.1351038 | 0.1965273 | 0.1699002 | 0.226873 | 0.2115356 | 0.283 |  | Portico-Lotus | 2.13873 | 1.101861 | 1.154494 | 1.250649 | 0.9842367 | 1.59723 | 0.538 |
| Portico-Lotus | 0.7775114 | 0.3102704 | 0.7479876 | 0.2020756 | 0.0295238 | 0.3706086 | 0.937 |  | Portico-Sapien3 | 1.405885 | 0.8190856 | 0.0872258 | 1.011309 | 1.318659 | 0.986796 | 0.181 |
| Portico-DFM | -0.7732853 | 0.8341774 | -0.568514 | 0.2639175 | -0.2047713 | 0.8719819 | 0.814 |  | Lotus-DFM | 0.4606808 | 1.64071 | -1.303661 | 1.716465 | 1.764342 | 2.374485 | 0.457 |
| Portico-Sapien3 | -0.2554532 | 0.1884169 | -0.3495432 | 0.1630207 | 0.09409 | 0.2443839 | 0.700 |  | Lotus-Sapien3 | -0.461576 | 0.5185971 | -2.544502 | 1.198848 | 2.082926 | 1.254045 | 0.097 |
| Lotus-DFM | -1.403009 | 0.302594 | -1.266705 | 0.3364345 | -0.1363042 | 0.4286636 | 0.751 |  | DFM-Sapien3 | 0.4946962 | 1.640675 | -1.269645 | 1.716498 | 1.764342 | 2.374485 | 0.457 |
| Lotus-Sapien3 | -1.039578 | 0.1395068 | -1.183593 | 0.2757685 | 0.1440141 | 0.2995739 | 0.631 |  |  |  |  |  |  |  |  |  |
| DFM-Sapien3 | 0.261251 | 0.2495018 | 0.3203279 | 0.3774405 | -0.059077 | 0.4133901 | 0.886 |  |  |  |  |  |  |  |  |  |

**(I) No correct positioning (J) Moderate-to-severe paravalvular leak**

| **Side** | **Direct** |  | **Indirect** |  | **Difference** |  | **P>\|z\|** |  | **Side** | **Direct** |  | **Indirect** |  | **Difference** |  | **P>\|z\|** |
| --- | --- | --- | --- | --- | --- | --- | --- | --- | --- | --- | --- | --- | --- | --- | --- | --- |
|  | **Coef.** | **Std. Err.** | **Coef.** | **Std. Err.** | **Coef.** | **Std. Err.** |  |  |  | **Coef.** | **Std. Err.** | **Coef.** | **Std. Err.** | **Coef.** | **Std. Err.** |  |
| Evolut-Acurate | -0.6480453 | 0.3540762 | -0.1875213 | 0.5924625 | -0.4605239 | 0.6929121 | 0.506 |  | Evolut-Acurate | 0.2048628 | 0.1635703 | 0.4863784 | 0.2784999 | -0.2815155 | 0.3211615 | 0.381 |
| Evolut-Portico | 0.0692889 | 0.3536852 | -0.4120342 | 0.9165804 | 0.4813231 | 0.9340162 | 0.606 |  | Evolut-Portico | 0.4240519 | 0.1894417 | 1.231042 | 0.4667026 | -0.8069905 | 0.4995878 | 0.106 |
| Acurate-Lotus | -0.2702092 | 0.8267539 | -1.606601 | 0.7306155 | 1.336392 | 0.8392639 | 0.111 |  | Evolut-Lotus | -1.401999 | 0.3155557 | -1.179691 | 0.4018962 | -0.222308 | 0.454196 | 0.625 |
| Acurate-Sapien3 | -1.082247 | 0.3242158 | -1.912971 | 0.5457515 | 0.8307242 | 0.6321412 | 0.189 |  | Evolut-DFM | 0.5734266 | 0.7755543 | -0.5544252 | 0.6212102 | 1.127852 | 0.9847882 | 0.252 |
| Acurate-Portico | 0.4976534 | 0.4985117 | 0.682004 | 0.7808886 | -0.1843506 | 0.9461633 | 0.846 |  | Evolut-Sapien3 | -0.5419768 | 0.1614223 | -0.9223822 | 0.275336 | 0.3804054 | 0.3213292 | 0.236 |
| Acurate-Lotus | -1.954986 | 0.911115 | 0.1013681 | 0.7379294 | -2.056354 | 0.959133 | 0.032 |  | Acurate-Portico | 0.3784461 | 0.2359459 | 0.0102932 | 0.3296087 | 0.3681529 | 0.3978363 | 0.355 |
| Acurate-Sapien3 | -0.9645755 | 0.3244006 | -0.1171449 | 0.6104271 | -0.8474306 | 0.6908107 | 0.220 |  | Acurate-Lotus | -1.682479 | 0.3490837 | -1.500307 | 0.3763214 | -0.1821721 | 0.4594408 | 0.692 |
| Portico-Lotus | -1.490225 | 1.237908 | -0.9771607 | 0.8550653 | -0.5130638 | 1.445685 | 0.723 |  | Acurate-Sapien3 | -1.039243 | 0.1643425 | -0.5545664 | 0.2802739 | -0.4846765 | 0.3240406 | 0.135 |
| Portico-Sapien3 | -0.9767466 | 0.5990698 | -1.592419 | 0.5258762 | 0.6156723 | 0.7763691 | 0.428 |  | Portico-Sapien3 | -1.153465 | 0.6647986 | -2.037596 | 0.3445735 | 0.8841305 | 0.73671 | 0.230 |
| Lotus-DFM | -1.761907 | 1.675714 | 1.112033 | 1.245551 | -2.87394 | 2.087921 | 0.169 |  | Lotus-DFM | -1.421801 | 0.3459657 | -1.039765 | 0.2548087 | -0.3820361 | 0.4287623 | 0.373 |
| Lotus-Sapien3 | 0.0066134 | 0.671638 | -0.9471297 | 1.039154 | 0.953743 | 1.036903 | 0.358 |  | Lotus-Sapien3 | 1.556335 | 0.9694492 | 1.006457 | 0.7101668 | 0.5498788 | 1.284125 | 0.668 |
| DFM-Sapien3 | -1.006184 | 1.049192 | 1.867753 | 1.805091 | -2.873937 | 2.087921 | 0.169 |  | DFM-Sapien3 | 0.6267772 | 0.3039351 | 0.8289547 | 0.459709 | -0.2021775 | 0.506424 | 0.690 |

**(K) Prosthesis patient mismatch (L) Mean aortic valve gradients**

| **Side** | **Direct** |  | **Indirect** |  | **Difference** |  | **P>\|z\|** |  | **Side** | **Direct** |  | **Indirect** |  | **Difference** |  | **P>\|z\|** |
| --- | --- | --- | --- | --- | --- | --- | --- | --- | --- | --- | --- | --- | --- | --- | --- | --- |
|  | **Coef.** | **Std. Err.** | **Coef.** | **Std. Err.** | **Coef.** | **Std. Err.** |  |  |  | **Coef.** | **Std. Err.** | **Coef.** | **Std. Err.** | **Coef.** | **Std. Err.** |  |
| Evolut-Acurate | 0.3286049 | 0.1164508 | -0.3652765 | 0.2134043 | 0.6938814 | 0.2434064 | 0.004 |  | Evolut-Acurate | 1.416776 | 0.4084476 | -0.0053193 | 0.5140163 | 1.422095 | 0.6533376 | 0.030 |
| Evolut-Portico | 0.5035079 | 0.180674 | -0.3320197 | 0.4041889 | 0.8355275 | 0.4313735 | 0.053 |  | Evolut-Portico | 0.8170449 | 0.3897672 | -1.203992 | 0.9724922 | 2.021037 | 1.045198 | 0.053 |
| Evolut-Lotus | 0.4032689 | 0.3583802 | 0.9171774 | 0.2869082 | -0.5139085 | 0.4489519 | 0.252 |  | Evolut-Lotus | 5.466299 | 0.8929407 | 4.478603 | 0.6036291 | 0.9876963 | 1.056788 | 0.350 |
| Evolut-DFM | 0.5753641 | 0.6821304 | 0.9756406 | 0.9227692 | -0.4002764 | 1.200752 | 0.739 |  | Evolut-DFM | 3.499399 | 0.2738433 | 4.536658 | 0.6314126 | -1.037259 | 0.6883177 | 0.132 |
| Evolut-Sapien3 | 0.8436472 | 0.1368031 | 1.240526 | 0.2587871 | -0.3968788 | 0.2946237 | 0.178 |  | Evolut-Sapien3 | -0.7638509 | 0.5252582 | 0.4948557 | 0.7305989 | -1.258707 | 0.9019194 | 0.163 |
| Acurate-Portico | 0.093204 | 0.1925153 | 0.9866503 | 0.4446123 | -0.8934463 | 0.4699788 | 0.057 |  | Acurate-Portico | 4.084788 | 0.8913004 | 3.813873 | 0.6699571 | 0.270915 | 1.095512 | 0.805 |
| Acurate-Lotus | 0.0686282 | 0.359329 | 0.8197657 | 0.2700423 | -0.7511375 | 0.4290825 | 0.080 |  | Acurate-Lotus | 3.237041 | 0.3809236 | 1.872487 | 0.5481847 | 1.364555 | 0.6663766 | 0.051 |
| Acurate-Sapien3 | 0.9589079 | 0.1637449 | 0.4337526 | 0.2152657 | 0.5251552 | 0.2700518 | 0.052 |  | Acurate-Sapien3 | 3.82168 | 1.257123 | 4.370637 | 0.6745302 | -0.5489573 | 1.418089 | 0.699 |
| Portico-Lotus | 0.4273232 | 0.4500635 | 0.2967196 | 0.3548254 | 0.1306036 | 0.5727653 | 0.820 |  | Portico-Lotus | 3.05568 | 0.6821972 | 3.177023 | 0.5141585 | -0.1213429 | 0.8528181 | 0.887 |
| Portico-Sapien3 | 1.029128 | 0.342327 | 0.3357759 | 0.236263 | 0.693352 | 0.4037847 | 0.086 |  | Portico-Sapien3 | 3.20000 | 1.264456 | 3.524388 | 0.9208262 | -0.3243886 | 1.564215 | 0.836 |
| Lotus-DFM | 0.169899 | 0.6402534 | -0.3772161 | 0.9646285 | 0.5471152 | 1.163753 | 0.638 |  | Lotus-DFM | -0.9499118 | 0.5269964 | -1.718716 | 0.9636715 | 0.7688044 | 1.072377 | 0.473 |
| Lotus-Sapien3 | 0.201677 | 0.2159818 | 0.2984239 | 0.5469336 | -0.096747 | 0.5587311 | 0.863 |  | Lotus-Sapien3 | -4.608702 | 0.7734837 | -4.284109 | 1.359951 | -0.3245928 | 1.564215 | 0.836 |
| DFM-Sapien3 | 0.238411 | 0.5667414 | 0.1099306 | 0.9310613 | 0.1284804 | 1.011222 | 0.899 |  | DFM-Sapien3 | 1.416776 | 0.4084476 | -0.0053193 | 0.5140163 | 1.422095 | 0.6533376 | 0.030 |

**Table 11. Summary of GRADE domain and procedure for pairwise effect estimate and network estimate**

| **GRADE domain** | **GRADE procedure** | |
| --- | --- | --- |
|  | **For pairwise effect estimate** | **For overall network estimate** |
| Study limitations | We assessed study limitations according to the assessment of risk of bias for each direct comparison and the contribution of each direct comparison to the network estimates (contribution matrix). We downgraded by one level when the contributions from moderate risk of bias comparisons were 50% or greater. | |
| Imprecision | We assessed imprecision according to the OR point estimate and 95% confidence interval. We downgraded the estimate if the OR point estimate is 1 or more and the lower limit of its CrI is below 0.80; or if the OR point estimate is less than 1 and the upper limit of its CrI is above 1.20. | We assessed imprecision according to the SUCRA values. We downgraded by one level when the SUCRAs were similar. |
| Inconsistency | We assessed heterogeneity according to the 95% confidence interval (CI) and 95% prediction interval (PrI). We downgraded the comparisons if there were significant differences between 95% CI and 95% PrI.  We assessed inconsistency according to the results of side splitting and we downgraded the comparisons with important inconsistency (p<0.05). | We assessed heterogeneity according to the network variance estimates (Tau^2^). We downgraded by one level when the Tau^2^ was greater than 0.36.  We assessed inconsistency according to the results of design-by treatment global test. We downgraded by one level when global inconsistency P-value < 0.05. |
| Indirectness | We assessed indirectness according to the distribution of known effect modifiers across comparisons. We have assured that transitivity assumption in our network was reasonable, so we did not downgrade for either pairwise effect estimate or overall network estimate. | |
| Publication bias | In addition to the comprehensiveness search for studies on several databases, we have managed to retrieve e and unpublished studies included in the available systematic reviews and meta-analyses. We were confident that we have all available studies so we did not downgrade GRADE for pairwise effect estimate. | We assessed publication bias according to the comparison-adjusted funnel plot. We downgraded by one level when the funnel plot was asymmetrical. Indicate a form of small-study effects |

**Table 12. Summary of our confidence in effect estimates and ranking of treatments for each endpoint**

**(A) Device success**

| **Comparison** | **Study limitations** | **Imprecision** | **Inconsistency** | | **Indirectness** | **Publication bias** | **Total** | **GRADE** |
| --- | --- | --- | --- | --- | --- | --- | --- | --- |
|  |  |  | **heterogeneity** | **Incoherence** |  |  |  |  |
| Evolut vs Acurate | -1 | -1 | -0 | -0 | -0 | -0 | -2 | Low |
| Evolut vs Portico | -1 | -1 | -0 | -0 | -0 | -0 | -2 | Low |
| Evolut vs Lotus | -0 | -1 | -0 | -0 | -0 | -0 | -1 | Moderate |
| Evolut vs Sapien3 | -0 | -0 | -0 | -0 | -0 | -0 | -0 | High |
| Acurate vs Portico | -1 | -1 | -0 | -0 | -0 | -0 | -2 | Low |
| Acurate vs Lotus | -0 | -1 | -0 | -0 | -0 | -0 | -1 | Moderate |
| Acurate vs Sapien3 | -1 | -1 | -0 | -0 | -0 | -0 | -2 | Low |
| Portico vs Lotus | -0 | -1 | -0 | -0 | -0 | -0 | -1 | Moderate |
| Portico vs Sapien3 | -1 | -1 | -0 | -0 | -0 | -0 | -2 | Low |
| Lotus vs DFM | -0 | -1 | -0 | -1 | -0 | -0 | -2 | Low |
| Lotus vs Sapien3 | -0 | -1 | -0 | -1 | -0 | -0 | -2 | Low |
| DFM vs Sapien3 | -1 | -0 | -0 | -1 | -0 | -0 | -2 | Low |
| Evolut vs DFM | -1 | -0 | -0 | -1 | -0 | -0 | -2 | Low |
| Acurate vs DFM | -1 | -0 | -0 | -1 | -0 | -0 | -2 | Low |
| Portico vs DFM | -1 | -0 | -0 | -1 | -0 | -0 | -2 | Low |
| *Network* | *-1* | *-1* | *-0* | *-0* | *-0* | *-0* | *-2* | *low* |

**(B) Mortality**

| **Comparison** | **Study limitations** | **Imprecision** | **Inconsistency** | | **Indirectness** | **Publication bias** | **Total** | **GRADE** |
| --- | --- | --- | --- | --- | --- | --- | --- | --- |
|  |  |  | **heterogeneity** | **incoherence** |  |  |  |  |
| Evolut vs Acurate | -1 | -1 | -0 | -0 | -0 | -0 | -2 | Low |
| Evolut vs Portico | -1 | -1 | -0 | -0 | -0 | -0 | -2 | Low |
| Evolut vs Lotus | -1 | -1 | -0 | -0 | -0 | -0 | -2 | Low |
| Evolut vs DFM | -1 | -1 | -0 | -0 | -0 | -0 | -2 | Low |
| Evolut vs Sapien3 | -1 | -0 | -0 | -0 | -0 | -0 | -1 | Moderate |
| Acurate vs Portico | -1 | -1 | -0 | -0 | -0 | -0 | -2 | Low |
| Acurate vs Lotus | -1 | -1 | -0 | -1 | -0 | -0 | -3 | Very low |
| Acurate vs Sapien3 | -1 | -0 | -0 | -0 | -0 | -0 | -1 | Moderate |
| Portico vs Lotus | -1 | -1 | -0 | -0 | -0 | -0 | -2 | Low |
| Portico vs Sapien3 | -1 | -0 | -0 | -0 | -0 | -0 | -1 | Moderate |
| Lotus vs DFM | -1 | -1 | -0 | -0 | -0 | -0 | -2 | Low |
| Lotus vs Sapien3 | -1 | -0 | -0 | -0 | -0 | -0 | -1 | Moderate |
| DFM vs Sapien3 | -1 | -1 | -0 | -0 | -0 | -0 | -2 | Low |
| Acurate vs DFM | -1 | -1 | -0 | -1 | -0 | -0 | -3 | Very low |
| Portico vs DFM | -1 | -1 | -0 | -1 | -0 | -0 | -3 | Very low |
| *Network* | *-1* | *-0* | *-0* | *-0* | *-0* | *-0* | *-1* | *Moderate* |

**(C) Stroke**

| **Comparison** | **Study limitations** | **Imprecision** | **Inconsistency** | | **Indirectness** | **Publication bias** | **Total** | **GRADE** |
| --- | --- | --- | --- | --- | --- | --- | --- | --- |
|  |  |  | **heterogeneity** | **incoherence** |  |  |  |  |
| Evolut vs Acurate | -1 | -0 | -1 | -0 | -0 | -0 | -2 | Low |
| Evolut vs Portico | -1 | -1 | -0 | -0 | -0 | -0 | -2 | Low |
| Evolut vs Lotus | -0 | -1 | -0 | -0 | -0 | -0 | -1 | Moderate |
| Evolut vs DFM | -1 | -0 | -0 | -0 | -0 | -0 | -1 | Moderate |
| Evolut vs Sapien3 | -0 | -0 | -0 | -0 | -0 | -0 | -0 | High |
| Acurate vs Portico | -1 | -1 | -0 | -0 | -0 | -0 | -2 | Low |
| Acurate vs Lotus | -0 | -0 | -0 | -0 | -0 | -0 | -0 | High |
| Acurate vs Sapien3 | -0 | -1 | -0 | -0 | -0 | -0 | -1 | Moderate |
| Portico vs Lotus | -1 | -1 | -0 | -0 | -0 | -0 | -2 | Low |
| Portico vs Sapien3 | -1 | -0 | -0 | -0 | -0 | -0 | -1 | Moderate |
| Lotus vs DFM | -1 | -0 | -0 | -0 | -0 | -0 | -1 | Moderate |
| Lotus vs Sapien3 | -0 | -0 | -0 | -0 | -0 | -0 | -0 | High |
| DFM vs Sapien3 | -1 | -1 | -0 | -0 | -0 | -0 | -2 | Low |
| Acurate vs DFM | -1 | -1 | -0 | -1 | -0 | -0 | -3 | Very low |
| Portico vs DFM | -1 | -1 | -0 | -1 | -0 | -0 | -3 | Very low |
| *Network* | *-1* | *-0* | *-0* | *-0* | *-0* | *-0* | *-1* | *Moderate* |

**(D) Major/life threatening bleeding**

| **Comparison** | **Study limitations** | **Imprecision** | **Inconsistency** | | **Indirectness** | **Publication bias** | **Total** | **GRADE** |
| --- | --- | --- | --- | --- | --- | --- | --- | --- |
|  |  |  | **heterogeneity** | **incoherence** |  |  |  |  |
| Evolut vs Acurate | -1 | -1 | -0 | -0 | -0 | -0 | -2 | Low |
| Evolut vs Portico | -1 | -1 | -0 | -0 | -0 | -0 | -2 | Low |
| Evolut vs Lotus | -1 | -1 | -0 | -1 | -0 | -0 | -3 | Very low |
| Evolut vs DFM | -1 | -1 | -0 | -0 | -0 | -0 | -2 | Low |
| Evolut vs Sapien3 | -1 | -0 | -0 | -0 | -0 | -0 | -1 | Moderate |
| Acurate vs Portico | -1 | -1 | -0 | -0 | -0 | -0 | -2 | Low |
| Acurate vs Lotus | -0 | -1 | -0 | -0 | -0 | -0 | -1 | Moderate |
| Acurate vs Sapien3 | -0 | -1 | -0 | -0 | -0 | -0 | -1 | Moderate |
| Portico vs Lotus | -1 | -1 | -0 | -0 | -0 | -0 | -2 | Low |
| Portico vs Sapien3 | -1 | -1 | -0 | -0 | -0 | -0 | -2 | Low |
| Lotus vs DFM | -1 | -1 | -0 | -0 | -0 | -0 | -2 | Low |
| Lotus vs Sapien3 | -0 | -1 | -0 | -0 | -0 | -0 | -1 | Moderate |
| DFM vs Sapien3 | -1 | -1 | -0 | -0 | -0 | -0 | -2 | Low |
| Acurate vs DFM | -1 | -1 | -0 | -1 | -0 | -0 | -3 | Very low |
| Portico vs DFM | -1 | -1 | -0 | -1 | -0 | -0 | -3 | Very low |
| *Network* | *-1* | *-0* | *-0* | *-1* | *-0* | *-0* | *-2* | *Low* |

**(E) Major vascular complications**

| **Comparison** | **Study limitations** | **Imprecision** | **Inconsistency** | | **Indirectness** | **Publication bias** | **Total** | **GRADE** |
| --- | --- | --- | --- | --- | --- | --- | --- | --- |
|  |  |  | **heterogeneity** | **incoherence** |  |  |  |  |
| Evolut vs Acurate | -1 | -0 | -0 | -0 | -0 | -0 | -1 | Moderate |
| Evolut vs Portico | -1 | -1 | -0 | -0 | -0 | -0 | -2 | Low |
| Evolut vs Lotus | -1 | -1 | -0 | -0 | -0 | -0 | -2 | Low |
| Evolut vs DFM | -1 | -1 | -0 | -0 | -0 | -0 | -2 | Low |
| Evolut vs Sapien3 | -1 | -1 | -0 | -0 | -0 | -0 | -2 | Low |
| Acurate vs Portico | -1 | -1 | -0 | -0 | -0 | -0 | -2 | Low |
| Acurate vs Lotus | -0 | -1 | -0 | -0 | -0 | -0 | -1 | Moderate |
| Acurate vs Sapien3 | -0 | -0 | -0 | -0 | -0 | -0 | -0 | High |
| Portico vs Lotus | -1 | -1 | -0 | -0 | -0 | -0 | -2 | Low |
| Portico vs Sapien3 | -1 | -1 | -0 | -0 | -0 | -0 | -2 | Low |
| Lotus vs DFM | -1 | -1 | -0 | -0 | -0 | -0 | -2 | Low |
| Lotus vs Sapien3 | -0 | -1 | -0 | -0 | -0 | -0 | -1 | Moderate |
| DFM vs Sapien3 | -1 | -1 | -0 | -0 | -0 | -0 | -2 | Low |
| Acurate vs DFM | -1 | -1 | -0 | -1 | -0 | -0 | -3 | Very low |
| Portico vs DFM | -1 | -1 | -0 | -1 | -0 | -0 | -3 | Very low |
| *Network* | *-1* | *-0* | *-1* | *-0* | *-0* | *-0* | *-2* | *Low* |

**(F) Acute kidney injury**

| **Comparison** | **Study limitations** | **Imprecision** | **Inconsistency** | | **Indirectness** | **Publication bias** | **Total** | **GRADE** |
| --- | --- | --- | --- | --- | --- | --- | --- | --- |
|  |  |  | **heterogeneity** | **incoherence** |  |  |  |  |
| Evolut vs Acurate | -1 | -1 | -0 | -0 | -0 | -0 | -2 | High |
| Evolut vs Portico | -1 | -1 | -0 | -0 | -0 | -0 | -2 | Moderate |
| Evolut vs Lotus | -1 | -1 | -0 | -0 | -0 | -0 | -2 | Low |
| Evolut vs DFM | -1 | -1 | -0 | -0 | -0 | -0 | -2 | Moderate |
| Evolut vs Sapien3 | -1 | -0 | -0 | -0 | -0 | -0 | -1 | Moderate |
| Acurate vs Portico | -1 | -1 | -0 | -0 | -0 | -0 | -2 | Moderate |
| Acurate vs Lotus | -1 | -1 | -0 | -0 | -0 | -0 | -2 | Low |
| Acurate vs Sapien3 | -1 | -1 | -0 | -0 | -0 | -0 | -2 | High |
| Portico vs Lotus | -1 | -1 | -0 | -0 | -0 | -0 | -2 | Moderate |
| Portico vs Sapien3 | -1 | -1 | -0 | -0 | -0 | -0 | -2 | Moderate |
| Lotus vs DFM | -1 | -1 | -0 | -0 | -0 | -0 | -2 | Low |
| Lotus vs Sapien3 | -1 | -1 | -0 | -0 | -0 | -0 | -2 | Low |
| DFM vs Sapien3 | -1 | -0 | -0 | -0 | -0 | -0 | -1 | High |
| Acurate vs DFM | -1 | -1 | -0 | -1 | -0 | -0 | -3 | Very low |
| Portico vs DFM | -1 | -0 | -0 | -1 | -0 | -0 | -2 | low |
| *Network* | *-1* | *-1* | *-0* | *-0* | *-0* | *-0* | *-2* | *Moderate* |

**(G) Permanent pacemaker implantation**

| **Comparison** | **Study limitations** | **Imprecision** | **Inconsistency** | | **Indirectness** | **Publication bias** | **Total** | **GRADE** |
| --- | --- | --- | --- | --- | --- | --- | --- | --- |
|  |  |  | **heterogeneity** | **incoherence** |  |  |  |  |
| Evolut vs Acurate | -1 | -0 | -0 | -0 | -0 | -0 | -1 | High |
| Evolut vs Portico | -1 | -0 | -0 | -0 | -0 | -0 | -1 | Moderate |
| Evolut vs Lotus | -1 | -0 | -0 | -0 | -0 | -0 | -1 | Moderate |
| Evolut vs DFM | -1 | -0 | -0 | -0 | -0 | -0 | -1 | Moderate |
| Evolut vs Sapien3 | -1 | -0 | -1 | -0 | -0 | -0 | -2 | Moderate |
| Acurate vs Portico | -1 | -0 | -0 | -0 | -0 | -0 | -1 | Moderate |
| Acurate vs Lotus | -1 | -0 | -0 | -0 | -0 | -0 | -1 | Moderate |
| Acurate vs Sapien3 | -1 | -0 | -1 | -1 | -0 | -0 | -3 | Low |
| Portico vs Lotus | -1 | -0 | -0 | -0 | -0 | -0 | -1 | Moderate |
| Portico vs DFM | -1 | -0 | -0 | -0 | -0 | -0 | -1 | Low |
| Portico vs Sapien3 | -1 | -0 | -1 | -0 | -0 | -0 | -2 | Low |
| Lotus vs DFM | -1 | -0 | -0 | -0 | -0 | -0 | -1 | Moderate |
| Lotus vs Sapien3 | -1 | -0 | -0 | -0 | -0 | -0 | -1 | Moderate |
| DFM vs Sapien3 | -1 | -0 | -0 | -0 | -0 | -0 | -1 | Moderate |
| Acurate vs DFM | -1 | -1 | -0 | -1 | -0 | -0 | -3 | Very low |
| *Network* | *-1* | *-0* | *-0* | *-1* | *-0* | *-0* | *-2* | *Moderate* |

**(H) Procedural mortality**

| **Comparison** | **Study limitations** | **Imprecision** | **Inconsistency** | | **Indirectness** | **Publication bias** | **Total** | **GRADE** |
| --- | --- | --- | --- | --- | --- | --- | --- | --- |
|  |  |  | **heterogeneity** | **incoherence** |  |  |  |  |
| Evolut vs Acurate | -1 | -1 | -0 | -0 | -0 | -0 | -2 | Moderate |
| Evolut vs Portico | -1 | -1 | -0 | -0 | -0 | -0 | -2 | Low |
| Evolut vs Lotus | -1 | -1 | -0 | -0 | -0 | -0 | -2 | Moderate |
| Evolut vs Sapien3 | -1 | -1 | -0 | -0 | -0 | -0 | -2 | Moderate |
| Acurate vs Portico | -1 | -0 | -0 | -1 | -0 | -0 | -2 | Low |
| Acurate vs Lotus | -0 | -1 | -0 | -0 | -0 | -0 | -1 | Moderate |
| Acurate vs Sapien3 | -0 | -1 | -0 | -0 | -0 | -0 | -1 | High |
| Portico vs Lotus | -1 | -0 | -1 | -0 | -0 | -0 | -2 | Low |
| Portico vs Sapien3 | -1 | -1 | -0 | -0 | -0 | -0 | -2 | Low |
| Lotus vs DFM | -0 | -1 | -0 | -0 | -0 | -0 | -1 | Moderate |
| Lotus vs Sapien3 | -0 | -1 | -0 | -0 | -0 | -0 | -1 | Moderate |
| DFM vs Sapien3 | -0 | -1 | -0 | -0 | -0 | -0 | -1 | Moderate |
| Evolut vs DFM | -1 | -1 | -0 | -1 | -0 | -0 | -3 | low |
| Acurate vs DFM | -0 | -1 | -0 | -1 | -0 | -0 | -2 | low |
| Portico vs DFM | -1 | -1 | -0 | -1 | -0 | -0 | -3 | Very low |
| *Network* | *-1* | *-0* | *-0* | *-0* | *-0* | *-0* | *-1* | *Moderate* |

**(I) No correct positioning**

| **Comparison** | **Study limitations** | **Imprecision** | **Inconsistency** | | **Indirectness** | **Publication bias** | **Total** | **GRADE** |
| --- | --- | --- | --- | --- | --- | --- | --- | --- |
|  |  |  | **heterogeneity** | **incoherence** |  |  |  |  |
| Evolut vs Acurate | -0 | -0 | -0 | -0 | -0 | -0 | -0 | Moderate |
| Evolut vs Portico | -1 | -1 | -0 | -0 | -0 | -0 | -2 | Low |
| Evolut vs Lotus | -0 | -0 | -0 | -0 | -0 | -0 | -0 | Moderate |
| Evolut vs Sapien3 | -0 | -0 | -0 | -0 | -0 | -0 | -0 | High |
| Acurate vs Portico | -1 | -1 | -0 | -0 | -0 | -0 | -2 | Low |
| Acurate vs Lotus | -0 | -1 | -0 | -1 | -0 | -0 | -2 | Low |
| Acurate vs Sapien3 | -0 | -0 | -1 | -0 | -0 | -0 | -1 | Moderate |
| Portico vs Lotus | -1 | -1 | -0 | -0 | -0 | -0 | -2 | Low |
| Portico vs Sapien3 | -1 | -0 | -0 | -0 | -0 | -0 | -1 | Moderate |
| Lotus vs DFM | -1 | -1 | -0 | -0 | -0 | -0 | -2 | Moderate |
| Lotus vs Sapien3 | -0 | -1 | -0 | -0 | -0 | -0 | -1 | Moderate |
| DFM vs Sapien3 | -1 | -1 | -0 | -0 | -0 | -0 | -2 | Moderate |
| Evolut vs DFM | -1 | -1 | -0 | -1 | -0 | -0 | -3 | low |
| Acurate vs DFM | -1 | -1 | -0 | -1 | -0 | -0 | -3 | low |
| Portico vs DFM | -1 | -1 | -0 | -1 | -0 | -0 | -3 | Very low |
| *Network* | *-1* | *-0* | *-0* | *-0* | *-0* | *-0* | *-1* | *Moderate* |

**(J) Moderate-to-severe paravalvular leak**

| **Comparison** | **Study limitations** | **Imprecision** | **Inconsistency** | | **Indirectness** | **Publication bias** | **Total** | **GRADE** |
| --- | --- | --- | --- | --- | --- | --- | --- | --- |
|  |  |  | **heterogeneity** | **incoherence** |  |  |  |  |
| Evolut vs Acurate | -1 | -0 | -0 | -0 | -0 | -0 | -1 | Moderate |
| Evolut vs Portico | -1 | -0 | -1 | -0 | -0 | -0 | -2 | Low |
| Evolut vs Lotus | -1 | -0 | -0 | -0 | -0 | -0 | -1 | Moderate |
| Evolut vs DFM | -1 | -1 | -0 | -0 | -0 | -0 | -2 | Low |
| Evolut vs Sapien3 | -1 | -0 | -1 | -0 | -0 | -0 | -2 | Low |
| Acurate vs Portico | -1 | -0 | -0 | -0 | -0 | -0 | -1 | Moderate |
| Acurate vs Lotus | -1 | -0 | -0 | -0 | -0 | -0 | -1 | Moderate |
| Acurate vs Sapien3 | -1 | -0 | -0 | -0 | -0 | -0 | -1 | Moderate |
| Portico vs Sapien3 | -1 | -0 | -0 | -0 | -0 | -0 | -1 | Moderate |
| Lotus vs DFM | -1 | -0 | -1 | -0 | -0 | -0 | -2 | Moderate |
| Lotus vs Sapien3 | -1 | -0 | -1 | -0 | -0 | -0 | -2 | Low |
| DFM vs Sapien3 | -1 | -1 | -0 | -0 | -0 | -0 | -2 | Low |
| Acurate vs DFM | -1 | -1 | -0 | -1 | -0 | -0 | -3 | Very low |
| Portico vs Lotus | -1 | -0 | -0 | -1 | -0 | -0 | -2 | low |
| Portico vs DFM | -1 | -1 | -0 | -1 | -0 | -0 | -3 | Very low |
| *Network* | *-1* | *-0* | *-0* | *-0* | *-0* | *-0* | *-1* | *Moderate* |

**(K) prosthesis patient mismatch**

| **Comparison** | **Study limitations** | **Imprecision** | **Inconsistency** | | **Indirectness** | **Publication bias** | **Total** | **GRADE** |
| --- | --- | --- | --- | --- | --- | --- | --- | --- |
|  |  |  | **heterogeneity** | **incoherence** |  |  |  |  |
| Evolut vs Acurate | -1 | -0 | -0 | -1 | -0 | -0 | -2 | Very low |
| Evolut vs Portico | -1 | -0 | -0 | -0 | -0 | -0 | -1 | Low |
| Evolut vs Lotus | -1 | -0 | -1 | -0 | -0 | -0 | -2 | Low |
| Evolut vs DFM | -1 | -1 | -0 | -0 | -0 | -0 | -2 | Moderate |
| Evolut vs Sapien3 | -1 | -0 | -0 | -0 | -0 | -0 | -1 | Low |
| Acurate vs Portico | -1 | -0 | -0 | -0 | -0 | -0 | -1 | Moderate |
| Acurate vs Lotus | -1 | -0 | -1 | -0 | -0 | -0 | -2 | Moderate |
| Acurate vs Sapien3 | -1 | -0 | -0 | -0 | -0 | -0 | -1 | Moderate |
| Portico vs Lotus | -1 | -1 | -0 | -0 | -0 | -0 | -2 | Very low |
| Portico vs Sapien3 | -1 | -0 | -1 | -0 | -0 | -0 | -2 | Low |
| Lotus vs DFM | -1 | -1 | -0 | -0 | -0 | -0 | -2 |  |
| Lotus vs Sapien3 | -1 | -1 | -0 | -0 | -0 | -0 | -2 | Moderate |
| DFM vs Sapien3 | -1 | -1 | -0 | -0 | -0 | -0 | -2 |  |
| Acurate vs DFM | -1 | -1 | -0 | -0 | -0 | -0 | -2 |  |
| Portico vs DFM | -1 | -1 | -0 | -0 | -0 | -0 | -2 |  |
| *Network* | *-1* | *-0* | *-0* | *-1* | *-0* | *-1* | *-3* |  |

**(L) Mean aortic valve gradients**

| **Comparison** | **Study limitations** | **Imprecision** | **Inconsistency** | | **Indirectness** | **Publication bias** | **Total** | **GRADE** |
| --- | --- | --- | --- | --- | --- | --- | --- | --- |
|  |  |  | **heterogeneity** | **incoherence** |  |  |  |  |
| Evolut vs Acurate | -1 | -0 | -1 | -1 | -0 | -0 | -3 | Moderate |
| Evolut vs Portico | -1 | -0 | -0 | -0 | -0 | -0 | -1 | Moderate |
| Evolut vs Lotus | -0 | -0 | -0 | -0 | -0 | -0 | -0 | High |
| Evolut vs Sapien3 | -0 | -0 | -0 | -0 | -0 | -0 | -0 | Moderate |
| Acurate vs Portico | -1 | -0 | -0 | -0 | -0 | -0 | -1 | Moderate |
| Acurate vs Lotus | -1 | -0 | -0 | -0 | -0 | -0 | -1 | Moderate |
| Acurate vs Sapien3 | -1 | -0 | -0 | -1 | -0 | -0 | -2 | Moderate |
| Portico vs Sapien3 | -1 | -0 | -0 | -0 | -0 | -0 | -1 | Moderate |
| Lotus vs DFM | -1 | -0 | -0 | -0 | -0 | -0 | -1 | Moderate |
| Lotus vs Sapien3 | -0 | -0 | -1 | -0 | -0 | -0 | -1 | High |
| DFM vs Sapien3 | -0 | -0 | -0 | -0 | -0 | -0 | -0 | High |
| Evolut vs DFM | -0 | -0 | -0 | -1 | -0 | -0 | -1 | Moderate |
| Acurate vs DFM | -0 | -0 | -0 | -1 | -0 | -0 | -1 | Moderate |
| Portico vs Lotus | -0 | -0 | -0 | -1 | -0 | -0 | -1 | Low |
| Portico vs DFM | -1 | -0 | -0 | -1 | -0 | -0 | -2 | Moderate |
| *Network* | *-1* | *-0* | *-1* | *-0* | *-0* | *-0* | *-2* | *Low* |

**Table 13. Sensitivity analyses: Effect estimates for each endpoint**

**(A) Device success**

| **Treatments** | **Comparisons** | **Network meta-analysis - OR (95% CI)** | | | |
| --- | --- | --- | --- | --- | --- |
|  |  | **Frequentist** | **Bayesian** | **30-day** | **No DFM** |
| Evolut | Acurate | 0.96 (0.71,1.29) | 0.94 (0.65, 1.44) | / | 0.96 (0.72,1.28) |
|  | Portico | 0.98 (0.64,1.49) | 0.94 (0.57, 1.65) | / | 0.98 (0.64,1.48) |
|  | Lotus | 1.05 (0.62,1.76) | 1.11 (0.60, 2.31) | / | 0.94 (0.56,1.58) |
|  | DFM | 2.29 (0.84,6.21) | 2.30 (0.85, 9.26) | / | / |
|  | Sapien 3 | 0.90 (0.69,1.18) | 0.94 (0.68, 1.33) | / | 0.91 (0.70,1.18) |
| Acurate | Portico | 1.02 (0.65,1.58) | 0.97 (0.57,1.77) | / | 1.02 (0.66,1.57) |
|  | Lotus | 1.09 (0.64,1.85) | 1.15 (0.61, 2.43) | / | 0.98 (0.57,1.66) |
|  | DFM | 2.38 (0.87,6.50) | 2.38 (0.87, 9.74) | / | / |
|  | Sapien 3 | 0.94 (0.71,1.25) | 0.97 (0.68, 1.44) | / | 0.95 (0.72,1.25) |
| Portico | Lotus | 1.07 (0.57,2.00) | 1.11 (0.55, 2.70) | / | 0.96 (0.52,1.79) |
|  | DFM | 2.35 (0.82,6.75) | 2.31 (0.81, 10.35) | / | / |
|  | Sapien 3 | 0.93 (0.60,1.44) | 0.94 (0.57, 1.71) | / | 0.93 (0.61,1.43) |
| Lotus | DFM | 2.19 (0.79,6.07) | 1.94 (0.70, 8.13) | / | / |
|  | Sapien 3 | 0.87 (0.54,1.37) | 0.78 (0.44, 1.47) | / | 0.97 (0.61,1.54) |
| DFM | Sapien 3 | 0.40 (0.15,1.04) | 0.29 (0.11, 1.07) | / | / |

**(B) Mortality**

| **Treatments** | **Comparisons** | **Network meta-analysis - OR (95% CI)** | | | |
| --- | --- | --- | --- | --- | --- |
|  |  | **Frequentist** | **Bayesian** | **30-day** | **No DFM** |
| Evolut | Acurate | 1.05 (0.79,1.39) | 1.05 (0.81, 1.42) | 0.89 (0.64,1.22) | 1.05 (0.79,1.40) |
|  | Portico | 0.96 (0.66,1.41) | 0.88 (0.62, 1.32) | 0.96 (0.61,1.51) | 0.95 (0.65,1.39) |
|  | Lotus | 0.81 (0.53,1.25) | 0.79 (0.54, 1.29) | 0.83 (0.52,1.31) | 0.83 (0.54,1.29) |
|  | DFM | 1.62 (0.58,4.58) | 1.66 (0.64, 6.28) | 1.64 (0.58,4.62) |  |
|  | Sapien 3 | 1.28 (1.18,1.40) | 1.28 (1.11, 1.48) | 1.29 (1.18,1.41) | 1.28 (1.18,1.40) |
| Acurate | Portico | 0.92 (0.59,1.42) | 0.83 (0.56, 1.27) | 1.08 (0.64,1.82) | 0.90 (0.58,1.40) |
|  | Lotus | 0.78 (0.47,1.27) | 0.74 (0.48, 1.26) | 0.93 (0.54,1.60) | 0.79 (0.48,1.30) |
|  | DFM | 1.55 (0.53,4.51) | 1.56 (0.60, 6.07) | 1.85 (0.63,5.44) |  |
|  | Sapien 3 | 1.23 (0.93,1.62) | 1.20 (0.91, 1.61) | 1.46 (1.06,2.00) | 1.22 (0.92,1.61) |
| Portico | Lotus | 0.85 (0.49,1.47) | 0.87 (0.53, 1.56) | 0.86 (0.46,1.61) | 0.88 (0.50,1.54) |
|  | DFM | 1.69 (0.56,5.07) | 1.83 (0.70,7.42) | 1.83 (0.70,7.42) |  |
|  | Sapien 3 | 1.34 (0.91,1.96) | 1.40 (0.98, 2.06) | 1.35 (0.86,2.12) | 1.35 (0.92,1.99) |
| Lotus | DFM | 1.99 (0.69,5.75) | 2.06 (0.85, 7.75) | 1.98 (0.68,5.74) | / |
|  | Sapien 3 | 1.58 (1.03,2.41) | 1.55 (1.00, 2.34) | 1.56 (0.99,2.45) | 1.54 (1.00,2.39) |
| DFM | Sapien 3 | 0.79 (0.28,2.22) | 0.55 (0.20, 1.98) | 0.79 (0.28,2.22) | / |

**(C) Stroke**

| **Treatments** | **Comparisons** | **Network meta-analysis - OR (95% CI)** | | | |
| --- | --- | --- | --- | --- | --- |
|  |  | **Frequentist** | **Bayesian** | **30-day** | **No DFM** |
| Evolut | Acurate | 1.46 (1.02,2.10) | 1.39 (0.92, 2.19) | 1.64 (1.07,2.51) | 1.46 (1.01,2.09) |
|  | Portico | 1.19 (0.70,2.02) | 1.37 (0.78, 2.67) | 1.75 (0.82,3.72) | 1.19 (0.69,2.04) |
|  | Lotus | 0.78 (0.48,1.26) | 0.67 (0.39, 1.23) | 0.79 (0.47,1.35) | 0.76 (0.46,1.25) |
|  | DFM | 3.11 (0.85,11.43) | 3.08 (0.96,22.14) | 3.16 (0.84,11.81) |  |
|  | Sapien 3 | 1.60 (1.21,2.12) | 1.46 (1.06, 2.03) | 1.61 (1.13,2.28) | 1.60 (1.21,2.12) |
| Acurate | Portico | 0.81 (0.46,1.43) | 0.95 (0.52, 2.01) | 1.07 (0.48,2.40) | 0.82 (0.46,1.45) |
|  | Lotus | 0.53 (0.31,0.90) | 0.46 (0.26, 0.91) | 0.48 (0.27,0.86) | 0.52 (0.31,0.89) |
|  | DFM | 2.13 (0.57,7.95) | 2.15 (0.66, 15.76) | 1.93 (0.51,7.35) |  |
|  | Sapien 3 | 1.09 (0.77,1.55) | 1.02 (0.69, 1.56) | 0.98 (0.66,1.47) | 1.10 (0.78,1.56) |
| Portico | Lotus | 0.65 (0.34,1.27) | 0.45 (0.22, 1.04) | 0.45 (0.20,1.04) | 0.64 (0.33,1.25) |
|  | DFM | 2.63 (0.66,10.40) | 2.06 (0.61, 16.13) | 1.80 (0.42,7.81) |  |
|  | Sapien 3 | 1.35 (0.80,2.29) | 0.98 (0.55, 1.88) | 0.92 (0.45,1.89) | 1.35 (0.79,2.30) |
| Lotus | DFM | 4.01 (1.13,14.20) | 4.47 (1.41, 30.59) | 3.98 (1.12,14.14) | / |
|  | Sapien 3 | 2.06 (1.34,3.17) | 2.06 (1.28, 3.54) | 2.03 (1.30,3.15) | 2.10 (1.36,3.25) |
| DFM | Sapien 3 | 0.51 (0.14,1.85) | 0.26 (0.07, 1.49) | 0.51 (0.14,1.83) | / |

**(D) Major/life threatening bleeding**

| **Treatments** | **Comparisons** | **Network meta-analysis - OR (95% CI)** | | | |
| --- | --- | --- | --- | --- | --- |
|  |  | **Frequentist** | **Bayesian** | **30-day** | **No DFM** |
| Evolut | Acurate | 0.90 (0.66,1.21) | 0.88 (0.66, 1.22) | 0.81 (0.53,1.23) | 0.90 (0.66,1.22) |
|  | Portico | 0.94 (0.64,1.38) | 0.91 (0.62, 1.41) | 0.92 (0.52,1.63) | 0.94 (0.64,1.39) |
|  | Lotus | 0.77 (0.50,1.18) | 0.75 (0.48, 1.21) | 0.80 (0.47,1.36) | 0.74 (0.48,1.15) |
|  | DFM | 1.12 (0.33,3.74) | 1.18 (0.42, 5.20) | 0.80 (0.19,3.39) |  |
|  | Sapien 3 | 0.84 (0.65,1.07) | 0.80 (0.62, 1.05) | 0.86 (0.58,1.26) | 0.84 (0.65,1.08) |
| Acurate | Portico | 1.05 (0.69,1.58) | 1.01 (0.67, 1.60) | 1.14 (0.64,2.02) | 1.05 (0.69,1.59) |
|  | Lotus | 0.85 (0.55,1.33) | 0.84 (0.54, 1.36) | 0.99 (0.59,1.65) | 0.83 (0.53,1.30) |
|  | DFM | 1.25 (0.37,4.19) | 1.33 (0.48, 5.90) | 0.99 (0.24,4.15) |  |
|  | Sapien 3 | 0.93 (0.72,1.21) | 0.90 (0.68, 1.19) | 1.06 (0.76,1.47) | 0.93 (0.72,1.21) |
| Portico | Lotus | 0.82 (0.49,1.36) | 0.80 (0.48, 1.42) | 0.87 (0.45,1.67) | 0.79 (0.47,1.33) |
|  | DFM | 1.19 (0.34,4.12) | 1.26 (0.44, 5.87) | 0.87 (0.20,3.86) |  |
|  | Sapien 3 | 0.89 (0.61,1.30) | 0.87 (0.58, 1.30) | 0.93 (0.55,1.58) | 0.89 (0.61,1.31) |
| Lotus | DFM | 1.46 (0.44,4.78) | 1.55 (0.56, 6.72) | 1.00 (0.25,3.97) | / |
|  | Sapien 3 | 1.09 (0.75,1.59) | 1.03 (0.70, 1.58) | 1.07 (0.71,1.61) | 1.12 (0.77,1.65) |
| DFM | Sapien 3 | 0.75 (0.23,2.45) | 0.45 (0.16, 1.86) | 1.07 (0.26,4.33) | / |

**(E) Major vascular complications**

| **Treatments** | **Comparisons** | **Network meta-analysis - OR (95% CI)** | | | |
| --- | --- | --- | --- | --- | --- |
|  |  | **Frequentist** | **Bayesian** | **30-day** | **No DFM** |
| Evolut | Acurate | 0.87 (0.64,1.19) | 0.89 (0.65, 1.29) | 0.65 (0.42,1.02) | 0.88 (0.64,1.20) |
|  | Portico | 1.05 (0.70,1.56) | 1.04 (0.69, 1.66) | 1.12 (0.64,1.94) | 1.05 (0.70,1.56) |
|  | Lotus | 1.07 (0.68,1.70) | 1.09 (0.67, 1.87) | 0.96 (0.57,1.62) | 1.04 (0.65,1.66) |
|  | DFM | 1.60 (0.47,5.44) | 1.76 (0.62, 7.26) | 1.05 (0.25,4.33) |  |
|  | Sapien 3 | 0.96 (0.72,1.27) | 0.94 (0.70, 1.29) | 0.79 (0.54,1.16) | 0.96 (0.72,1.28) |
| Acurate | Portico | 1.20 (0.79,1.82) | 1.15 (0.73, 1.86) | 1.71 (0.96,3.04) | 1.19 (0.78,1.82) |
|  | Lotus | 1.23 (0.77,1.95) | 1.20 (0.73, 2.06) | 1.47 (0.88,2.45) | 1.19 (0.74,1.91) |
|  | DFM | 1.83 (0.54,6.23) | 1.94 (0.67, 9.25) | 1.61 (0.39,6.60) |  |
|  | Sapien 3 | 1.10 (0.82,1.46) | 1.03 (0.75, 1.41) | 1.21 (0.87,1.68) | 1.10 (0.82,1.46) |
| Portico | Lotus | 1.03 (0.60,1.75) | 1.00 (0.58, 1.90) | 0.86 (0.46,1.61) | 1.00 (0.58,1.71) |
|  | DFM | 1.53 (0.43,5.37) | 1.62 (0.55, 8.16) | 0.94 (0.22,4.06) |  |
|  | Sapien 3 | 0.92 (0.61,1.37) | 0.86 (0.56, 1.39) | 0.70 (0.42,1.18) | 0.92 (0.61,1.38) |
| Lotus | DFM | 1.49 (0.45,4.97) | 1.58 (0.56, 7.23) | 1.09 (0.28,4.27) | / |
|  | Sapien 3 | 0.89 (0.60,1.33) | 0.82 (0.54, 1.32) | 0.82 (0.54,1.24) | 0.92 (0.61,1.39) |
| DFM | Sapien 3 | 0.60 (0.18,1.99) | 0.35 (0.12, 1.49) | 0.75 (0.19,2.99) | / |

**(F) Acute kidney injury**

| **Treatments** | **Comparisons** | **Network meta-analysis - OR (95% CI)** | | | |
| --- | --- | --- | --- | --- | --- |
|  |  | **Frequentist** | **Bayesian** | **30-day** | **No DFM** |
| Evolut | Acurate | 0.98 (0.72,1.33) | 1.00 (0.70, 1.51) | 0.73 (0.52,1.03) | 0.98 (0.72,1.34) |
|  | Portico | 1.16 (0.76,1.77) | 1.03 (0.62, 1.73) | 1.53 (0.93,2.51) | 1.16 (0.76,1.77) |
|  | Lotus | 0.99 (0.62,1.56) | 1.00 (0.61, 1.74) | 0.87 (0.54,1.40) | 0.98 (0.61,1.56) |
|  | DFM | 0.55 (0.24,1.27) | 0.54 (0.24,1.51) | 0.55 (0.24,1.29) |  |
|  | Sapien 3 | 1.02 (0.80,1.29) | 0.96 (0.71, 1.29) | 0.93 (0.71,1.21) | 1.02 (0.80,1.30) |
| Acurate | Portico | 1.18 (0.74,1.88) | 1.00 (0.58, 1.75) | 2.09 (1.21,3.61) | 1.17 (0.74,1.88) |
|  | Lotus | 1.00 (0.61,1.65) | 0.98 (0.57, 1.76) | 1.20 (0.71,2.02) | 0.99 (0.60,1.64) |
|  | DFM | 0.56 (0.24,1.32) | 0.52 (0.23, 1.48) | 0.76 (0.31,1.83) |  |
|  | Sapien 3 | 1.03 (0.78,1.37) | 0.94 (0.65, 1.33) | 1.27 (0.94,1.72) | 1.03 (0.77,1.38) |
| Portico | Lotus | 0.85 (0.48,1.51) | 0.93 (0.52, 1.89) | 0.57 (0.30,1.09) | 0.84 (0.47,1.51) |
|  | DFM | 0.47 (0.19,1.17) | 0.50 (0.21, 1.53) | 0.36 (0.14,0.95) |  |
|  | Sapien 3 | 0.88 (0.57,1.34) | 0.88 (0.56, 1.53) | 0.61 (0.37,1.01) | 0.88 (0.57,1.35) |
| Lotus | DFM | 0.55 (0.24,1.26) | 0.51 (0.23, 1.42) | 0.63 (0.28,1.43) | / |
|  | Sapien 3 | 1.03 (0.66,1.61) | 0.90 (0.57, 1.52) | 1.06 (0.67,1.68) | 1.04 (0.66,1.65) |
| DFM | Sapien 3 | 1.85 (0.81,4.26) | 1.47 (0.67, 3.91) | 1.68 (0.72,3.89) | / |

**(G) Permanent pacemaker implantation**

| **Treatments** | **Comparisons** | **Network meta-analysis - OR (95% CI)** | | | |
| --- | --- | --- | --- | --- | --- |
|  |  | **Frequentist** | **Bayesian** | **30-day** | **No DFM** |
| Evolut | Acurate | 2.18 (1.81,2.63) | 2.22 (1.83, 2.71) | 2.25 (1.73,2.92) | 2.20 (1.82,2.65) |
|  | Portico | 1.01 (0.82,1.24) | 1.00 (0.81, 1.25) | 0.98 (0.68,1.40) | 1.01 (0.82,1.25) |
|  | Lotus | 0.52 (0.42,0.65) | 0.51 (0.41, 0.65) | 0.53 (0.41,0.69) | 0.53 (0.42,0.68) |
|  | DFM | 2.10 (1.36,3.23) | 2.13 (1.41, 3.46) | 2.35 (1.41,3.91) |  |
|  | Sapien 3 | 1.56 (1.38,1.76) | 1.57 (1.39, 1.81) | 1.58 (1.32,1.89) | 1.57 (1.38,1.78) |
| Acurate | Portico | 0.46 (0.36,0.58) | 0.45 (0.35, 0.58) | 0.43 (0.29,0.65) | 0.46 (0.36,0.59) |
|  | Lotus | 0.24 (0.18,0.31) | 0.23 (0.18, 0.30) | 0.24 (0.17,0.32) | 0.24 (0.19,0.32) |
|  | DFM | 0.96 (0.61,1.51) | 0.96 (0.62, 1.59) | 1.04 (0.61,1.79) |  |
|  | Sapien 3 | 0.71 (0.60,0.85) | 0.71 (0.59, 0.86) | 0.70 (0.56,0.89) | 0.71 (0.60,0.86) |
| Portico | Lotus | 0.52 (0.39,0.68) | 0.51 (0.38, 0.69) | 0.54 (0.36,0.80) | 0.53 (0.39,0.70) |
|  | DFM | 2.08 (1.31,3.31) | 2.11 (1.36, 3.54) | 2.41 (1.34,4.32) |  |
|  | Sapien 3 | 1.55 (1.26,1.91) | 1.56 (1.26, 1.95) | 1.62 (1.14,2.30) | 1.55 (1.25,1.92) |
| Lotus | DFM | 4.02 (2.59,6.23) | 4.12 (2.69, 6.76) | 4.44 (2.68,7.36) | / |
|  | Sapien 3 | 2.99 (2.44,3.66) | 3.03 (2.47, 3.78) | 2.99 (2.39,3.74) | 2.95 (2.37,3.66) |
| DFM | Sapien 3 | 0.74 (0.49,1.13) | 0.70 (0.46, 1.11) | 0.67 (0.41,1.10) | / |

**(H) Procedural mortality**

| **Treatments** | **Comparisons** | **Network meta-analysis - OR (95% CI)** | | | |
| --- | --- | --- | --- | --- | --- |
|  |  | **Frequentist** | **Bayesian** | **30-day** | **No DFM** |
| Evolut | Acurate | 0.73 (0.30,1.79) | 0.80 (0.25, 4.95) | / | 0.73 (0.30,1.80) |
|  | Portico | 3.23 (0.73,14.30) | 4.28 (0.82, 395.26) | / | 3.21 (0.72,14.19) |
|  | Lotus | 0.58 (0.19,1.74) | 0.25 (0.07, 8.06) | / | 0.54 (0.18,1.64) |
|  | DFM | 0.85 (0.08,9.33) | 0.03 (0.01, 120.70) | / |  |
|  | Sapien 3 | 1.21 (0.65,2.24) | 1.32 (0.49, 5.71) | / | 1.21 (0.65,2.25) |
| Acurate | Portico | 4.41 (0.88,22.14) | 3.83 (0.71, 386.70) | / | 4.37 (0.87,21.96) |
|  | Lotus | 0.79 (0.24,2.64) | 0.32 (0.06, 7.40) | / | 0.74 (0.22,2.49) |
|  | DFM | 1.16 (0.10,13.35) | 0.02 (0.01, 116.55) | / |  |
|  | Sapien 3 | 1.65 (0.76,3.58) | 1.23 (0.42, 5.38) | / | 1.65 (0.76,3.60) |
| Portico | Lotus | 0.18 (0.03,0.97) | 0.01 (0.00, 1.52) | / | 0.17 (0.03,0.92) |
|  | DFM | 0.26 (0.02,4.05) | 0.00 (0.00, 18.46) | / |  |
|  | Sapien 3 | 0.37 (0.08,1.64) | 0.02 (0.00, 1.94) | / | 0.38 (0.09,1.66) |
| Lotus | DFM | 1.46 (0.14,14.97) | 0.04 (0.02, 136.20) | / | / |
|  | Sapien 3 | 2.07 (0.79,5.45) | 1.21 (0.25, 19.07) | / | 2.25 (0.84,6.04) |
| DFM | Sapien 3 | 1.42 (0.14,14.47) | 0.07 (0.01, 115.79) | / | / |

**(I) No correct positioning**

| **Treatments** | **Comparisons** | **Network meta-analysis - OR (95% CI)** | | | |
| --- | --- | --- | --- | --- | --- |
|  |  | **Frequentist** | **Bayesian** | **30-day** | **No DFM** |
| Evolut | Acurate | 1.69 (0.94,3.01) | 2.29 (1.14, 5.94) | / | 1.68 (0.94,3.00) |
|  | Portico | 0.97 (0.50,1.91) | 1.23 (0.52, 3.88) | / | 0.97 (0.50,1.91) |
|  | Lotus | 3.03 (0.81,11.27) | 7.31 (1.51, 482.86) | / | 4.02 (1.02,15.88) |
|  | DFM | 2.77 (0.43,17.79) | 2.23 (0.43, 164.04) | / |  |
|  | Sapien 3 | 3.67 (2.12,6.36) | 5.98 (2.97, 17.79) | / | 3.62 (2.09,6.27) |
| Acurate | Portico | 0.58 (0.26,1.29) | 0.48 (0.18, 1.66) | / | 0.58 (0.26,1.29) |
|  | Lotus | 1.80 (0.48,6.78) | 2.90 (0.58, 197.39) | / | 2.39 (0.60,9.60) |
|  | DFM | 1.65 (0.26,10.58) | 0.92 (0.17,61.54) | / |  |
|  | Sapien 3 | 2.18 (1.24,3.83) | 2.42 (1.20, 6.62) | / | 2.15 (1.22,3.79) |
| Portico | Lotus | 3.11 (0.75,12.97) | 4.91 (0.96, 376.36) | / | 4.13 (0.94,18.18) |
|  | DFM | 2.85 (0.41,19.83) | 1.48 (0.29, 125.05) | / |  |
|  | Sapien 3 | 3.76 (1.71,8.28) | 3.99 (1.57, 16.48) | / | 3.72 (1.69,8.15) |
| Lotus | DFM | 0.92 (0.13,6.53) | 0.06 (0.01, 12.28） | / | / |
|  | Sapien 3 | 1.21 (0.35,4.21) | 0.05 (0.01, 4.29) | / | 0.90 (0.24,3.36) |
| DFM | Sapien 3 | 1.32 (0.22,7.86) | 0.28 (0.05, 13.64) | / | / |

**(J) Moderate-to-severe paravalvular leak**

| **Treatments** | **Comparisons** | **Network meta-analysis - OR (95% CI)** | | | |
| --- | --- | --- | --- | --- | --- |
|  |  | **Frequentist** | **Bayesian** | **30-day** | **No DFM** |
| Evolut | Acurate | 0.76 (0.57,1.00) | 0.75 (0.55, 1.05) | / | 0.77 (0.58,1.01) |
|  | Portico | 0.59 (0.41,0.84) | 0.57 (0.39, 0.89) | / | 0.59 (0.41,0.84) |
|  | Lotus | 3.76 (2.22,6.39) | 4.77 (2.72, 9.44) | / | 4.00 (2.35,6.82) |
|  | DFM | 1.12 (0.43,2.93) | 1.00 (0.41, 3.24) |  |  |
|  | Sapien 3 | 1.90 (1.44,2.50) | 2.01 (1.50, 2.78) | / | 1.94 (1.47,2.56) |
| Acurate | Portico | 0.77 (0.53,1.14) | 0.75 (0.50, 1.20) | / | 0.77 (0.53,1.13) |
|  | Lotus | 4.96 (2.88,8.57) | 6.30 (3.53, 12.71) | / | 5.21 (3.01,9.02) |
|  | DFM | 1.48 (0.56,3.92) | 1.31 (0.53, 4.35) | / |  |
|  | Sapien 3 | 2.50 (1.89,3.32) | 2.65 (1.96, 3.70) | / | 2.53 (1.91,3.35) |
| Portico | Lotus | 6.41 (3.47,11.83) | 8.03 (4.23, 17.66) | / | 6.77 (3.66,12.52) |
|  | DFM | 1.91 (0.70,5.26) | 1.68 (0.65, 5.88) | / |  |
|  | Sapien 3 | 3.23 (2.16,4.84) | 3.38 (2.22, 5.48) | / | 3.28 (2.19,4.90) |
| Lotus | DFM | 0.30 (0.11,0.84) | 0.19 (0.07, 0.72) | / | / |
|  | Sapien 3 | 0.50 (0.30,0.86) | 0.39 (0.22, 0.75) | / | 0.48 (0.28,0.82) |
| DFM | Sapien 3 | 1.69 (0.65,4.38) | 1.55 (0.64, 4.89) | / | / |

**(K) Prosthesis patient mismatch**

| **Treatments** | **Comparisons** | **Network meta-analysis - OR (95% CI)** | | | |
| --- | --- | --- | --- | --- | --- |
|  |  | **Frequentist** | **Bayesian** | **30-day** | **No DFM** |
| Evolut | Acurate | 0.85 (0.64,1.12) | 0.81 (0.58, 1.15) | / | 0.84 (0.63,1.13) |
|  | Portico | 0.69 (0.47,1.01) | 0.66 (0.43, 1.06) | / | 0.69 (0.46,1.01) |
|  | Lotus | 0.49 (0.31,0.76) | 0.45 (0.28, 0.76) | / | 0.47 (0.29,0.75) |
|  | DFM | 0.49 (0.18,1.34) | 0.40 (0.16, 1.40) | / |  |
|  | Sapien 3 | 0.39 (0.31,0.51) | 0.36 (0.27, 0.48) | / | 0.39 (0.30,0.51) |
| Acurate | Portico | 0.81 (0.55,1.20) | 0.80 (0.52, 1.31) | / | 0.81 (0.55,1.21) |
|  | Lotus | 0.57 (0.36,0.91) | 0.55 (0.34, 0.96) | / | 0.55 (0.34,0.91) |
|  | DFM | 0.57 (0.20,1.61) | 0.48 (0.19, 1.75) | / |  |
|  | Sapien 3 | 0.47 (0.35,0.62) | 0.44 (0.31, 0.63) | / | 0.47 (0.34,0.63) |
| Portico | Lotus | 0.71 (0.41,1.21) | 0.66 (0.37,1.26) | / | 0.68 (0.38,1.20) |
|  | DFM | 0.71 (0.24,2.06) | 0.58 (0.22, 2.21) | / |  |
|  | Sapien 3 | 0.57 (0.38,0.86) | 0.53 (0.33, 0.86) | / | 0.57 (0.37,0.87) |
| Lotus | DFM | 1.00 (0.35,2.81) | 0.85 (0.33, 3.06) | / | / |
|  | Sapien 3 | 0.81 (0.54,1.21) | 0.77 (0.50, 1.22) | / | 0.84 (0.54,1.30) |
| DFM | Sapien 3 | 0.81 (0.30,2.22) | 0.67 (0.26, 2.31) | / | / |

**(L) Mean aortic valve gradients**

| **Treatments** | **Comparisons** | **Network meta-analysis - OR (95% CI)** | | | |
| --- | --- | --- | --- | --- | --- |
|  |  | **Frequentist** | **Bayesian** | **30-day** | **No DFM** |
| Evolut | Acurate | -0.87 (-1.53, -0.21) | -0.88 (-1.56, -0.20) | / | -0.86 (-1.46, -0.26) |
|  | Portico | -0.53 (-1.27, 0.20) | -0.53 (-1.30, 0.24) | / | -0.55 (-1.22, 0.11) |
|  | Lotus | -4.78 (-5.78, -3.78) | -4.78 (-5.83, -3.75) | / | -4.74 (-5.70, -3.78) |
|  | DFM | -8.18 (-9.58, -6.79) | -8.20 (-9.66, -6.79) |  |  |
|  | Sapien 3 | -3.66 (-4.17, -3.16) | -3.67 (-4.19, -3.15) | / | -3.66 (-4.11, -3.20) |
| Acurate | Portico | 0.34 (-0.50,1.18) | 0.34 (-0.52, 1.22) | / | 0.31 (-0.45, 1.07) |
|  | Lotus | -3.91 (-4.97, -2.85) | -3.91 (-5.00, -2.81) | / | -3.88 (-4.88, -2.87) |
|  | DFM | -7.31 (-8.75, -5.87) | -7.32 (-8.83, -5.87) | / |  |
|  | Sapien 3 | -2.79 (-3.43, -2.16) | -2.79 (-3.45, -2.13) | / | -2.80 (-3.37, -2.22) |
| Portico | Lotus | -4.25 (-5.41, -3.08) | -4.25 (-5.46, -3.06) | / | -4.18 (-5.28, -3.09) |
|  | DFM | -7.65 (-9.17, -6.13) | -7.66 (-9.27, -6.13) | / |  |
|  | Sapien 3 | -3.13 (-3.93, -2.33) | -3.13 (-3.97, -2.32) | / | -3.10 (-3.83, -2.38) |
| Lotus | DFM | -3.40 (-4.85, -1.96) | -3.42 (-4.93, -1.95) | / | / |
|  | Sapien 3 | 1.12 (0.20, 2.04) | 1.11 (0.16, 2.07) | / | 1.08 (0.18, 1.98) |
| DFM | Sapien 3 | 4.52 (3.22, 5.82) | 4.53 (3.21, 5.90) | / | / |

**Table 14. Sensitivity analyses: Surface Under the Cumulative Ranking (SUCRA) values and mean rank for each endpoint**

**(A) Device success**

| **Frequentist** | |  | **Bayesian** | |  | **30-day** | |  | **No DFM** | |
| --- | --- | --- | --- | --- | --- | --- | --- | --- | --- | --- |
| **Treatment** | **SUCRA (Mean rank)** |  | **Treatment** | **SUCRA (Mean rank)** |  | **Treatment** | **SUCRA (Mean rank)** |  | **Treatment** | **SUCRA (Mean rank)** |
| DFM | 4.6 (5.8) |  | DFM | 5.0 (5.8) |  | / | / |  | Evolut | 37.1 (3.5) |
| Lotus | 48.8 (3.6) |  | Lotus | 41.8 (3.9) |  | / | / |  | Portico | 46.4 (3.1) |
| Evolut | 51.8 (3.4) |  | Evolut | 58.2 (3.1) |  | / | / |  | Acurate | 48.7 (3.1) |
| Portico | 58.3 (3.1) |  | Portico | 62.4 (2.9) |  | / | / |  | Lotus | 52.7 (2.9) |
| Acurate | 61.3 (2.9) |  | Acurate | 65.2 (2.7) |  | / | / |  | Sapien 3 | 65.1 (2.4) |
| Sapien 3 | 75.3 (2.2) |  | Sapien 3 | 67.5 (2.6) |  | / | / |  | / | / |

**(B) Mortality**

| **Frequentist** | |  | **Bayesian** | |  | **30-day** | |  | **No DFM** | |
| --- | --- | --- | --- | --- | --- | --- | --- | --- | --- | --- |
| **Treatment** | **SUCRA (Mean rank)** |  | **Treatment** | **SUCRA (Mean rank)** |  | **Treatment** | **SUCRA (Mean rank)** |  | **Treatment** | **SUCRA (Mean rank)** |
| Sapien 3 | 16.8 (5.2) |  | DFM | 13.8 (5.3) |  | Sapien 3 | 15.5 (5.2) |  | Sapien 3 | 4.3 (4.8) |
| DFM | 19.7 (5.0) |  | Sapien 3 | 18.2 (5.1) |  | DFM | 19.0 (5.0) |  | Acurate | 44.2 (3.2) |
| Acurate | 52.0 (3.4) |  | Acurate | 49.4 (3.5) |  | Evolut | 55.9 (3.2) |  | Evolut | 56.2 (2.8) |
| Evolut | 60.7 (3.0) |  | Evolut | 59.7 (3.0) |  | Acurate | 58.6 (3.1) |  | Portico | 63.6 (2.5) |
| Portico | 65.7 (2.7) |  | Portico | 73.9 (2.3) |  | Portico | 68.3 (2.6) |  | Lotus | 81.6 (1.7) |
| Lotus | 85.1 (1.7) |  | Lotus | 85.1 (1.7) |  | Lotus | 82.8 (1.9) |  | / | / |

**(C) Stroke**

| **Frequentist** | |  | **Bayesian** | |  | **30-day** | |  | **No DFM** | |
| --- | --- | --- | --- | --- | --- | --- | --- | --- | --- | --- |
| **Treatment** | **SUCRA (Mean rank)** |  | **Treatment** | **SUCRA (Mean rank)** |  | **Treatment** | **SUCRA (Mean rank)** |  | **Treatment** | **SUCRA (Mean rank)** |
| DFM | 8.6 (5.6) |  | DFM | 5.6 (5.7) |  | DFM | 12.1 (5.4) |  | Sapien 3 | 10.9 (4.6) |
| Sapien 3 | 25.5 (4.7) |  | Sapien 3 | 36.4 (4.2) |  | Acurate | 35.1 (4.2) |  | Acurate | 24.4 (4.0) |
| Acurate | 36.5 (4.2) |  | Acurate | 41.4 (3.9) |  | Portico | 35.6 (4.2) |  | Portico | 49.5 (3.0) |
| Portico | 58.7 (3.1) |  | Portico | 41.6 (3.9) |  | Sapien 3 | 41.4 (3.9) |  | Evolut | 71.3 (2.1) |
| Evolut | 76.7 (2.2) |  | Evolut | 77.9 (2.1) |  | Evolut | 80.2 (2.0) |  | Lotus | 93.9 (1.2) |
| Lotus | 94.1 (1.3) |  | Lotus | 96.9 (1.2) |  | Lotus | 95.6 (1.2) |  | / | / |

**(D)** **Major/life threatening bleeding**

| **Frequentist** | |  | **Bayesian** | |  | **30-day** | |  | **No DFM** | |
| --- | --- | --- | --- | --- | --- | --- | --- | --- | --- | --- |
| **Treatment** | **SUCRA (Mean rank)** |  | **Treatment** | **SUCRA (Mean rank)** |  | **Treatment** | **SUCRA (Mean rank)** |  | **Treatment** | **SUCRA (Mean rank)** |
| Evolut | 27.6 (4.6) |  | DFM | 21.9 (4.9) |  | Evolut | 12.3 (5.4) |  | Evolut | 19.5 (4.2) |
| DFM | 35.8 (4.2) |  | Evolut | 29.6 (4.5) |  | Sapien 3 | 48.1 (3.6) |  | Portico | 38.1 (3.5) |
| Portico | 42.7 (3.9) |  | Portico | 46.7 (3.7) |  | Portico | 52.6 (3.4) |  | Acurate | 46.4 (3.1) |
| Acurate | 50.6 (3.5) |  | Acurate | 52.6 (3.4) |  | DFM | 55.8 (3.2) |  | Sapien 3 | 65.2 (2.4) |
| Sapien 3 | 67.1 (2.6) |  | Sapien 3 | 73.2 (2.3) |  | Acurate | 64.9 (2.8) |  | Lotus | 80.9 (1.8) |
| Lotus | 76.3 (2.2) |  | Lotus | 76.0 (2.2) |  | Lotus | 66.3 (2.7) |  | / | / |

**(E) Major vascular complications**

| **Frequentist** | |  | **Bayesian** | |  | **30-day** | |  | **No DFM** | |
| --- | --- | --- | --- | --- | --- | --- | --- | --- | --- | --- |
| **Treatment** | **SUCRA (Mean rank)** |  | **Treatment** | **SUCRA (Mean rank)** |  | **Treatment** | **SUCRA (Mean rank)** |  | **Treatment** | **SUCRA (Mean rank)** |
| DFM | 22.4 (4.9) |  | DFM | 12.1 (5.4) |  | Evolut | 27.0 (4.7) |  | Portico | 35.9 (3.6) |
| Lotus | 40.8 (4.0) |  | Lotus | 40.1 (4.0) |  | Portico | 39.8 (4.0) |  | Lotus | 38.7 (3.5) |
| Portico | 44.8 (3.8) |  | Portico | 46.2 (3.7) |  | DFM | 40.5 (4.0) |  | Evolut | 48.7 (3.3) |
| Evolut | 51.3 (3.4) |  | Evolut | 56.4 (3.2) |  | Lotus | 43.9 (3.8) |  | Sapien 3 | 54.8 (2.8) |
| Sapien 3 | 61.1 (2.9) |  | Sapien 3 | 68.2 (2.6) |  | Sapien 3 | 58.7 (3.1) |  | Acurate | 76.9 (1.9) |
| Acurate | 79.6 (2.0) |  | Acurate | 76.4 (2.2) |  | Acurate | 90.1 (1.5) |  | / | / |

**(F) Acute kidney injury**

| **Frequentist** | |  | **Bayesian** | |  | **30-day** | |  | **No DFM** | |
| --- | --- | --- | --- | --- | --- | --- | --- | --- | --- | --- |
| **Treatment** | **SUCRA (Mean rank)** |  | **Treatment** | **SUCRA (Mean rank)** |  | **Treatment** | **SUCRA (Mean rank)** |  | **Treatment** | **SUCRA (Mean rank)** |
| Portico | 22.6 (4.9) |  | Portico | 34.9 (4.3) |  | Portico | 2.8 (5.9) |  | Portico | 26.6 (3.9) |
| Sapien 3 | 41.9 (3.9) |  | Lotus | 38.4 (4.1) |  | Evolut | 35.5 (4.2) |  | Sapien 3 | 50.1 (3.0) |
| Evolut | 46.5 (3.7) |  | Acurate | 43.6 (3.9) |  | Sapien 3 | 45.2 (3.7) |  | Evolut | 56.0 (2.8) |
| Lotus | 47.0 (3.7) |  | Evolut | 44.9 (3.8) |  | Lotus | 54.0 (3.3) |  | Lotus | 58.3 (2.7) |
| Acurate | 50.1 (3.5) |  | Sapien 3 | 51.9 (3.4) |  | Acurate | 74.3 (2.3) |  | Acurate | 59.0 (2.6) |
| DFM | 92.1 (1.4) |  | DFM | 87.3 (1.6) |  | DFM | 88.3 (1.6) |  | / | / |

**(G) Permanent pacemaker implantation**

| **Frequentist** | |  | **Bayesian** | |  | **30-day** | |  | **No DFM** | |
| --- | --- | --- | --- | --- | --- | --- | --- | --- | --- | --- |
| **Treatment** | **SUCRA (Mean rank)** |  | **Treatment** | **SUCRA (Mean rank)** |  | **Treatment** | **SUCRA (Mean rank)** |  | **Treatment** | **SUCRA (Mean rank)** |
| Acurate | 8.6 (5.6) |  | Acurate | 9.4 (5.5) |  | DFM | 10.0 (5.5) |  | Acurate | 0.0 (5.0) |
| DFM | 13.2 (5.3) |  | DFM | 12.0 (5.4) |  | Acurate | 11.1 (5.4) |  | Sapien 3 | 25.0 (4.0) |
| Sapien 3 | 38.3 (4.1) |  | Sapien 3 | 38.7 (4.1) |  | Sapien 3 | 39.0 (4.1) |  | Portico | 61.4 (2.5) |
| Portico | 69.6 (2.5) |  | Portico | 69.5 (2.5) |  | Evolut | 69.3 (2.5) |  | Evolut | 63.6 (2.5) |
| Evolut | 70.3 (2.5) |  | Evolut | 70.5 (2.5) |  | Portico | 70.6 (2.4) |  | Lotus | 100.0 (1.0) |
| Lotus | 100.0 (1.0) |  | Lotus | 100.0 (1.0) |  | Lotus | 100.0 (1.0) |  | / | / |

**(H) Procedural mortality**

| **Frequentist** | |  | **Bayesian** | |  | **No DFM** | |
| --- | --- | --- | --- | --- | --- | --- | --- |
| **Treatment** | **SUCRA (Mean rank)** |  | **Treatment** | **SUCRA (Mean rank)** |  | **Treatment** | **SUCRA (Mean rank)** |
| Portico | 7.7 (5.6) |  | Portico | 7.9 (5.6） |  | Portico | 5.7 (4.8) |
| Sapien 3 | 34.5 (4.3) |  | Sapien 3 | 40.2 (4.0) |  | Sapien 3 | 33.4 (3.7) |
| Evolut | 50.4 (3.5) |  | DFM | 55.7 (3.2) |  | Evolut | 51.1 (3.0) |
| DFM | 57.0 (3.2) |  | Acurate | 60.9 (3.0) |  | Acurate | 73.0 (2.1) |
| Acurate | 70.0 (2.5) |  | Evolut | 62.5 (2.9) |  | Lotus | 86.9 (1.5) |
| Lotus | 80.3 (2.0) |  | Lotus | 72.8 (2.4) |  | / | / |

**(I) No correct positioning**

| **Frequentist** | |  | **Bayesian** | |  | **No DFM** | |
| --- | --- | --- | --- | --- | --- | --- | --- |
| **Treatment** | **SUCRA (Mean rank)** |  | **Treatment** | **SUCRA (Mean rank)** |  | **Treatment** | **SUCRA (Mean rank)** |
| Sapien 3 | 15.3 (5.2) |  | Lotus | 14.0 (5.3) |  | Sapien 3 | 14.3 (4.4) |
| Lotus | 27.7 (4.6) |  | Sapien 3 | 25.4 (4.7) |  | Lotus | 14.7 (4.4) |
| DFM | 34.9 (4.3) |  | DFM | 31.8 (4.4) |  | Acurate | 50.6 (3.0) |
| Acurate | 52.6 (3.4) |  | Acurate | 56.9 (2.8) |  | Portico | 85.1 (1.6) |
| Evolut | 84.6 (1.8) |  | Portico | 78.9 (2.1) |  | Evolut | 85.3 (1.6) |
| Portico | 85.0 (1.8) |  | Evolut | 93.1 (1.5) |  | / | / |

**(J) Moderate-to-severe paravalvular leak**

| **Frequentist** | |  | **Bayesian** | |  | **No DFM** | |
| --- | --- | --- | --- | --- | --- | --- | --- |
| **Treatment** | **SUCRA (Mean rank)** |  | **Treatment** | **SUCRA (Mean rank)** |  | **Treatment** | **SUCRA (Mean rank)** |
| Lotus | 0.3 (6.0) |  | Lotus | 0.0 (6.0) |  | Lotus | 0.1 (5.0) |
| Sapien 3 | 22.7 (4.9) |  | Sapien 3 | 22.6 (4.9) |  | Sapien 3 | 24.9 (4.0) |
| Evolut | 52.2 (3.4) |  | DFM | 51.8 (3.4) |  | Evolut | 50.8 (3.0) |
| DFM | 51.7 (3.4) |  | Evolut | 53.1 (3.3) |  | Acurate | 76.5 (1.9) |
| Acurate | 76.9 (2.2) |  | Acurate | 77.2 (2.1) |  | Portico | 97.7 (1.1) |
| Portico | 96.2 (1.2) |  | Portico | 95.1 (1.2) |  | / | / |

**(K) Prosthesis patient mismatch**

| **Frequentist** | |  | **Bayesian** | |  | **No DFM** | |
| --- | --- | --- | --- | --- | --- | --- | --- |
| **Treatment** | **SUCRA (Mean rank)** |  | **Treatment** | **SUCRA (Mean rank)** |  | **Treatment** | **SUCRA (Mean rank)** |
| Evolut | 4.7 (5.8) |  | Evolut | 4.8 (5.8) |  | Evolut | 3.8 (4.8) |
| Acurate | 23.5 (4.8) |  | Acurate | 25.0 (4.7) |  | Acurate | 26.2 (4.0) |
| Portico | 43.8 (3.8) |  | Portico | 42.8 (3.9) |  | Portico | 47.7 (3.1) |
| DFM | 67.0 (2.7) |  | DFM | 66.7 (2.7) |  | Lotus | 77.8 (1.9) |
| Lotus | 70.6 (2.5) |  | Lotus | 70.2 (2.5) |  | Sapien 3 | 94.6 (1.2) |
| Sapien 3 | 90.3 (1.5) |  | Sapien 3 | 90.5 (1.5) |  | / | / |

**(L) Mean aortic valve gradients**

| **Frequentist** | |  | **Bayesian** | |  | **No DFM** | |
| --- | --- | --- | --- | --- | --- | --- | --- |
| **Treatment** | **SUCRA (Mean rank)** |  | **Treatment** | **SUCRA (Mean rank)** |  | **Treatment** | **SUCRA (Mean rank)** |
| Evolut | 1.5 (5.9) |  | Evolut | 1.8 (5.9) |  | Evolut | 1.4 (4.9) |
| Portico | 23.0 (4.8) |  | Portico | 22.6 (4.8) |  | Portico | 29.1 (3.8) |
| Acurate | 35.5 (4.2) |  | Acurate | 35.5 (4.4) |  | Acurate | 44.5 (3.2) |
| Sapien 3 | 60.2 (3.0) |  | Sapien 3 | 60.2 (3.0) |  | Sapien 3 | 75.2 (2.0) |
| Lotus | 79.8 (2.0) |  | Lotus | 79.8 (2.1) |  | Lotus | 99.8 (1.0) |
| DFM | 100.0 (1.0) |  | DFM | 100.0（1.0） |  | / | / |

**Table 15. Assessment of Bayesian random effects model fit**

| **Endpoints** | **Model fit** | |
| --- | --- | --- |
|  | **Data points** | **Total residual variance** |
| Device success | 96 | 99.32 |
| Mortality | 135 | 128.6 |
| Stroke | 116 | 113.0 |
| Major/life threatening bleeding | 110 | 103.3 |
| Major vascular complications | 106 | 106.4 |
| Acute kidney injury | 89 | 99.43 |
| Permanent pacemaker implantation | 161 | 159.2 |
| Procedural mortality | 54 | 47.86 |
| No correct positioning | 81 | 74.86 |
| Moderate-to-severe paravalvular leak | 125 | 123.3 |
| Prosthesis patient mismatch | 47 | 50.35 |
| Mean aortic valve gradients | 94 | 93.1 |

It means a good model fit if the total residual deviance approximated the number of data points

**Figure 1. Assessment of the transitivity assumption**

| 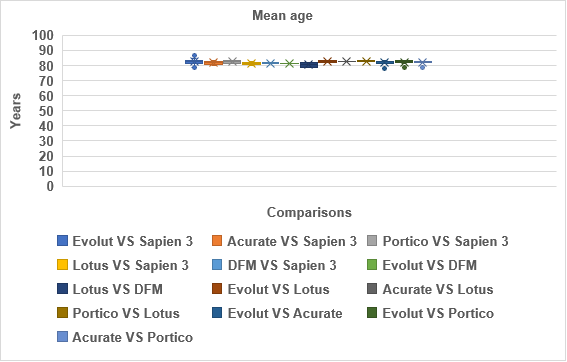 | 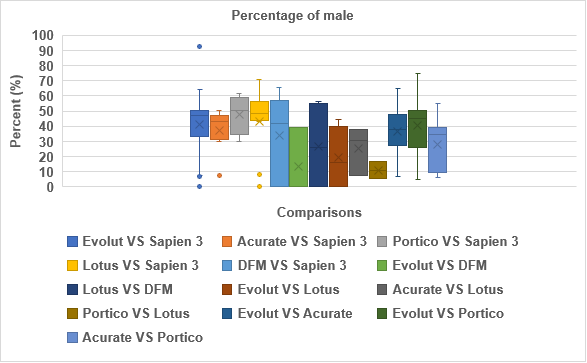 |
| --- | --- |
| 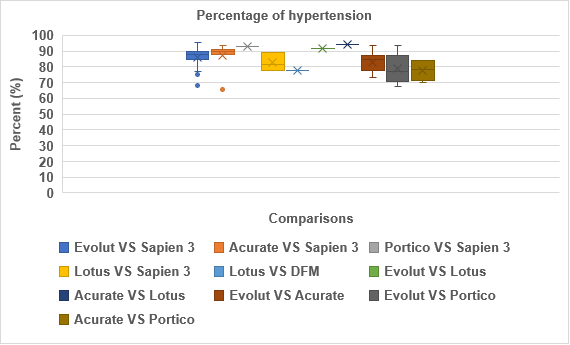 | 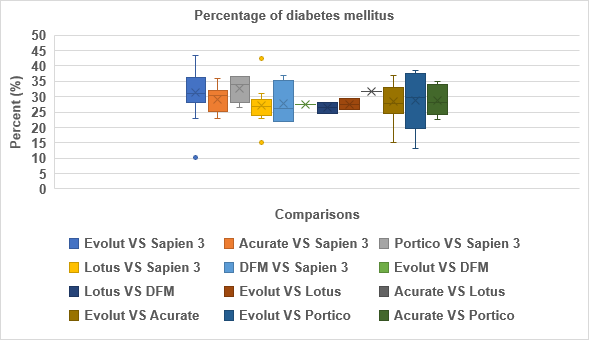 |
| 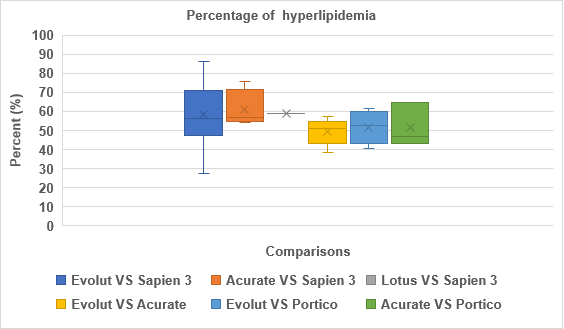 | 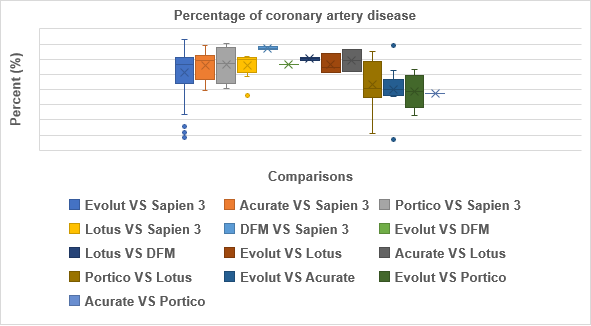 |
| 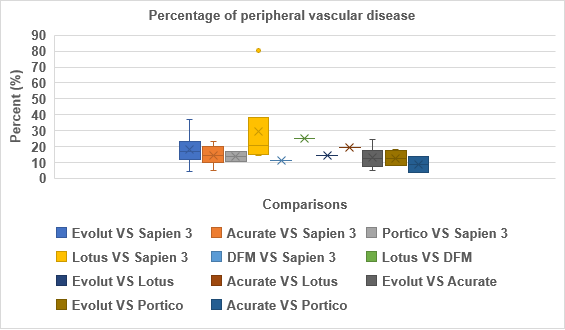 | 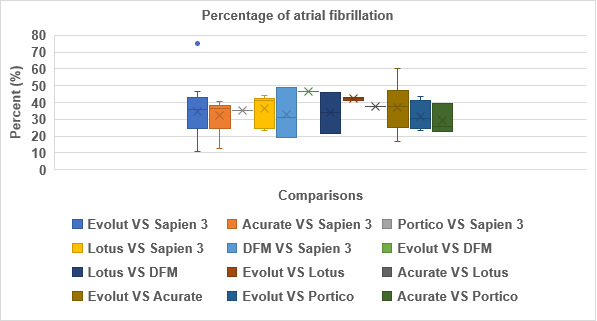 |
| 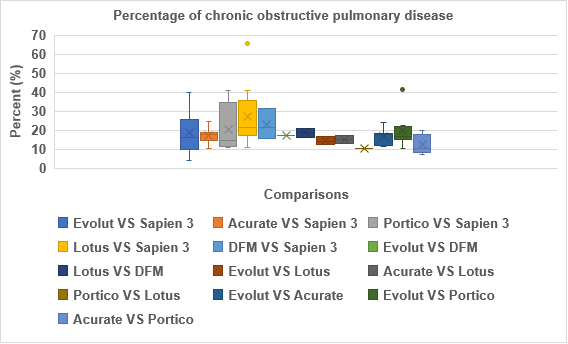 | 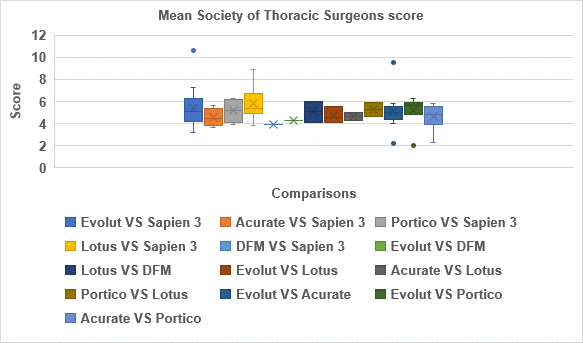 |
| 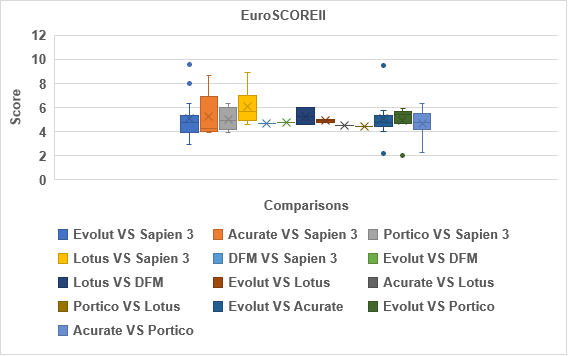 | 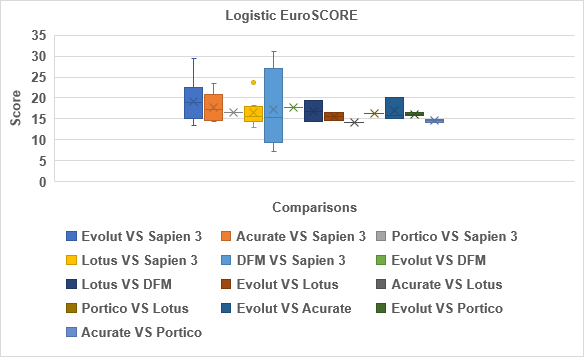 |
| 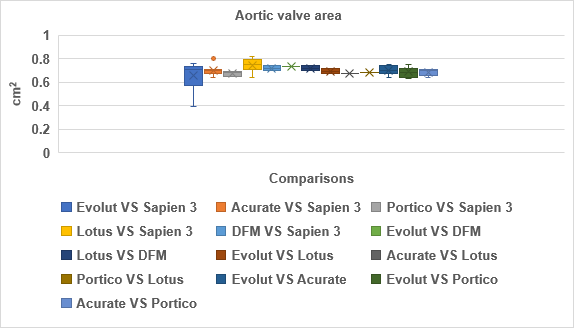 | 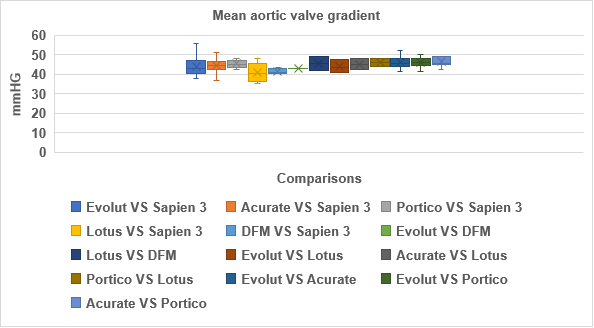 |
| 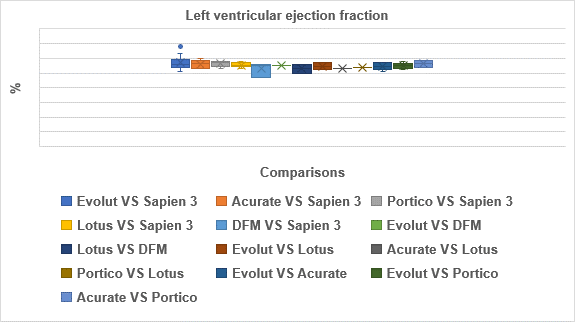 | 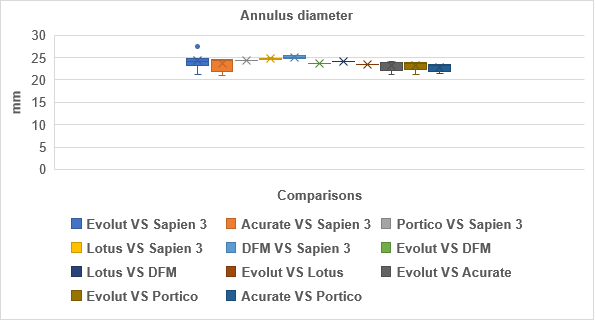 |
| 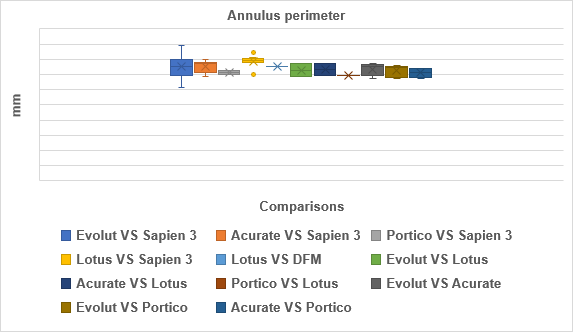 | 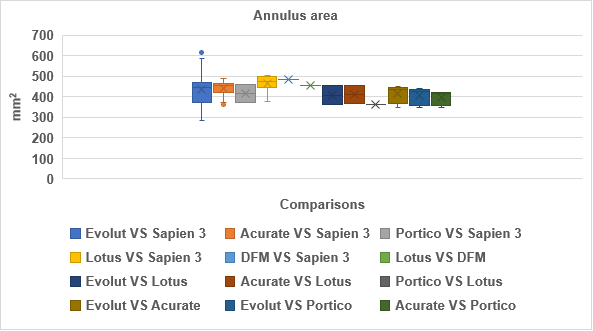 |
| 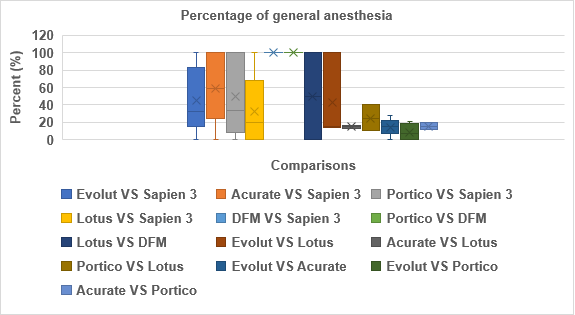 | 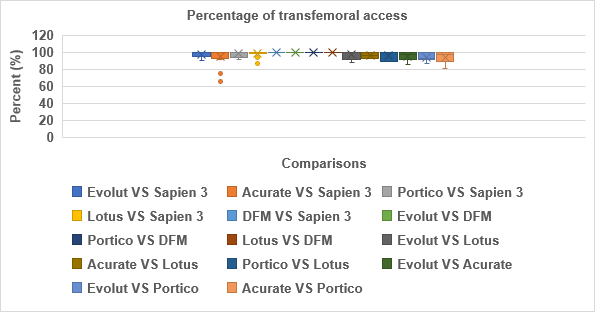 |
| 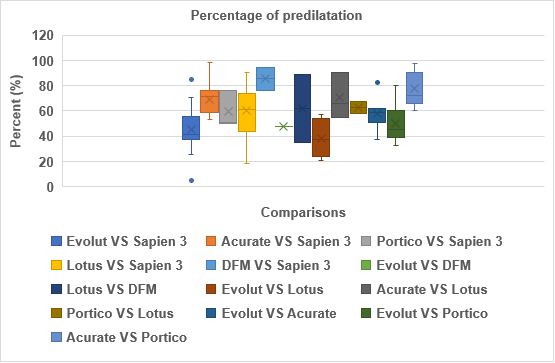 | 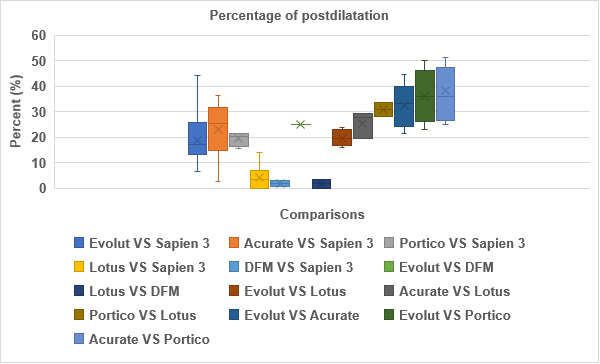 |

**Figure 2. Comparison-adjusted funnel plot for each endpoint**

| **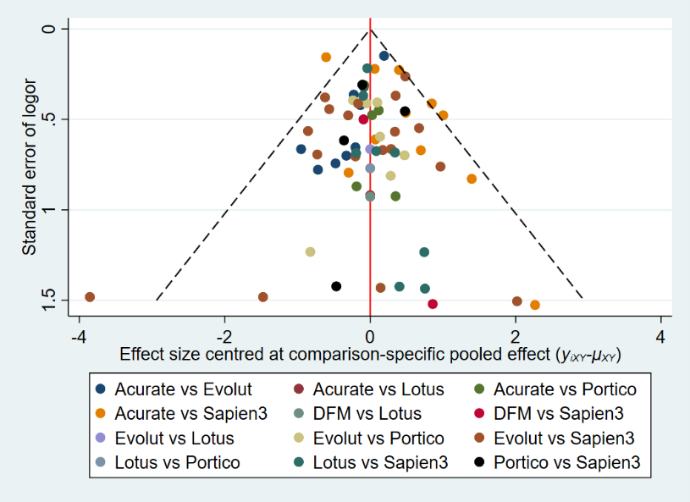** | **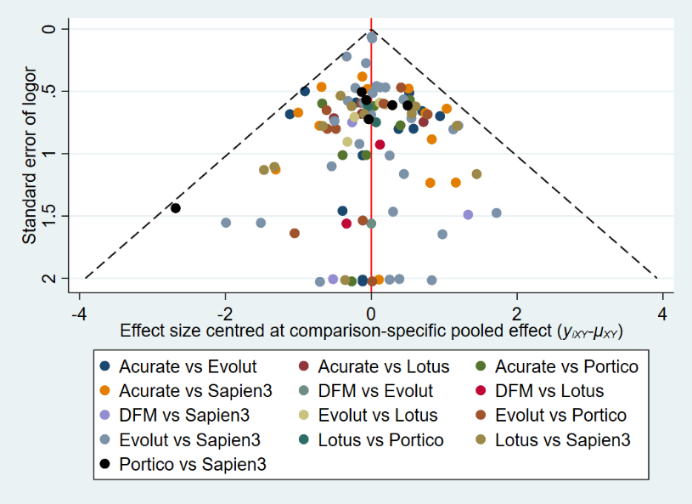** | **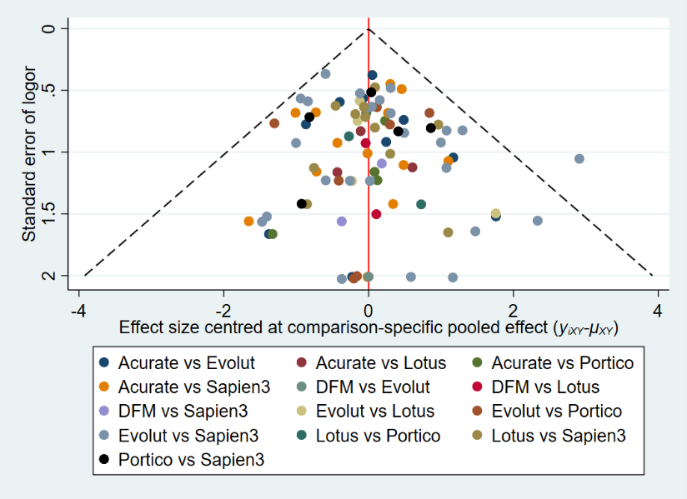** | **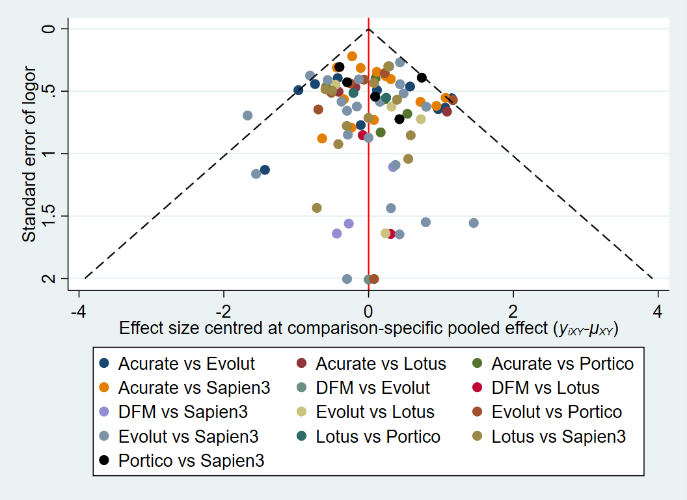** |
| --- | --- | --- | --- |
| **(A) Device success** | **(B) Mortality** | **(C) Stroke** | **(D) Major/life threatening bleeding** |
| 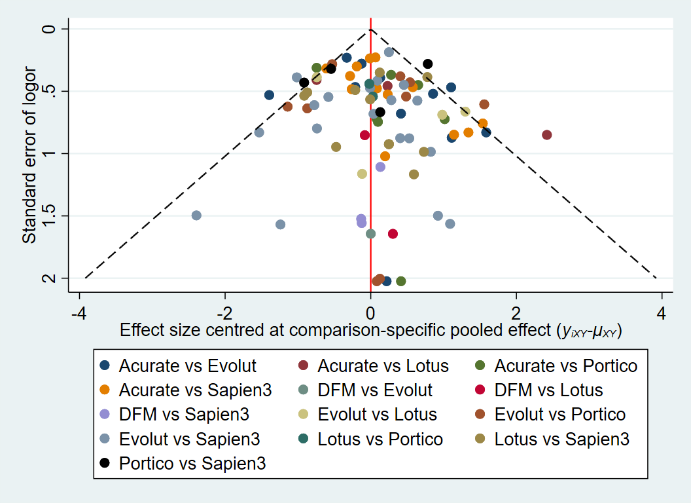 | 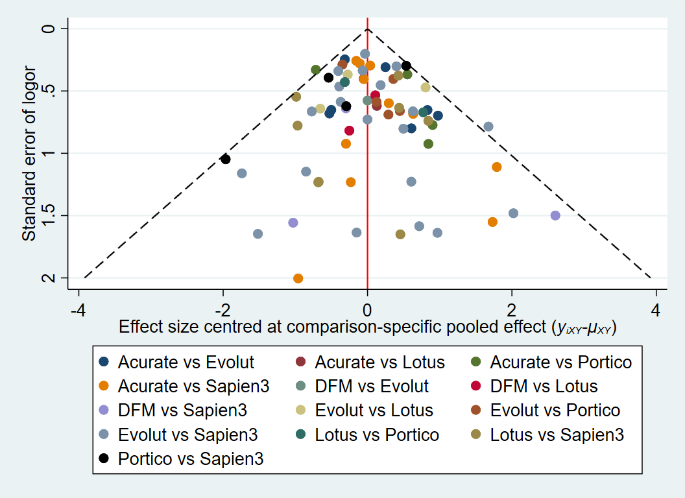 | 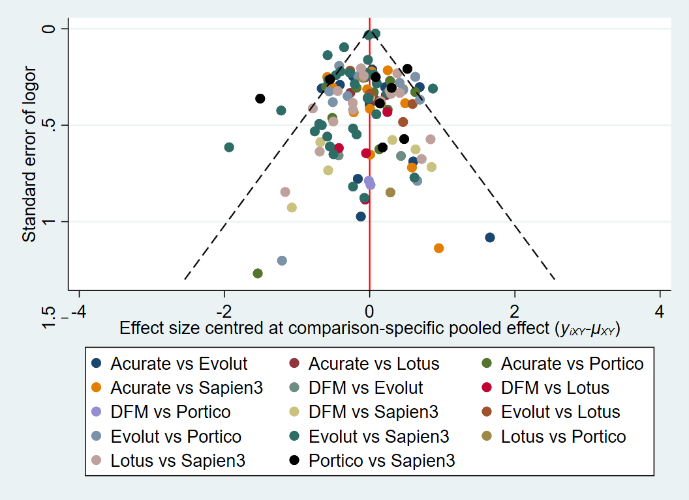 | 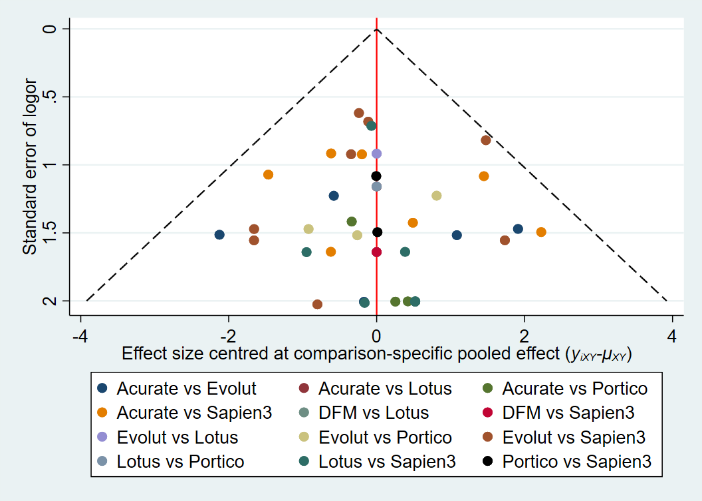 |
| **(E) Major vascular complication** | **(F) Acute kidney injury** | **(G) Permanent pacemaker implantation** | **(H) Procedural mortality** |
| **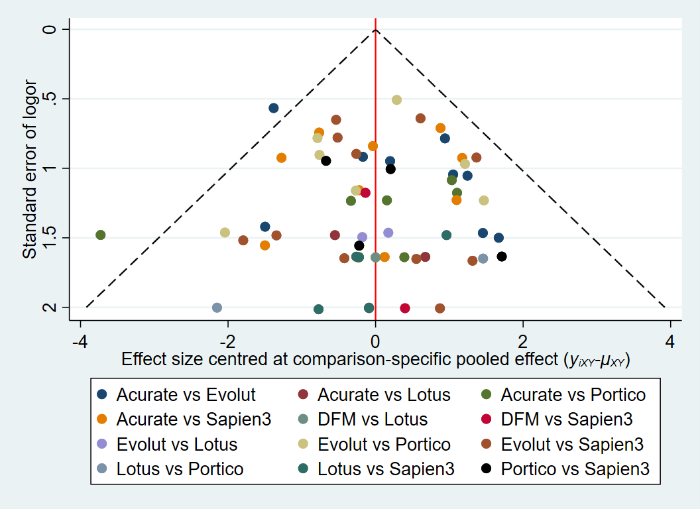** | 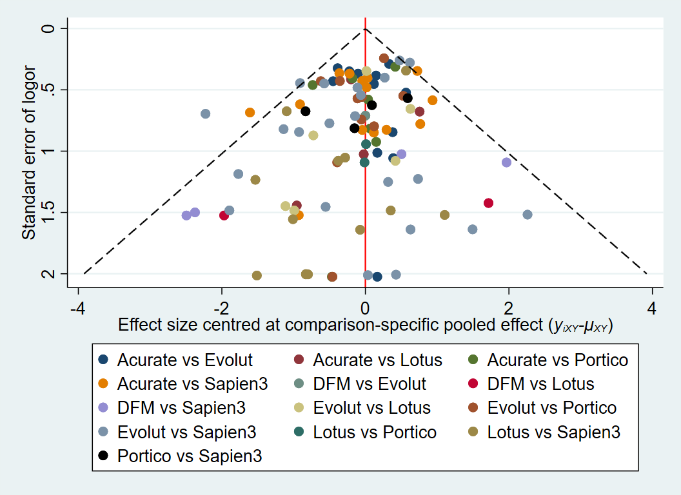 | **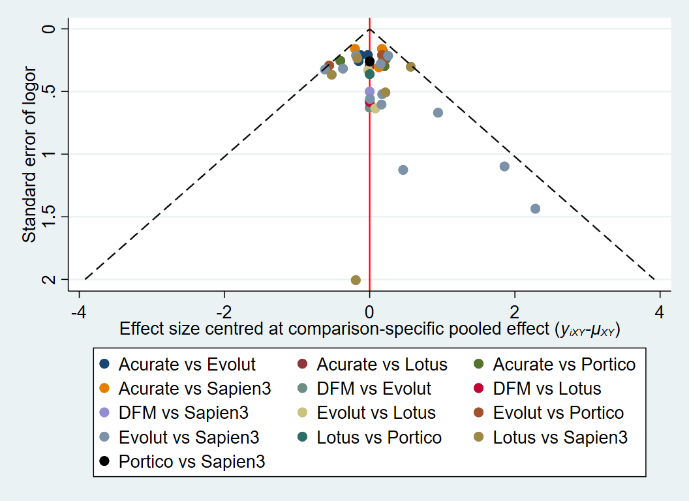** | 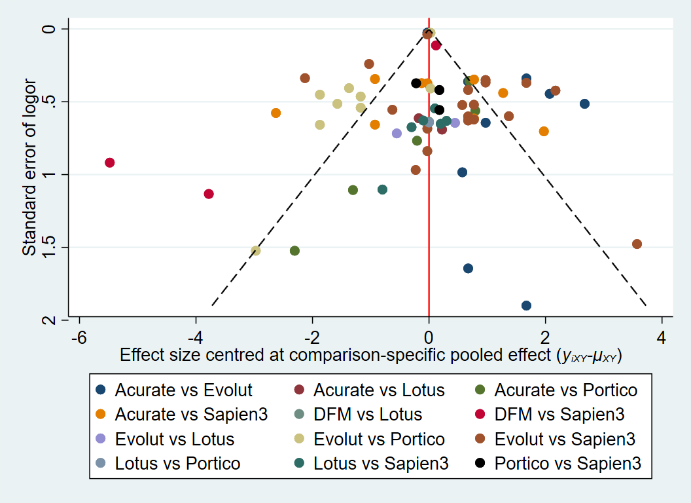 |
| **(I) No correct positioning** | **(J) Moderate-to-severe paravalvular leak** | **(K) Prosthesis patient mismatch** | **(L) Mean aortic valve gradients** |

**Figure 3. Cumulative ranking plots for each endpoint**

| **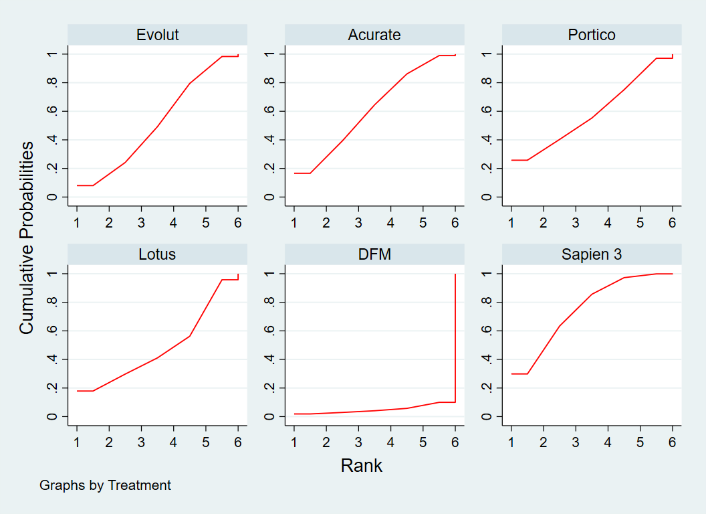** | **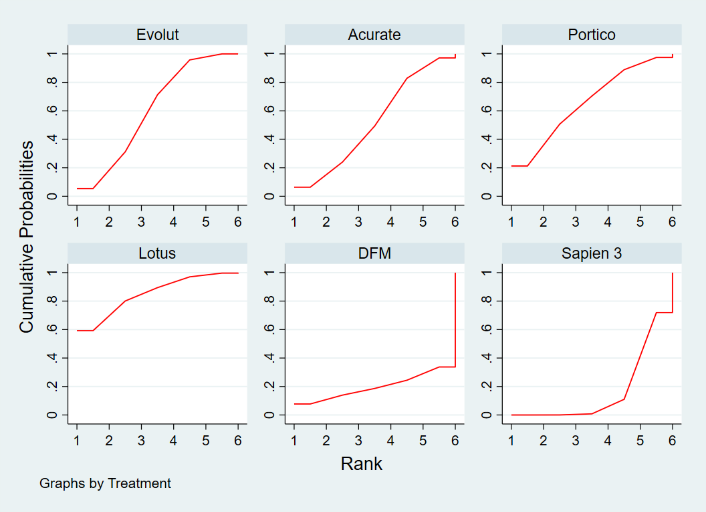** | **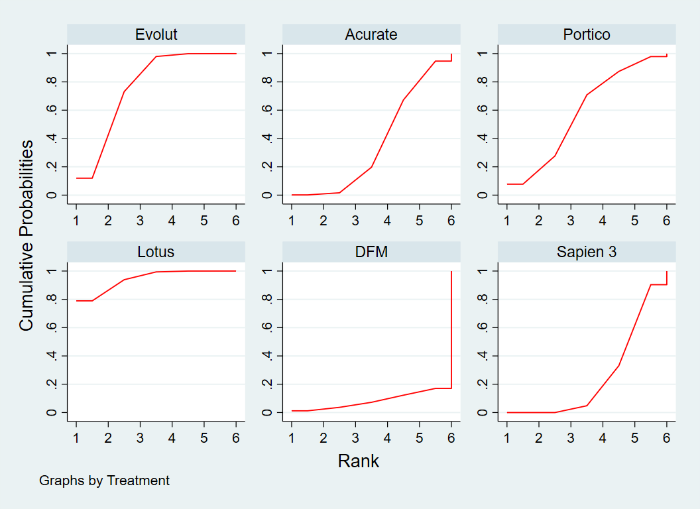** | **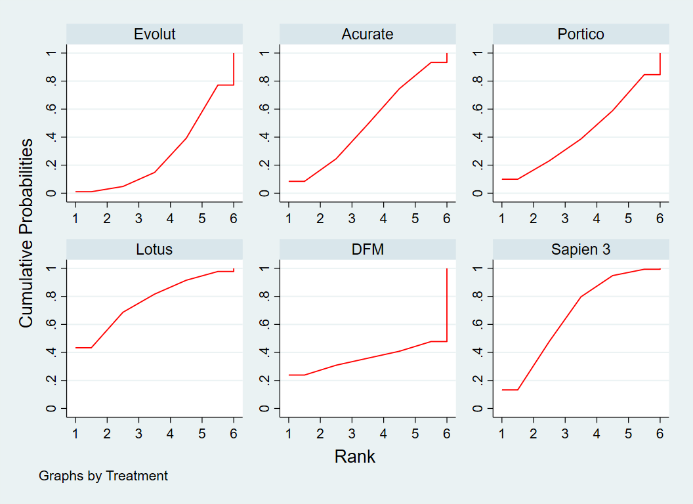** |
| --- | --- | --- | --- |
| **(A) Device success** | **(B) Mortality** | **(C) Stroke** | **(D) Major/life threatening bleeding** |
| **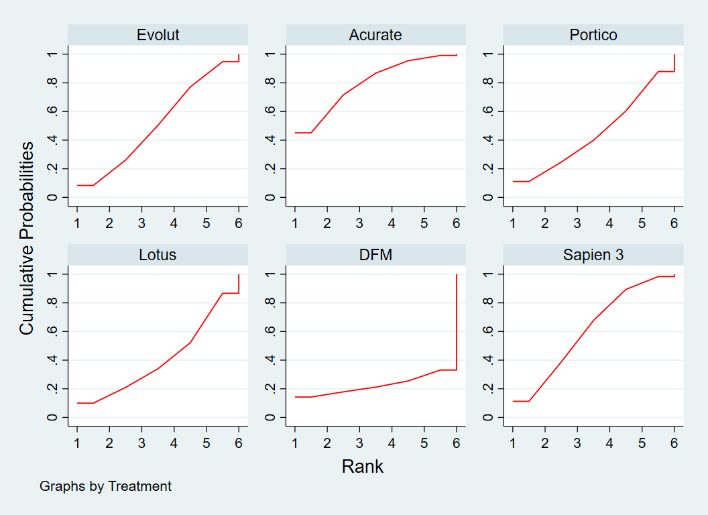** | **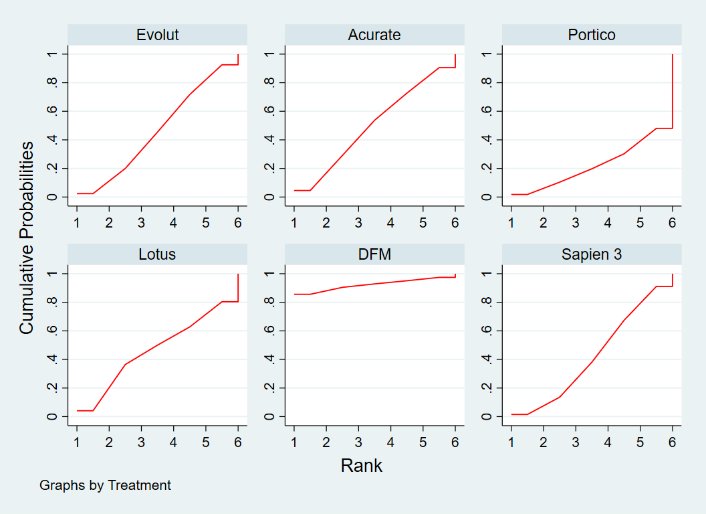** | **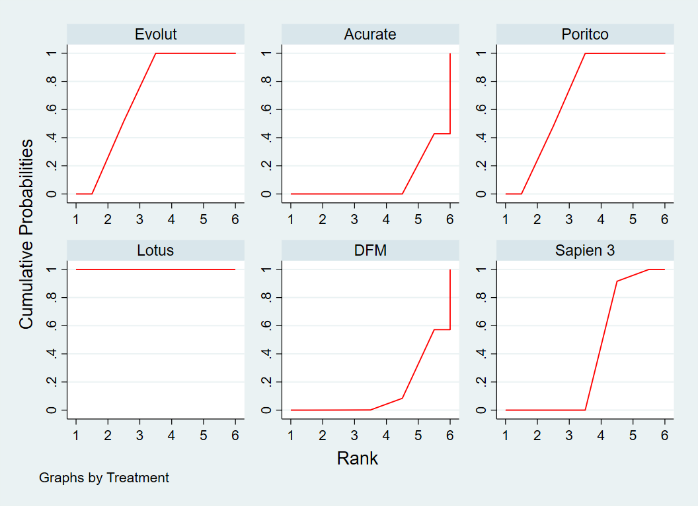** | **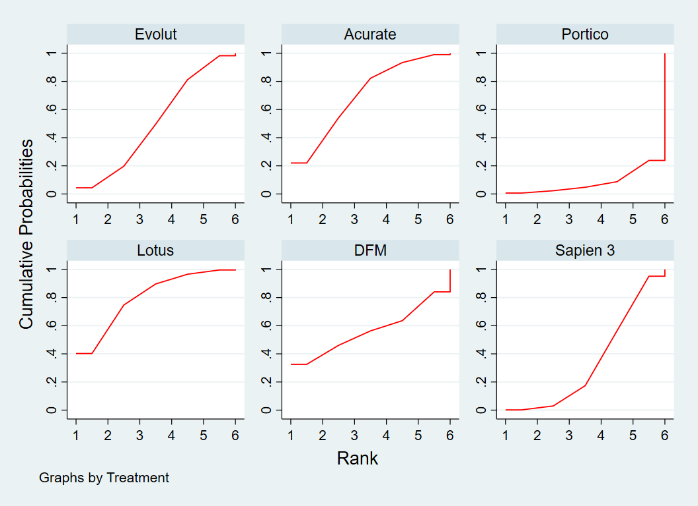** |
| **(E) Major vascular complications** | **(F) Acute kidney injury** | **(G) Permanent pacemaker implantation** | **(H) Procedural mortality** |
| **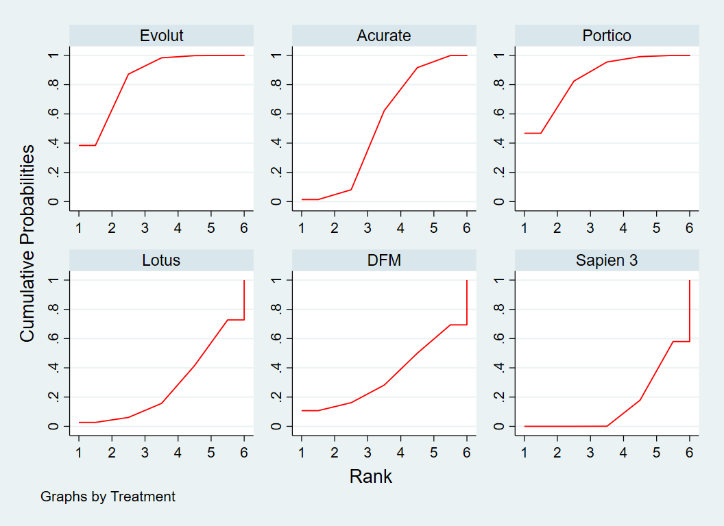** | **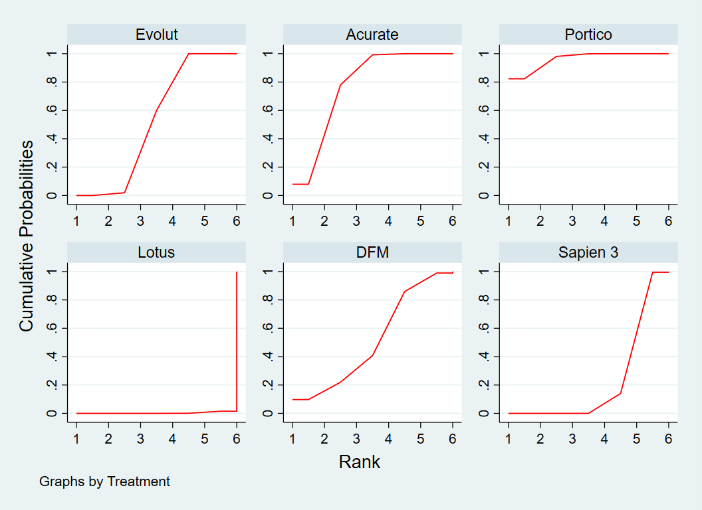** | **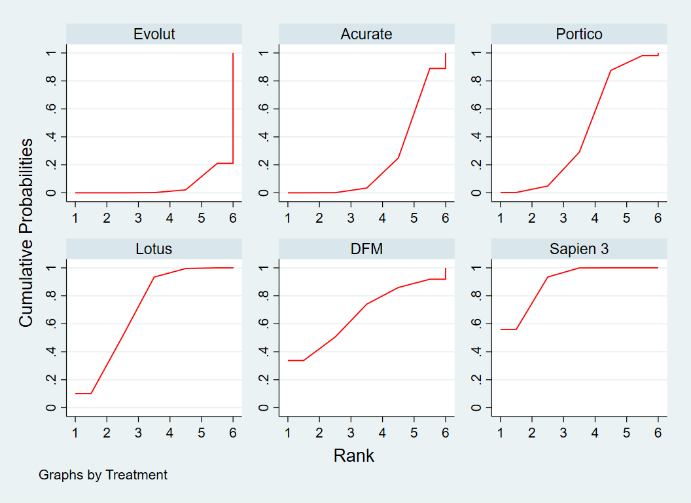** | **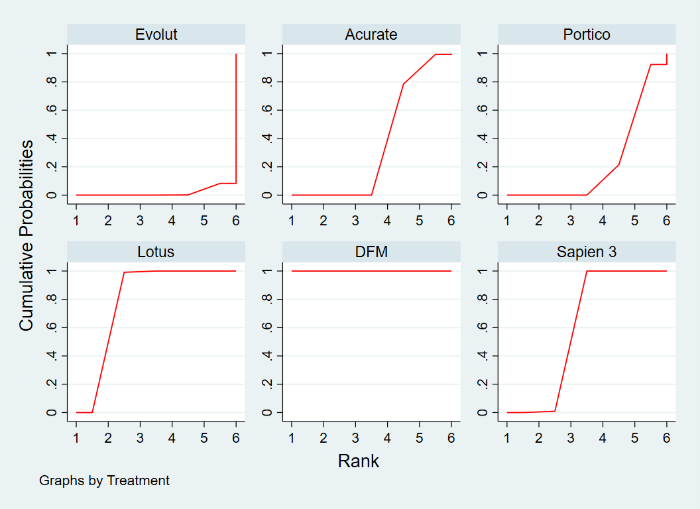** |
| **(I) No correct positioning** | **(J) Moderate-to-severe paravalvular leak** | **(K) Prosthesis patient mismatch** | **(L) Mean aortic valve gradients** |

**Figure 4. Contribution matrix for each endpoint**

| **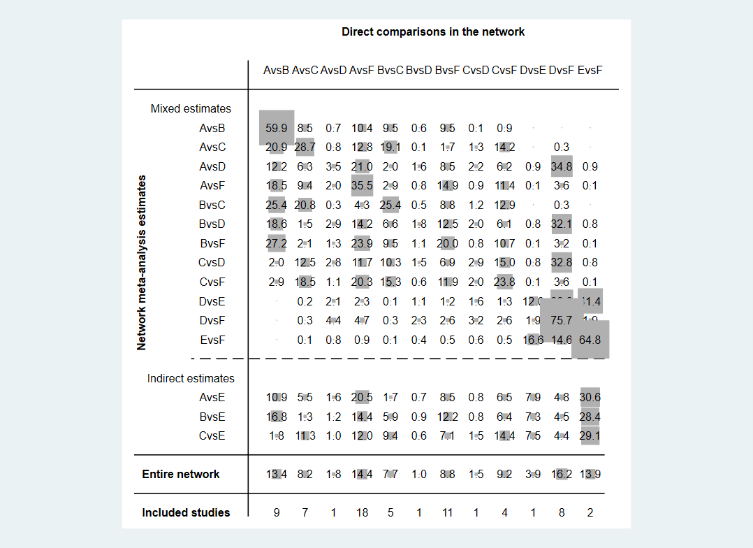** | **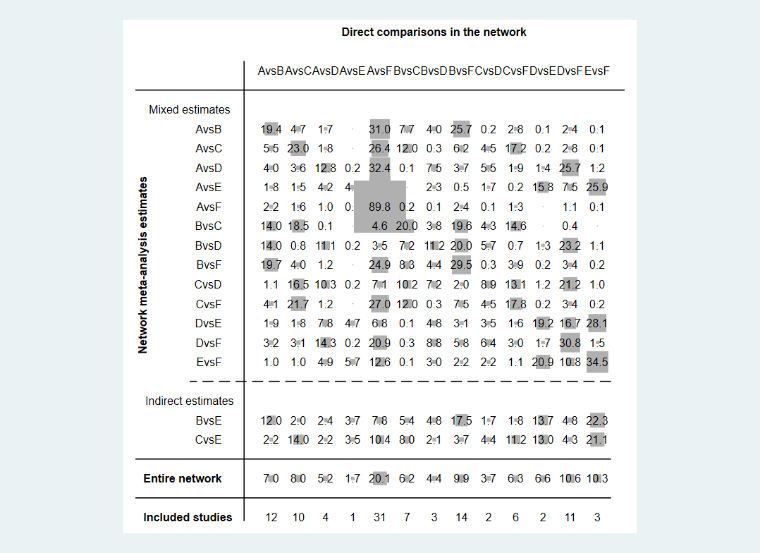** | **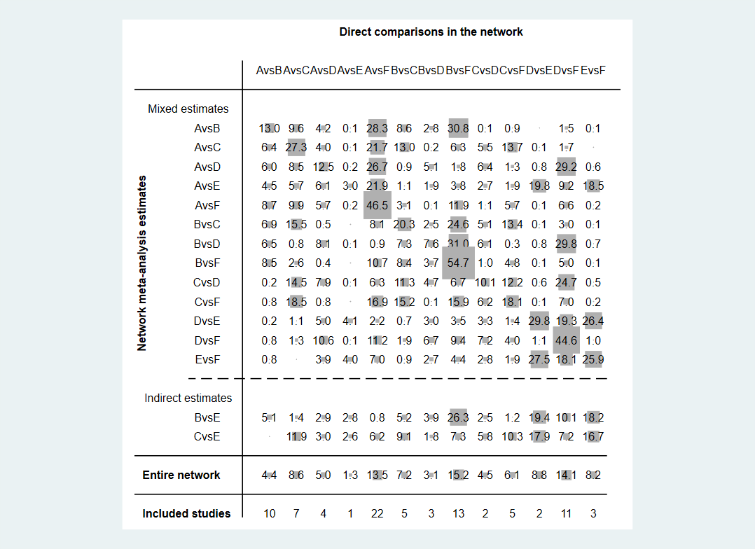** | **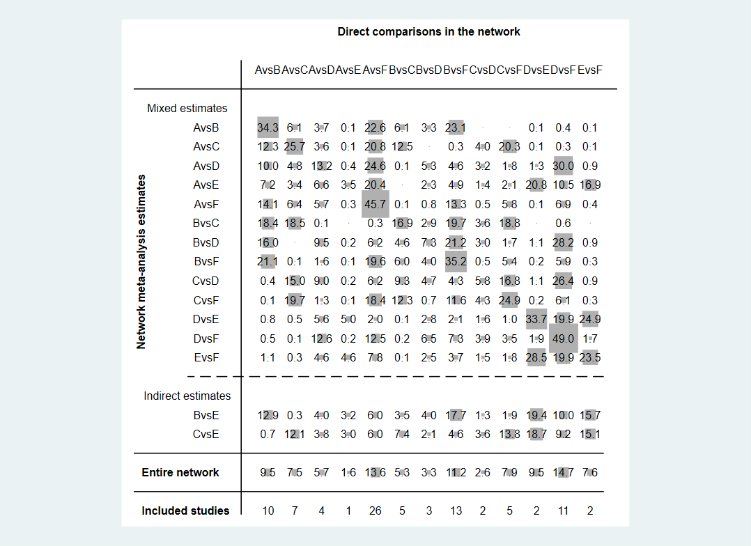** |
| --- | --- | --- | --- |
| **(A) Device success** | **(B) Mortality** | **(C) Stroke** | **(D) Major/life threatening bleeding** |
| **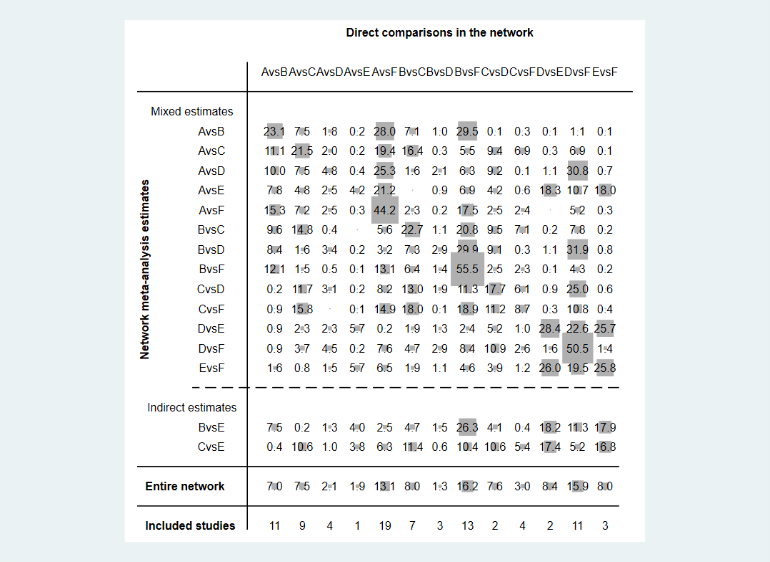** | 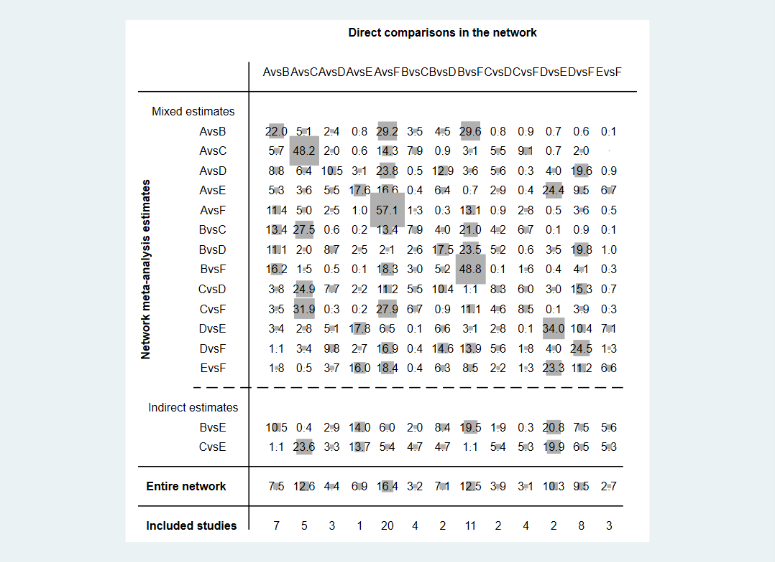 | **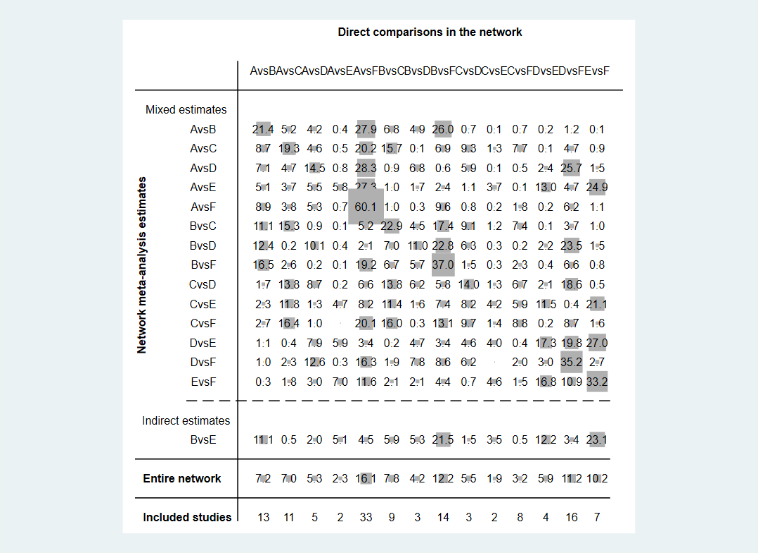** | **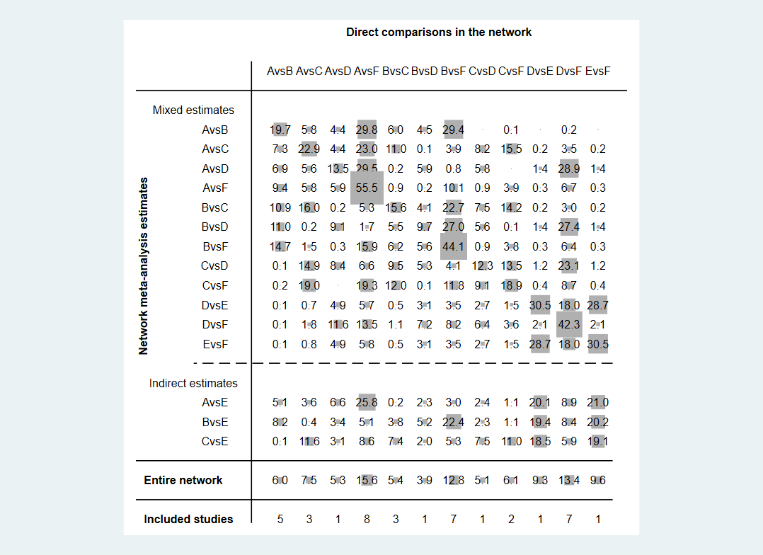** |
| **(E) Major vascular complications** | **(F) Acute kidney injury** | **(G) Permanent pacemaker implantation** | **(H) Procedural mortality** |
| _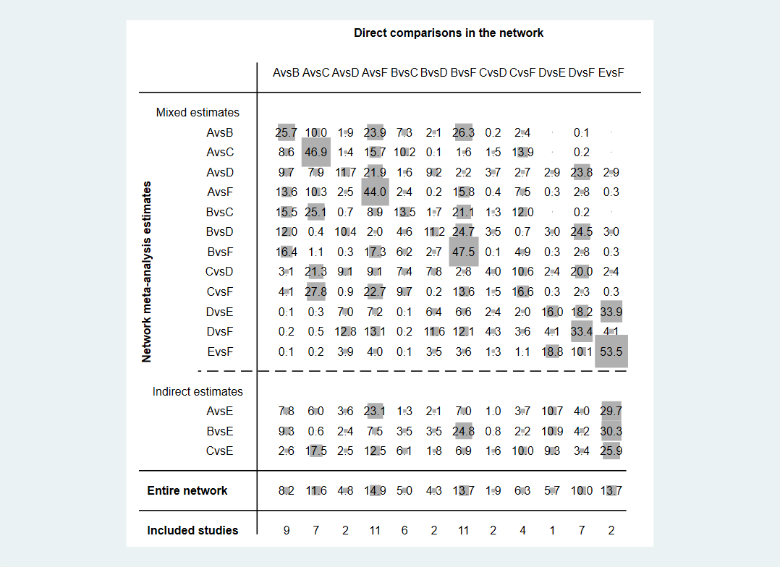_ | _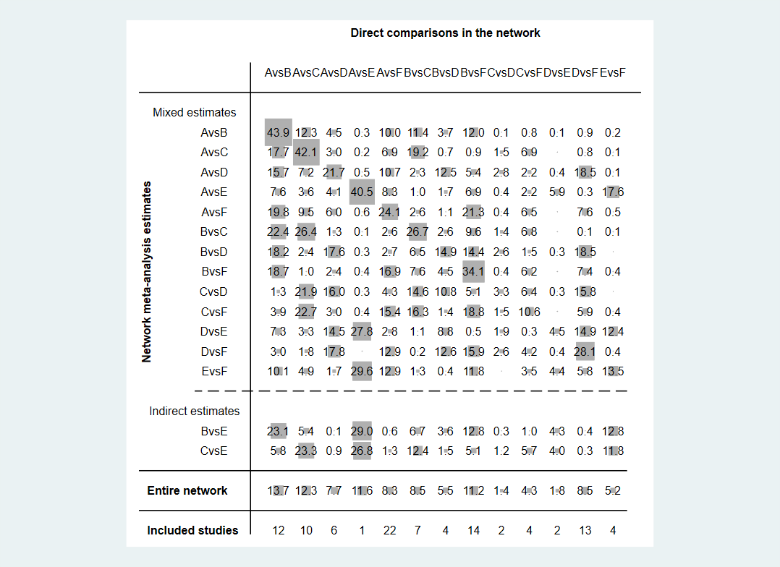_ | _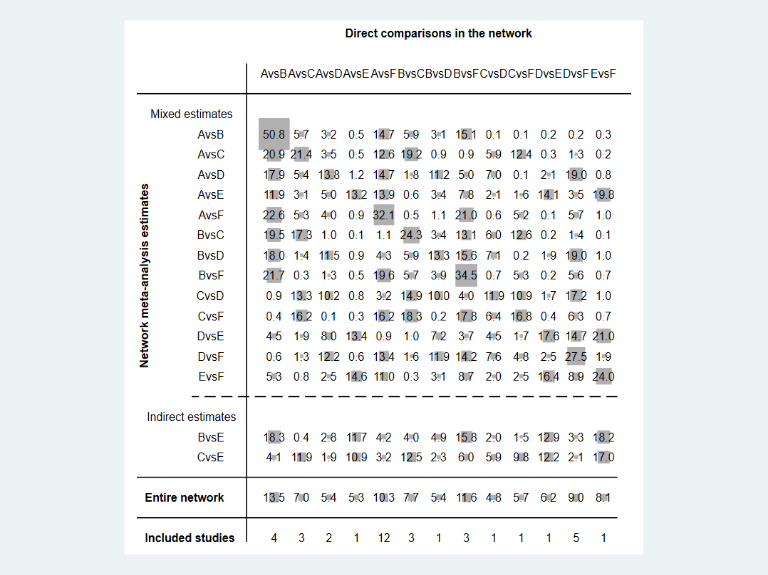_ | _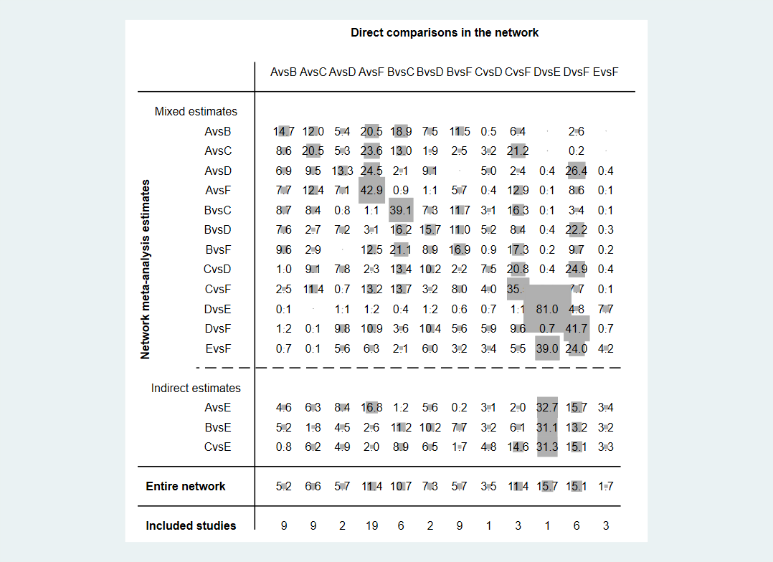_ |
| **(I) No correct positioning** | **(J) Moderate-to-severe paravalvular leak** | **(K) Prosthesis patient mismatch** | **(L) Mean aortic valve gradients** |

**Figure 5. 95% Prediction interval for each endpoint**

| 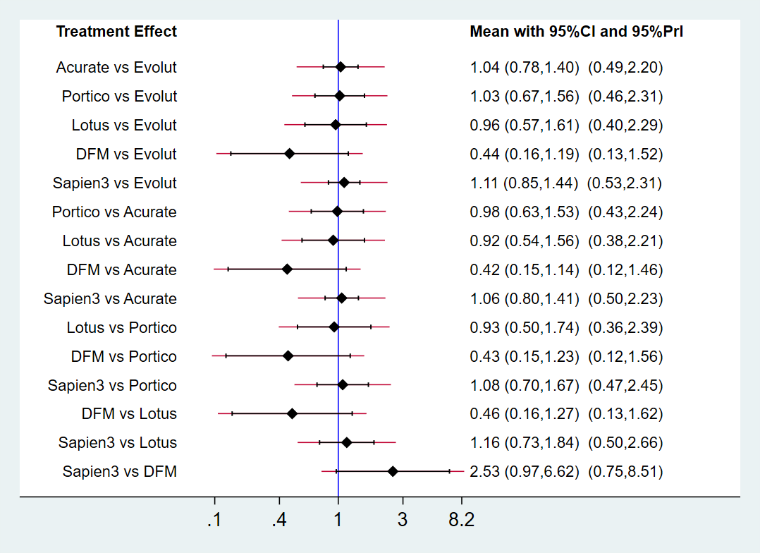 | 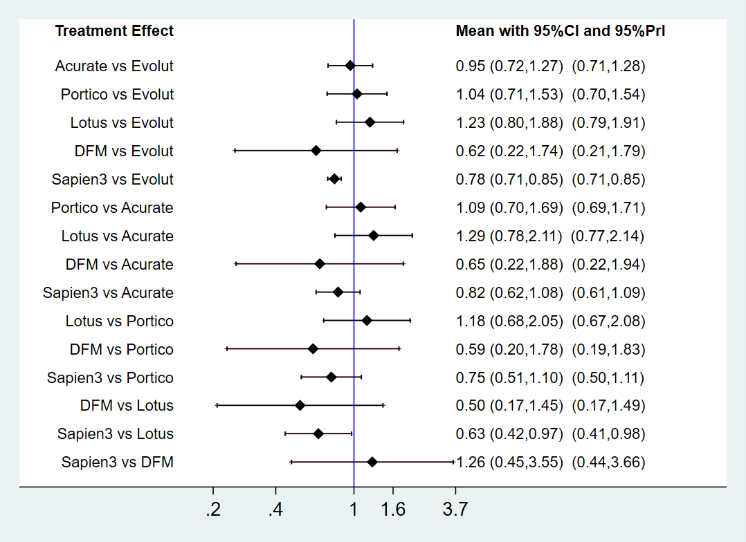 | 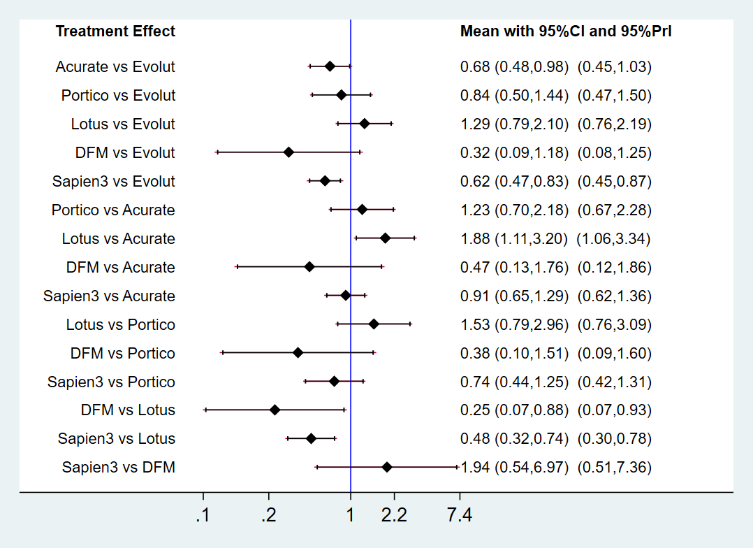 | 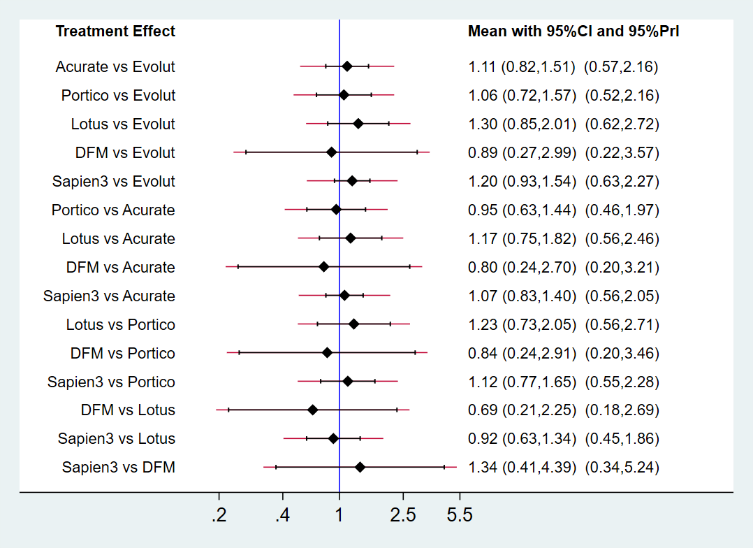 |
| --- | --- | --- | --- |
| **(A) Device success** | **(B) Mortality** | **(C) Stroke** | **(D) Major/life threatening bleeding** |
| 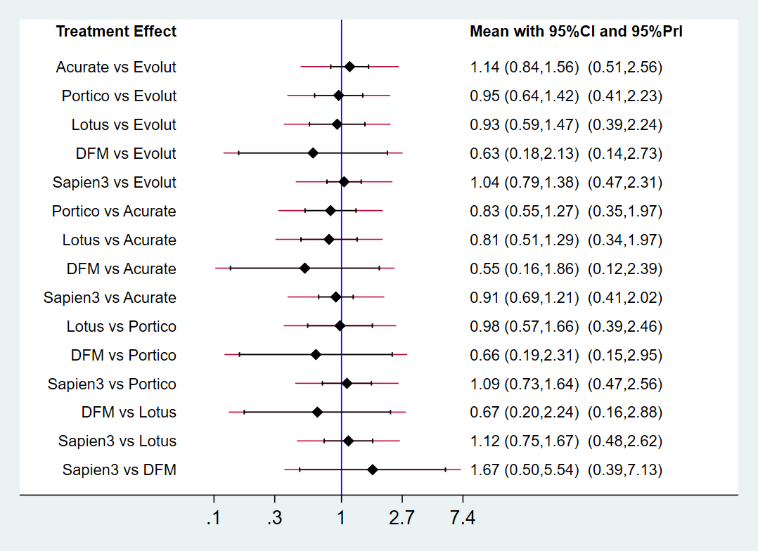 | **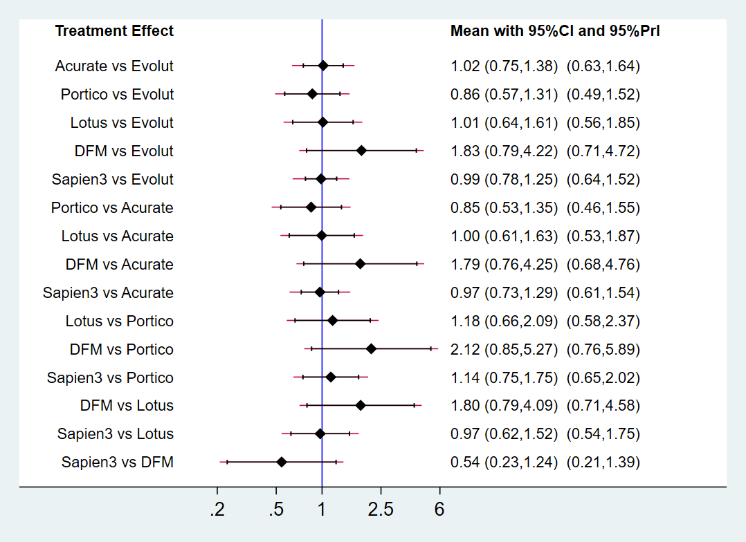** | **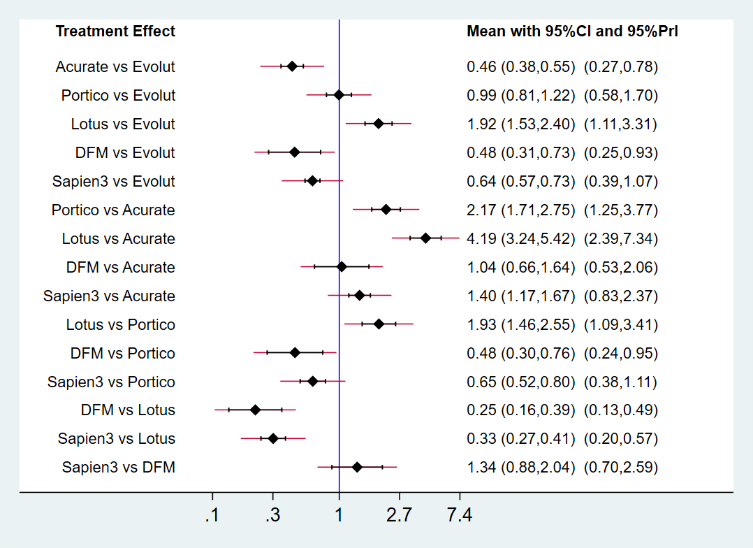** | 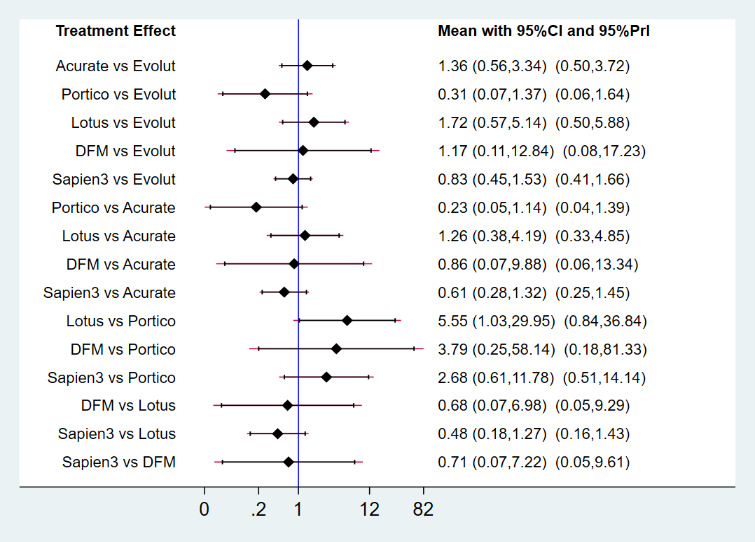 |
| **(E) Major vascular complications** | **(F) Acute kidney injury** | **(G) Permanent pacemaker implantation** | **(H) Procedural mortality** |
| **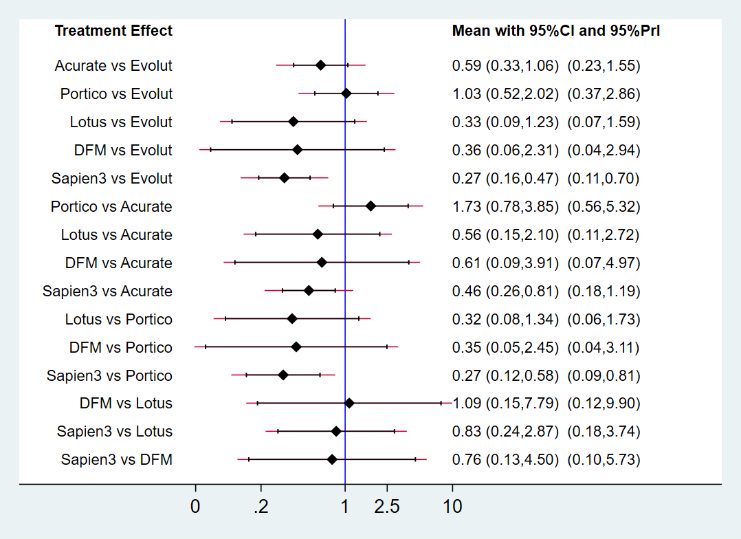** | 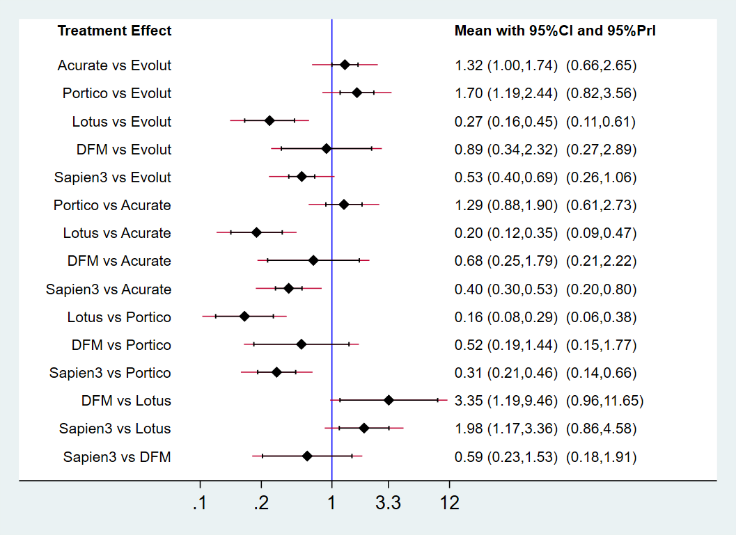 | 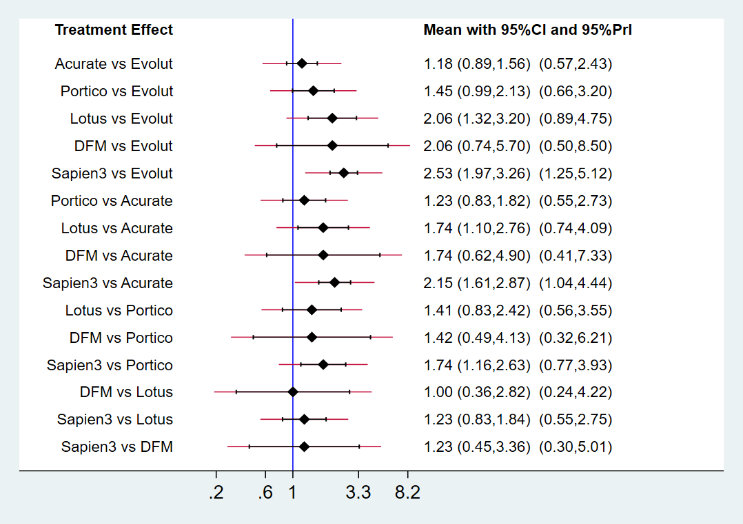 | 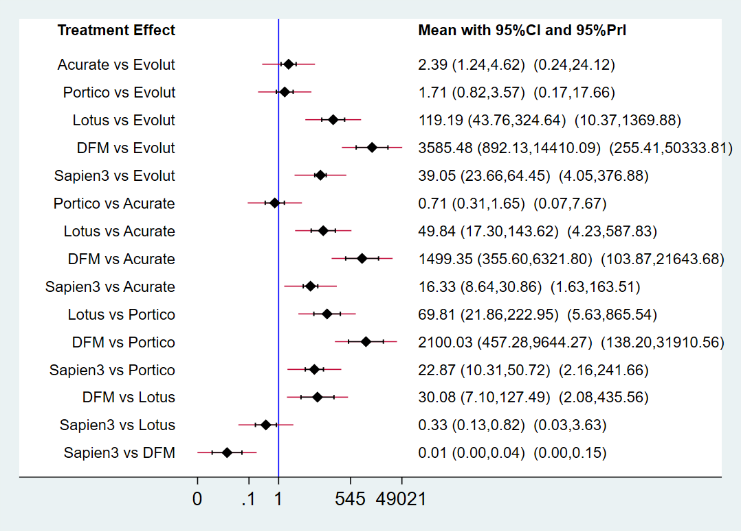 |
| **(I) No correct positioning** | **(J) Moderate-to-severe paravalvular leak** | **(K) Prosthesis patient mismatch** | **(L) Mean aortic valve gradients** |

**Figure 6. Summary of study limitations for each endpoint.**

|  |  |
| --- | --- |
|  |  |
|  |  |
|  |  |

|  |  |
| --- | --- |
|  |  |

Each bar corresponds to a NMA relative treatment effect and shows how much information comes from comparisons at low risk of bias (green] or moderate risk of bias.
